# Mental health in adults aged 50+ since the COVID-19 pandemic: Are we (all) back to ‘normal’? evidence from England

**DOI:** 10.1016/j.jadr.2025.101012

**Published:** 2026-01-23

**Authors:** Darío Moreno-Agostino, Giorgio Di Gessa

**Affiliations:** a Centre for Longitudinal Studies, UCL Social Research Institute, University College London, London, UK; b ESRC Centre for Society and Mental Health, King’s College London, London, UK; c Department of Epidemiology and Public Health, University College London, London, UK

**Keywords:** Depression, Anxiety, Loneliness, Quality of life, English Longitudinal Study of Ageing, Inequalities, Gender, Wealth, Living Arrangements

## Abstract

**Objectives:**

To understand how population mental health levels and inequalities in these are in the post-lockdown world compared to before the pandemic in adults aged 50 and older.

**Methods:**

We used data from three Waves (2016–2017, *n* = 7191; 2018–2019, *n* = 7286; and 2021–2023, *n* = 6249) of the English Longitudinal Study of Ageing. Using linear and modified Poisson regression models, we investigated whether prevalence of high depressive symptomatology, anxiety, and loneliness, and quality-of-life levels changed across time points overall and by gender, living situation, and wealth quintiles. Models were adjusted for age group, gender, education, and long-standing illnesses.

**Results:**

No significant differences were found between 2016–2017 and 2018–2019. However, compared to 2018–2019, prevalence of high depressive symptoms (RR_2021–2023_ = 1.23[95 %CI 1.12;1.34], *p* < 0.001), loneliness (RR_2021–2023_ = 1.32[1.22;1.42], *p* < 0.001) and quality-of-life levels (B_2021–2023_ = −1.84 [−2.21;−1.48], *p* < 0.001) were worse by 2021–2023. Pre-existing inequalities by gender, living arrangements, and wealth were not significantly different after the pandemic, except for depression, where gaps were significantly smaller by gender (RR_2021–2023*women_ = 0.72[0.59;0.89], *p* = 0.002) and, to a smaller extent, living situation (RR_2021–2023*not_alone_=1.22[1.02;1.47], *p* = 0.026).

**Conclusion:**

Population mental health levels in the population aged 50 and older seem to have declined after the pandemic, and inequalities within the population persist.

## 1. Introduction

The COVID-19 pandemic has had a profound impact on mental health globally. Despite some heterogeneity in results due to different methodologies, populations under study, and measures of mental health considered, reviews and meta-analyses report an increase in the prevalence of psychological distress, anxiety, and depression, particularly at the pandemic onset (Necho et al., 2021; Patel et al. 2022; Robinson et al. 2022; Santomauro et al. 2021). The World Health Organization (2022) reported a 25 % increase in the global prevalence of depression and anxiety throughout 2020, the first year of the pandemic. Much of these detrimental effects on mental health and well-being have been explained by significant direct and indirect effects and disruptions that the COVID-19 pandemic and policies to contain the spread of the disease and save lives have had on people’s lives. These included (but were not limited to) lockdowns and social distancing, closures of educational institutions, community facilities (e.g. libraries), and all non-essential shops and services, job losses and increased poverty (Brooks et al. 2020; Ebrahim et al. 2020; Holmes et al. 2020; Kumar and Nayar 2021; Nicola et al. 2020; Pfefferbaum and North 2020). Moreover, the high mortality of the SARS-Cov-2 virus paired with reduced access to, and the delivery of, primary and specialist health and social care services triggered anxiety and depression, both among people at higher risk because of their own age or clinical profiles (Mansfield et al. 2021; Murphy et al. 2021) and those individuals who lost family members and friends to COVID-19 (Joaquim et al. 2021).

The pandemic also exacerbated existing inequalities in mental health (Bambra et al. 2020; Marmot et al. 2020; Parenteau et al. 2023). Although upward trends of psychological distress and poorer mental health were observed since the onset of the pandemic, this overall effect was not distributed equally in the population. Women reported a larger deterioration in mental health since the COVID-19 pandemic than men (Niedzwiedz et al. 2020; Patel et al. 2022; Zaninotto et al. 2022). Also, although increased psychological distress was most prominent among younger adults (Moreno-Agostino et al. 2023b; Niedzwiedz et al. 2020; Patel et al. 2022), changes in mental health and well-being were not uniform across older age groups either (Di Gessa and Price 2021). The pandemic also impacted socioeconomic groups differentially, often increasing the gap in mental health distress among those better and worse off (Gibson et al. 2021; Parenteau et al. 2023). Also, the health and well-being of vulnerable populations, including those with pre-existing mental health conditions, were disproportionately affected by the pandemic (Di Gessa and Price 2021, 2022; Moreno-Agostino et al. 2023a; Zhu, Zaninotto and Di Gessa 2023; Steptoe and Di Gessa, 2021).

The approval and authorisation by health authorities to use COVID-19 vaccines changed the course of the pandemic (Watson et al. 2022). As vaccination programmes started to roll out as early as December 2020, non-pharmaceutical interventions that included lockdowns and social distancing started to be lifted at the pace of vaccination, particularly among older people and individuals at higher risk of mortality and hospitalisation due to COVID-19 (Bauer et al. 2021; Ge et al. 2022). For example, restrictions in England were mainly lifted on 19 July 2021, when the coverage rate of vaccination in adults aged ≥18 in the UK was about 88 % for dose 1 and 70 % for dose 2 of COVID-19 vaccines (Song and Bachmann 2021). Since then, the WHO has declared the end of a global health emergency (Wise 2023) and most societies have returned to what has been referred to as (pre-COVID-19) “normality”. However, whether this also meant that mental health and well-being bounced back to pre-pandemic levels remains understudied, and it remains unclear whether some of the increased inequalities persisted after the COVID-19 pandemic.

To date, the evidence seems to suggest that mental health and well-being have improved since the onset of the COVID-19 pandemic, returning to levels similar to those observed pre-pandemic. These patterns of mental health and well-being have broadly mirrored rates of infections and mortality, patterns of non-pharmaceutical restrictions (Aknin et al. 2022; Easterlin and O’Connor 2023; Smith et al. 2022; Zacher and Rudolph 2024), and the roll-out of COVID-19 vaccination (Perez-Arce et al. 2021; Walker et al. 2024; Zhai et al. 2024), suggesting that those vaccinated might have been less worried about infection and death and more socially active. Declines in life satisfaction in Europe were found to be associated with increases in the severity of the pandemic (i.e. waves of infections and COVID-19 death rates), and poorer mental health in the first 18 months of the pandemic was associated with more stringent COVID-19 policies. A longitudinal study of German full-time employees found that life satisfaction returned to pre-pandemic levels after restrictions were almost completely lifted in early April 2022 (Zacher and Rudolph 2024). Also, a study based on a longitudinal sample of older English people found that positive well-being levels in late 2021/23 bounced back and were even greater than those observed in the pre-pandemic period, although depression was still higher (Zaninotto et al. 2025). However, so far, the evidence as to whether the prevalence of poor mental health and well-being is back to pre-pandemic levels remains scarce and has focused on life satisfaction and positive well-being, overlooking other important aspects such as anxiety, loneliness, and quality of life (QoL). Also, it remains unknown whether the sociodemographic inequalities in mental health and well-being observed during the COVID-19 pandemic have returned to the levels observed pre-2020 at the population level.

In light of this, the present study aims to describe whether and to what extent overall levels of mental health post-COVID-19 pandemic (namely, depressive symptoms, anxiety, loneliness, and QoL) have reverted to levels observed before the outbreak. Second, we will assess inequalities in post-pandemic mental health and test whether these are similar to the gaps observed in the years preceding the pandemic. In particular, we focus on people aged 50 and older. Soon after the COVID-19 outbreak, age and disease profiles independently predicted the severity of illness and mortality from COVID-19 (Iaccarino et al. 2020). As the onset and prevalence of chronic conditions and multimorbidity emerge in mid-life and are higher in older ages (Airoldi et al. 2023; Gondek et al. 2021), older people with underlying health conditions were the focus of policy recommendations to stay indoors, limit travels and movements, as well as limit physical interactions with others during the pandemic. Moreover, as described in the Method section, we use a study that collects data from people aged 50 and older. Overall, using multiple waves of nationally representative datasets that collect information pre- and post-COVID-19 pandemic, we aim to understand whether we are “back to normal” (i.e., to the levels observed in the years preceding the COVID-19 pandemic) when it comes to mental health and well-being, and whether we are also back to observing the well-known health inequalities.

## 2. Materials and methods

### 2.1. Study design and population

We used data from the English Longitudinal Study of Ageing (ELSA), a long-term survey initiated in 2002 that is representative of individuals aged 50 and older who live in private households in England (Banks et al. 2024). In its initial round of data collection in 2002/03 (known as Wave 1), ELSA originally included 11,391 core participants, selected through the Health Survey for England to accurately reflect the demographics of older English adults. ELSA has periodically refreshed its sample with new participants to ensure the study remains representative of those aged 50 and over (Steptoe et al. 2012). Since its inception, ELSA has collected information biennially, with the sequence interrupted by the COVID-19 pandemic and the latest available data (Wave 10) collected post-pandemic between October 2021 and March 2023 (with a combination of CAPI face-to-face and CAWI video interviews). Additional information about the survey’s sampling frame, methodology, and ethical approvals is available at https://www.elsa-project.ac.uk. All participants gave informed consent. All data can be accessed through the UK Data Service (SN 5050). For this study, we considered all cross-sectional non-proxy core members of three rounds of data collection: Wave 8 (May 2016 – May 2017; *N* = 6889), Wave 9 (July 2018 – June 2019; *N* = 6940), and Wave 10 (October 2021 – March 2023; *N* = 6041). The sample for some of the outcomes considered (anxiety, loneliness, and QoL) was smaller in all waves because they were assessed in the self-completion questionnaire, with response rates at around 90 % for all waves.

### 2.2. Health outcomes

We considered four outcome measures of mental (ill-)health and well-being: high depressive symptoms, high anxiety, high loneliness, and QoL. Symptoms of depression were measured by an 8-item version of the validated Centre for Epidemiologic Studies Depression (CES-D) Scale (Radloff 1977). The CES-D scale is not a diagnostic instrument for clinical depression, but can be used to identify people “at risk” of depression in population-based studies. This short version has shown good internal consistency, with Cronbach’s α and Omega total (*ω*_*t*_) values ≥0.90 (Schlechter, Ford and Neufeld 2023) and comparable psychometric properties to the full 20-item CES-D (Karim et al. 2015). The scale includes eight binary (no/yes) questions that ask whether respondents experienced any depressive symptoms, such as feeling sad or having restless sleep, in the week before the interview. In our study, the *ω*_*t*_ of the CESD-8 was 0.80 across waves. In line with previous studies, we classified respondents who reported four or more depressive symptoms on the CES-D scale as having elevated depressive symptoms (Turvey, Wallace and Herzog 1999; Zivin et al. 2010).

Anxiety was measured as part of the self-completion questionnaire using the UK Office for National Statistics (ONS) anxiety single question: “On a scale where 0 is ‘not at all anxious’ and 10 is ‘completely anxious’, overall, how anxious did you feel yesterday?”. We created a binary variable representing whether the person had experienced high anxiety or not, using the ‘high’ threshold of 6–10 as indicated in the ONS personal well-being user guidance (ONS Office for National Statistics, 2025).

Loneliness was also measured in the self-completion questionnaire using the short (three-item) revised version of the University of California, Los Angeles (R-UCLA) Loneliness Scale (Hughes et al. 2004), designed to measure loneliness without directly mentioning the word “loneliness”. The R-UCLA loneliness scale includes three questions: “How often do you feel you lack companionship?”, “How often do you feel left out?” and “How often do you feel isolated from others?”. Responses were scored from 0 (hardly ever or never) to 2 (often), with total scores ranging from 0 to 6 and higher values indicating greater loneliness. The scale has generally shown good measurement properties (Alsubheen et al. 2023). In our study, the *ω*_*t*_ of the UCLA-3 was 0.84 across waves. Although there is no agreed threshold score for the UCLA-3 loneliness scale, previous studies have often considered values ≥3 (i.e. values in the top quintile of the distribution) to represent high loneliness (Davies et al. 2021; Gale, Westbury and Cooper 2017; Steptoe et al. 2013).

Finally, QoL was assessed in the self-completion questionnaire using the 19-item Control, Autonomy, Self-realisation and Pleasure (CASP-19) scale, which was specifically designed for individuals in later life and used in a wide variety of ageing surveys (Hyde et al. 2003). CASP-19 contains 19 Likert-scaled questions measuring older people’s control and autonomy as well as self-realisation through pleasurable activities. CASP-19 sum scores range from 0 to 57, with higher scores indicating greater QoL. The questionnaire has shown good psychometric properties (Frias-Goytia et al. 2024). In our study, the *ω*_*t*_ of the CASP-19 was 0.90 across waves. Thanks to its more favourable distribution, the CASP-19 sum score was treated as a continuous outcome variable in the analyses.

### 2.3. Key demographic and socioeconomic variables and covariates

Health inequalities were analysed by gender, living situation, and wealth quintiles. Although ELSA collects information on respondent’s sex, we refer to ‘men’, ‘women’, ‘gender’ and ‘gender inequalities’ to avoid attributing any between-groups differences as essential, biological, or inherent traits or characteristics, as we consider any differences in these outcomes to (more) likely be reflective of differential socialisation and oppression across the life course (Moreno-Agostino et al. 2024). Living situation was based on the number of people living in the household with the respondent, recoded into alone vs not alone. Wealth was operationalised as the sum of all savings, investments, physical wealth, and housing wealth after subtracting all financial and mortgage debt, categorised into quintiles (see https://www.elsa-project.ac.uk/user-guides).

As covariates in our models, we considered age groups (50–59, 60–69, 70–79, or 80 and older); educational levels based on respondents’ highest educational attainment and recoded into low (up to lower secondary), middle (upper secondary), and high (university or above) following the International Standard Classification of Education (http://www.uis.unesco.org/); and long-term overall disability assessed with the Global Activity Limitation Instrument (Van Oyen et al. 2018), which categorises individuals into three groups (severely limited, limited but not severely, and not limited at all).

### 2.4. Analytical strategy

First, we provided weighted cross-sectional descriptives at each wave for each outcome and variable of interest. Then, we performed pooled modified Poisson (for high depression, anxiety, and loneliness) or linear (for QoL) regression models, first overall and then by gender, living situation, and wealth quintiles, including the interaction terms between the grouping variable and time (wave) to test whether any between-groups inequalities had changed over time. All models were adjusted for age group, gender, educational attainment, and long-term disability. The main models by gender, however, were not adjusted for educational attainment to avoid over-adjusting for variables potentially in the pathway (separate models were conducted where educational attainment was included to check the robustness of the conclusions to this decision). All analyses employed relevant cross-sectional weights and survey settings in order to restore representativeness to the target population (non-proxy core members of the ELSA study, aged 50 or above). These weights were separately derived for the main survey and the self-completion questionnaire, to account for the differential probability of participating in these (Pacchiotti, Hussey and Bennett 2021). Since the proportion of missing data was relatively small (all below 5 %, except for CASP-19 which ranged between 6.03–7.45 %, see Table 1), the main analyses were conducted under a complete-case approach relying on the data being missing completely at random (MCAR). However, analyses were also conducted using data imputed via multiple imputation by chained equations (MICE, 20 imputations), hence relying on the more flexible assumption of data being conditionally missing at random (MAR).

### 2.5. Robustness checks

To contain the spread of the COVID-19 Omicron variant, the UK reintroduced temporary restrictions roughly spanning between November 2021 and February 2022. Concerning this, the main data collection mode (face-to-face interviews) was supplemented with telephone and online interviews. Moreover, some participants had received the COVID-19 vaccine booster by the time they were interviewed, while others had not. Since all these factors may have played a role in the mental (ill-)health and well-being levels reported by the participants, we conducted additional analyses where participants interviewed during a period of reinstated restrictions, without a face-to-face interview, or without a vaccine booster, were excluded. De facto, these restrictions exclude all participants interviewed between October 2021 and February 2022, rendering the length of data collection for Wave 10 (March 2022 – March 2023) similar to that observed for Waves 8 and 9. Furthermore, as during the pandemic, people aged 70 and older were often the main target of policy recommendations (Ayalon 2020; Perra 2021), we analysed whether health inequalities also differed by broad age groups (50–69 vs 70 and older). Finally, we also considered anxiety as a categorical variable with four levels of anxiety (very low: 0 to 1; low: 2 to 3; medium: 4 to 5; and high: 6 to 10) to capture a broader range of experiences of anxiety.

All computations were performed using Stata version 18 (Stata Corp, Union Station, Texas, USA).

### 2.6. Ethics approval

ELSA waves 8, 9, and 10 received ethical approval from the South Central – Berkshire Research Ethics Committee (15/SC/0526, 17/SC/0588, and 21/SC/0030).

### 2.7. Data availability statement

All data used in this study is available for download to *bona fide* researchers at the UK Data Service, http://doi.org/10.5255/UKDA-SN-5050-32 (Banks et al. 2024).

## 3. Results

Table 1 describes the cross-sectional samples at each of the three waves under study. As expected, the demographic and socioeconomic characteristics of the sample were relatively similar across waves, with 52–53 % being women, 23 % living alone, and about 46–48 % having no chronic conditions. However, the descriptive results for the different mental (ill-)health and well-being outcomes under study suggest, on average, a decline in the post-COVID Wave 10 compared to previous time points. For instance, the weighted prevalence of high depressive symptomatology ranged between 13.1–13.5 % in Waves 8 and 9 but was 16.6 % in Wave 10; the weighted prevalence of high loneliness remained relatively stable at 20.1 in 2016/17 and 20.8 % in 2018/19, but was 27.9 % in 2021/23. Similarly, the weighted prevalence of high anxiety was 16.3 % pre-COVID-19 and 17.9 % post-pandemic. The point estimate for the weighted average QoL score based on the CASP-19 also changed, ranging between 41.2–41.5 before the pandemic and dropping to 39.6 by the post-pandemic Wave 10.

The analytical models formally testing changes by wave, by each of the grouping variables (gender, living situation, and wealth quintiles), and the interaction between grouping variables and wave confirmed this overall decline in population mental health. The results of these models are shown in Table 2 (for the extended tables including the coefficients for the adjustment set, see Supplementary Table S1).

**Fig. 1.**
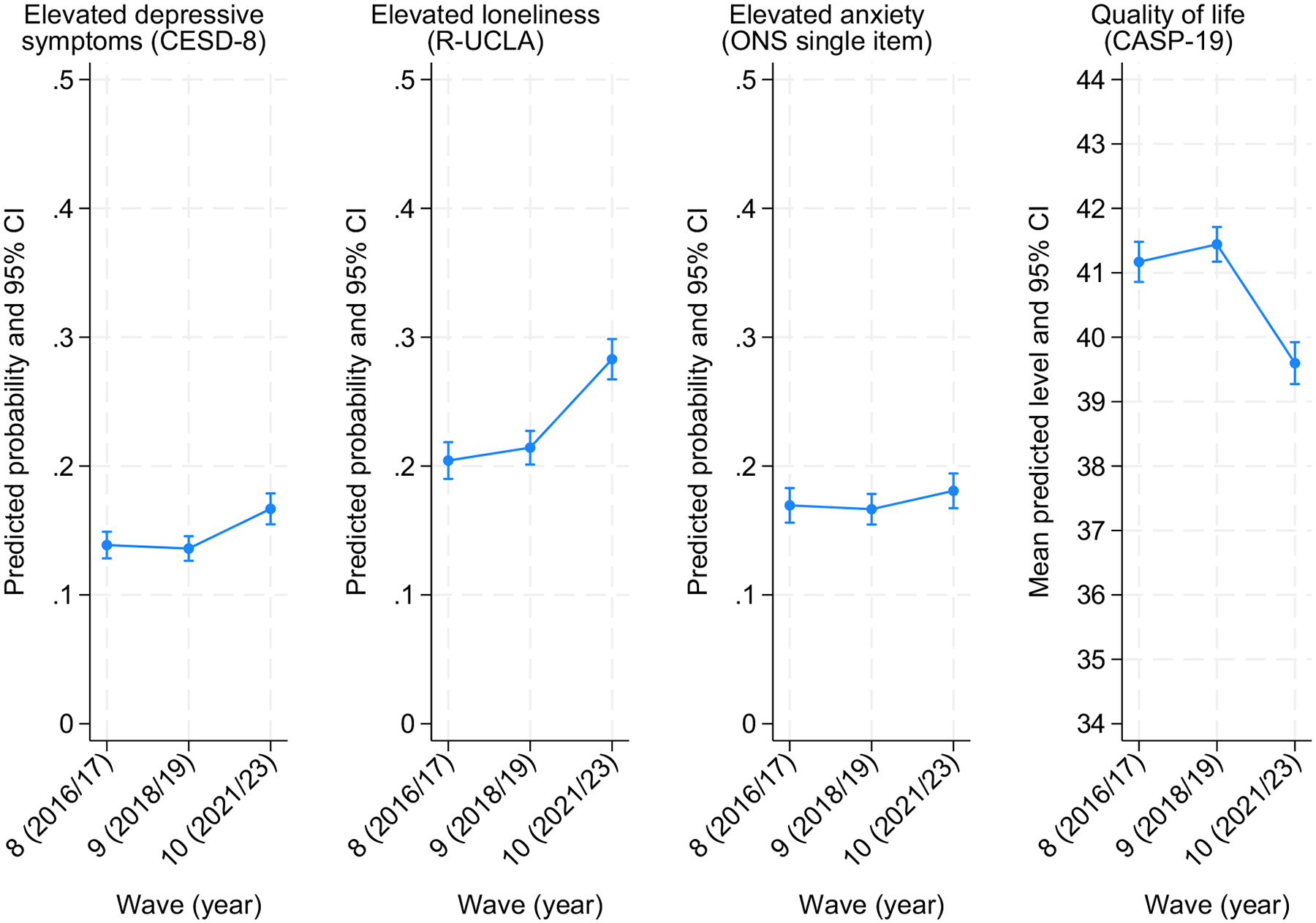
Marginal predicted probability of mental (ill-)health and predicted mean quality of life across time-points (brackets represent 95 % confidence intervals).

As shown by the overall models (Table 2, top section), there were no statistically significant changes in the prevalence of mental (ill-) health and the well-being levels between Waves 8 and 9. However, using Wave 9 as the reference time-point, the adjusted risk ratio (RR) for wave 10 was RR = 1.23 (95 % CI: 1.12, 1.34; *p* < 0.001) for high depressive symptomatology and RR = 1.32 (95 % CI: 1.22, 1.42; *p* < 0.001) for high loneliness. Similarly, between Waves 9 and 10, QoL levels declined by *B* = 1.84 points (95 % CI: −2.21, −1.48; *p* < 0.001). However, the difference in anxiety prevalence between Waves 9 and 10, observed in the weighted descriptive analyses, was not statistically significant, with RR = 1.02 (95 % CI: 0.98, 1.20; *p* = 0.105). Fig. 1 depicts the marginal predicted probabilities (or mean levels for QoL) of the different outcomes across the various waves.

**Fig. 2.**
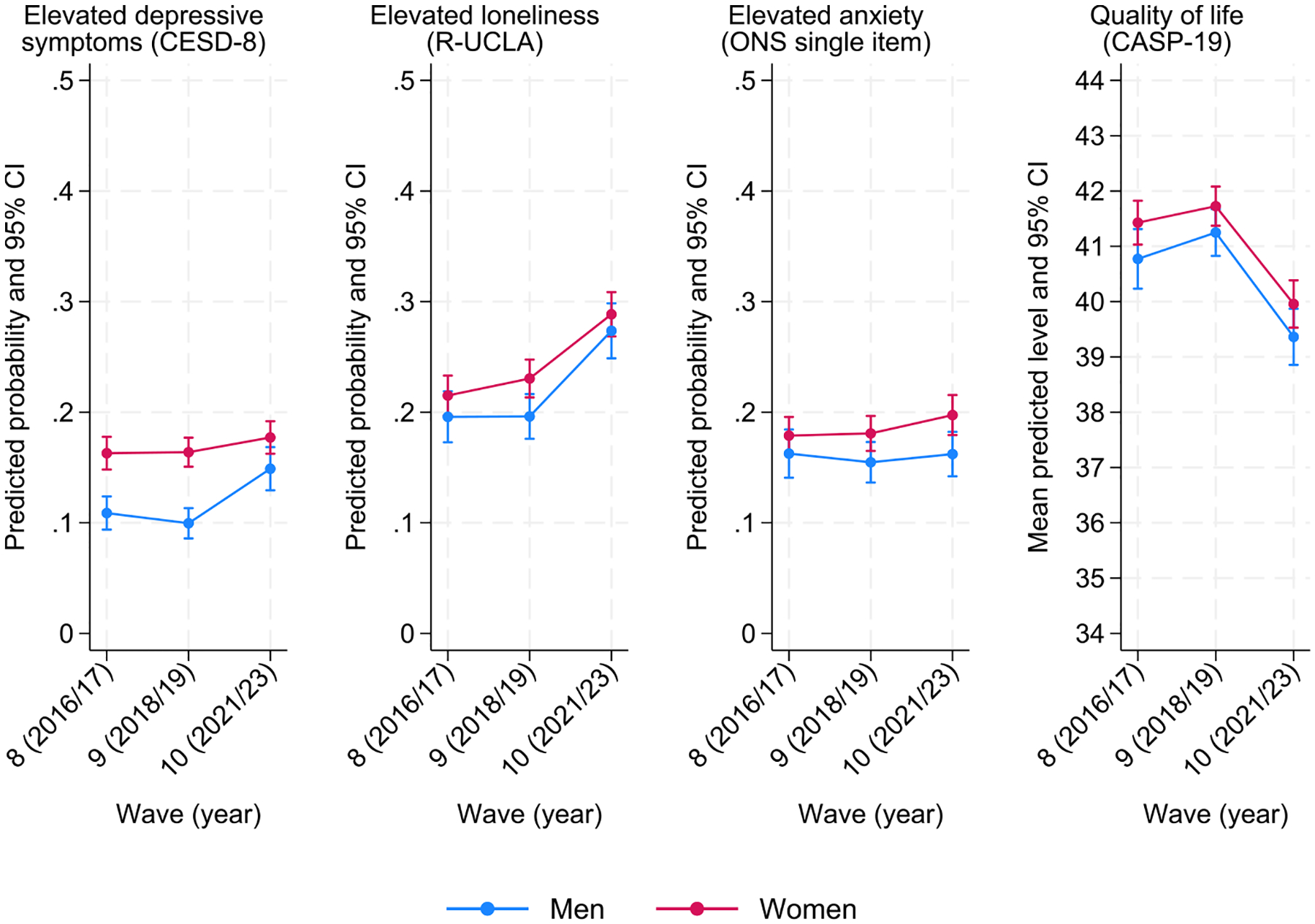
Marginal predicted probability of mental (ill-)health and predicted mean quality of life across time-points (brackets represent 95 % confidence intervals) by gender (women / men).

The models by groups (Table 2, bottom section) showed statistically significant gaps in mental (ill-)health and well-being by gender, living situation, and wealth and whether these have changed over the years. In line with the literature, we found that women were generally more likely to report poorer mental health. However, as shown in Fig. 2, most of these gaps have not changed across the years, with women consistently reporting, on average, poorer mental health and well-being. The only exception was observed for high depressive symptomatology, where we found a significant reduction in the gap between women and men (RR_women*wave10_ = 0.72, 95 % CI: 0.59, 0.89; *p* = 0.002). The visual inspection of the results shows that the reduction in the gap occurs as a result of men’s high depressive symptomatology probability increasing between Waves 9 and 10, rather than by a reduction in the probability of depression among women.

**Fig. 3.**
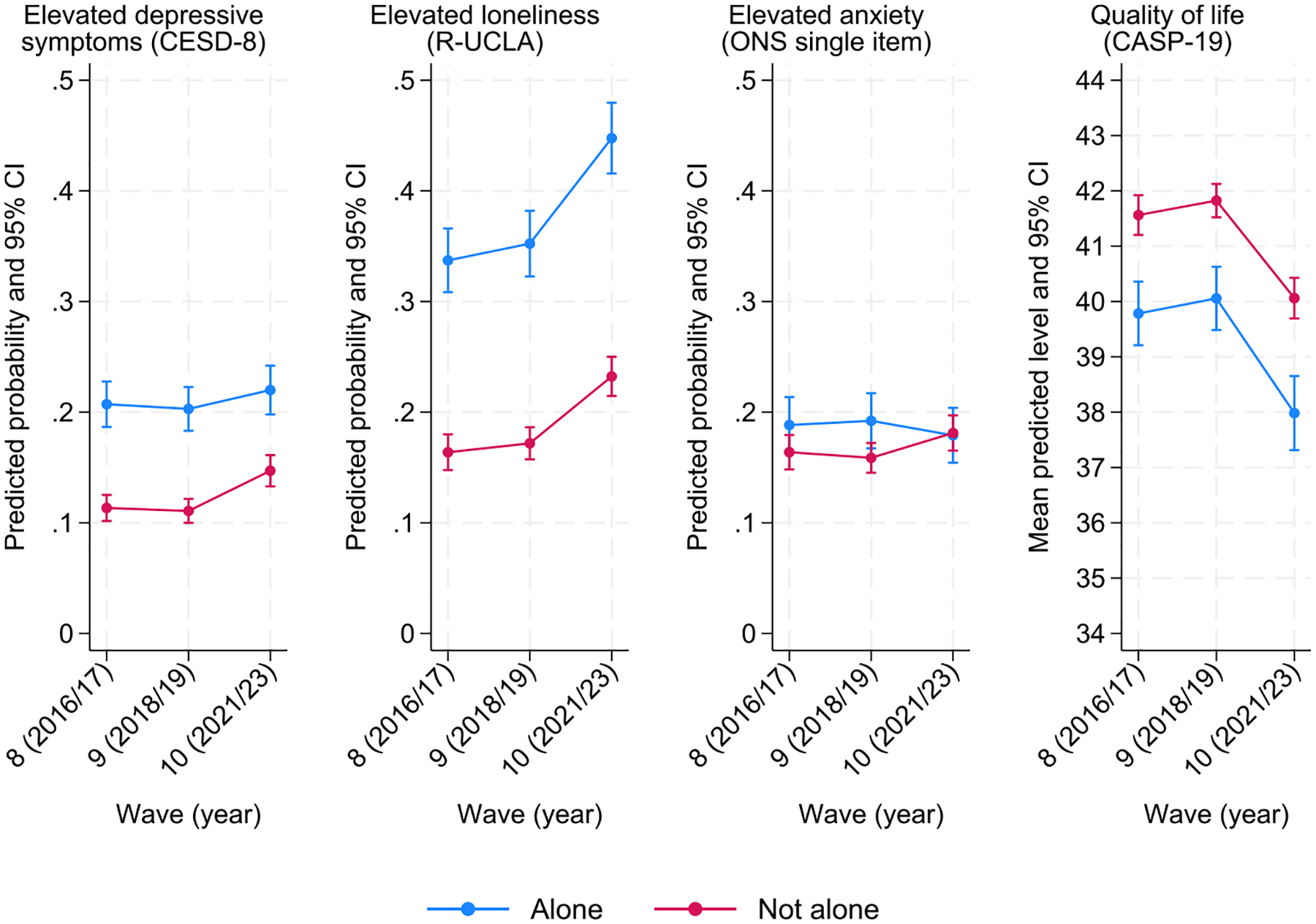
Marginal predicted probability of mental (ill-)health and predicted mean quality of life across time-points (brackets represent 95 % confidence intervals) by living situation (living alone / not living alone).

Fig. 3 visually shows the trends by living situation. For all outcomes examined, people who live alone consistently reported poorer mental health and well-being across all waves. The gaps remained there pre- and post-COVID-19 pandemic, except for a marginally significant larger increase in high depressive symptomatology between Waves 9 and 10 among those not living alone (RR_not_alone*wave10_ = 1.22, 95 % CI: 1.02, 1.47; *p* = 0.026).

**Fig. 4.**
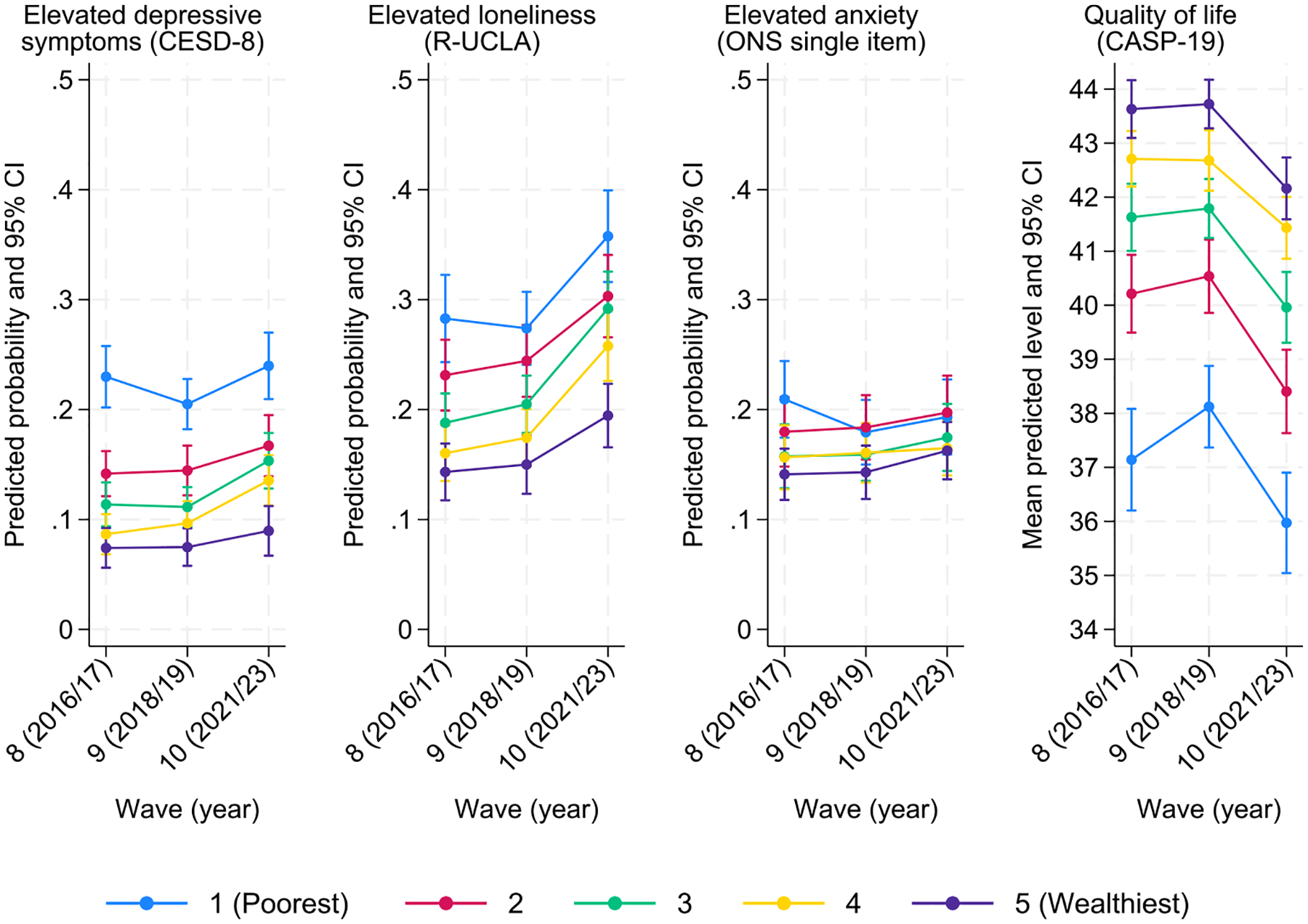
Marginal predicted probability of mental (ill-)health and predicted mean quality of life across time-points (brackets represent 95 % confidence intervals) by wealth quintiles.

Finally, Fig. 4 shows significant differences by wealth across all outcomes, with those in poorer wealth quintiles consistently reporting worse mental health and well-being than those in wealthier quintiles, with an apparent widening in the gaps for those in the poorest quintile. However, none of those effects significantly differed by wave, indicating that the gaps across wealth quintiles have remained broadly unchanged.

Results from the models using imputed data were very similar (Supplementary Table S2).

### 3.1. Robustness checks

Results from the models excluding respondents interviewed during the temporary restrictions between November 2021 and February 2022, using a non-face-to-face mode, or without a COVID-19 booster vaccine yielded remarkably similar results. Results from these models are included in Supplementary Table S3, and the visual depictions of the mental (ill-)health and well-being trends across the waves based on the results of these models are available in Supplementary Figures S1–S4. Results from the models by gender that also adjust for educational attainment also showed remarkably similar results (Supplementary Table S4).

Results from the multinomial models treating anxiety as an outcome with four categories were consistent with those from the simpler models treating anxiety as a dichotomous outcome. The results from these models are included in Supplementary Table S5, whereas the visual depiction of the predicted probability of each of the four categories at each time-point and group is provided in Supplementary Figures S5–S8.

In the models stratified by age group, the narrowing trend in the gender gap in depressive symptomatology was found to be statistically significant only for adults aged 70+ (RR_women*wave10_age70+_=0.60, 95 % CI: 0.46, 0.79; *p* < 0.001) but not for adults aged 50–69 (RR_women*wave10_age50–69_=0.79, 95 % CI: 0.60, 1.03; *p* = 0.079). Moreover, among adults aged 70+, quality of life levels were significantly lower in wave 8 when compared to wave 9 (B_wave8_age70+_=−0.53, 95 % CI: −0.88, −0.19; *p* = 0.003), although the difference was approximately three times the size when comparing waves 9 and 10 (B_wave10_age70+_=−1.65, 95 % CI: −2.07, −1.23; *p* < 0.001). Overall, the results from these models were largely consistent with those from the main analyses. Results from the models by age group are provided in Supplementary Tables S6–S7 (for ages 50–69 and 70+, respectively) and the corresponding visualisations are available in Supplementary Figures S9–S12.

## 4. Discussion

This paper investigated the extent to which mental (ill-)health post-COVID-19 pandemic (namely, depressive symptoms, anxiety, loneliness, and QoL) reversed back to levels observed before the outbreak and whether inequalities persisted in post-pandemic mental health. Our findings from nationally representative samples of older people living in England show that mental (ill-)health prevalence was pretty stable in the pre-pandemic years, with generally higher levels observed in the latest post-COVID-19 data collection. Unlike previous studies that focused on positive well-being and life satisfaction and suggested a “bounce back” to pre-pandemic levels when restrictions were lifted (Zacher and Rudolph 2024; Zaninotto et al. 2025), we found not only that mental (ill-)health is *not* “back to normal” (i.e., back to pre-pandemic levels), but also evidence suggesting a decline in mental (ill-)health and well-being. These different results could be due to different methodologies used, with our study involving repeat cross-sectional surveys with refreshment samples (in Waves 9 and 10) meant to provide an overall picture of societal changes, rather than a longitudinal assessment of change within the same individuals followed over time. However, our results on depression resonate with those of Zaninotto et al. (2025), who used within-individual methodology to identify trajectories of well-being. Therefore, such differences in findings are likely related to the outcome measures themselves, with positive psychological well-being and happiness relatively more stable over time than depression and quality of life (Eid and Diener 2004; Stegenga et al. 2012), with individuals returning to baseline levels of happiness even after significant (positive or negative) events (Kettlewell et al. 2020).

Importantly, this study also highlights that well-known health inequalities have broadly persisted post-pandemic, suggesting that the poorer mental health outcomes we observed at the population level post-COVID-19 were not driven by differential worsening in the specific subgroups we studied. We only observed a reduction in gaps for depression by gender and living arrangements; however, these gaps reduced not because of better levels amongst those historically known to be worse off (women and those living alone), but seemingly because of higher prevalence of depression among men and people who do not live alone. These could be driven by several factors, including differential effects of changes in networks and social relationships post-COVID-19 (Győri 2023), financial and economic circumstances (Louie et al. 2023), or help-seeking behaviours and resilience (Otten et al. 2021). Future studies are encouraged, using longitudinal data and relevant statistical methods that explore within-individual changes, to investigate factors that have contributed to increased depressive symptoms among older men and those not living alone in the aftermath of the pandemic.

### 4.1. Strengths and limitations

We investigated the prevalence at the population level of mental (ill-)health and well-being among people aged 50 and older in England before and after the COVID-19 pandemic. We used data from three waves of ELSA, a nationally representative study that has collected data on older adults in England since 2002. Using two pre-pandemic and one post-pandemic waves allowed us to formally test whether there were different levels of depressive symptomatology, anxiety, loneliness, and quality of life before and after COVID-19, or whether some trends were already observable pre-pandemic. Moreover, in our analyses, we reported weighted data that account for both different probabilities of being included in the sample and nonresponse to the survey. All variables used in this study were comparable (both in terms of outcomes and covariates, collected using the same questions and methodology), each wave was collected using similar study design, and robustness checks were carried out to account for differences in methods that occurred in Wave 10.

Our contribution, however, should be considered in light of some limitations. First, this study’s repeat cross-sectional design is appropriate for exploring health trends and documenting inequalities rather than establishing causality or investigating factors driving health differences. Second, although it safe to assume that COVID-19 is the driver behind the increased mental (ill-)health, we cannot rule out that other events (including the cost-of-living crisis in late 2021 and the Russian invasion of Ukraine in February 2022) might have contributed to the worse levels of mental health and well-being when ELSA Wave 10 took place. Third, given ELSA’s design, which samples only the over-50 population, we could not evaluate health trends across the whole adult age spectrum, although we were able to examine changes in mental health across broad age groups. Furthermore, we did not consider access to and availability of mental health services, even though these are crucial for positive outcomes and were particularly challenging during and soon after the COVID-19 pandemic. Finally, because of statistical power and lack of detailed information, our analyses did not consider specific groups (e.g., sexual and ethnic minority groups, but also older people living in institutions) likely disproportionately impacted by these declining trends.

In conclusion, our findings based on people aged 50 and above show an increase in the prevalence of depression, loneliness, and poorer quality of life among this age group following the COVID-19 lockdown and pandemic period. This highlights the need for public health initiatives aimed at addressing these trends. Policymakers should also continue to tackle health inequalities, which remained broadly similar to those observed before COVID-19, and to monitor groups that experienced a worsening in their depressive levels after COVID-19.

## Supplementary Material

Supplementary Material

Supplementary material associated with this article can be found, in the online version, at doi:10.1016/j.jadr.2025.101012.

## Figures and Tables

**Table 1:** Descriptive results of the cross-sectional samples at each wave, weighted results.

	Wave 8 (2016–2017)	Wave 9 (2018–2019)	Wave 10 (2021–2023)
Main survey N	6889	6940	6041
Self-completion N	6257	6359	5440
**Outcomes**			
High depressive symptomatology (CESD-8 ≥ 4)			
No	85.47 %	85.96 %	81.08 %
Yes	13.51 %	13.14 %	16.61 %
*Missing*	*1.01 %*	*0.90 %*	*2.30 %*
High anxiety (ONS ≥ 6)*			
No	79.73 %	80.92 %	79.88 %
Very low (0–1) **	39.12 %	45.43 %	42.94 %
Low (2–3) **	25.18 %	22.85 %	22.78 %
Medium (4–5) **	15.43 %	12.64 %	14.15 %
Yes	16.34 %	16.32 %	17.94 %
*Missing*	*3.93 %*	*2.76 %*	*2.19 %*
High loneliness (UCLA-3 ≥ 6)*			
No	77.80 %	76.56 %	69.98 %
Yes	20.09 %	20.76 %	27.87 %
*Missing*	*2.11 %*	*2.68 %*	*2.16 %*
Quality of life (CASP-19), M (95 % CI)*	41.16 (40.81, 41.52)	41.52 (41.22, 41.83)	39.57 (39.21, 39.94)
*Missing*	*7.02 %*	*7.45 %*	*6.03 %*
**Groups**			
Gender (% women)	52.53 %	52.86 %	52.84 %
Living situation (% not living alone)	76.64 %	76.78 %	76.85 %
Wealth quintiles			
1st (poorest)	19.78 %	19.86 %	20.51 %
2nd	19.72 %	19.68 %	18.05 %
3rd	19.64 %	19.93 %	18.95 %
4th	19.84 %	19.48 %	19.02 %
5th (wealthiest)	19.66 %	19.43 %	19.03 %
*Missing*	*1.35 %*	*1.61 %*	*4.45 %*
**Covariates**			
Age group			
50–59	31.51 %	35.32 %	35.98 %
60–69	32.81 %	29.32 %	29.04 %
70–79	22.72 %	22.21 %	22.86 %
80+	12.96 %	13.15 %	12.12 %
Highest educational qualification			
High (University or above)	18.02 %	19.87 %	23.21 %
Middle (Upper secondary education)	55.91 %	59.34 %	59.41 %
Low (Below lower secondary education)	21.34 %	18.13 %	15.56 %
*Missing*	*4.72 %*	*2.66 %*	*1.81 %*
Chronic conditions (% living with long-standing illness)			
No illness	46.47 %	47.58 %	46.29 %
Non-limiting illness	20.35 %	20.27 %	19.35 %
Limiting illness	33.16 %	32.07 %	34.32 %
*Missing*	*0.02 %*	*0.08 %*	*0.04 %*
**Sensitivity check variables**			
Received COVID-19 vaccination boost			
No			15.48 %
Yes			84.44 %
*Missing*			*0.08 %*
Interviewed during period of reinstated restrictions (Oct 2021 - Jan 2022) (%)			8.31 %
Not interviewed face-to-face (%)			19.92 %

*Note*. Weighted data.

* Variables weighted with self-completion weights to account for the differential likelihood of completing the self-completion questionnaire.

** The ‘No high anxiety’ category used in the main analyses comprises the ‘Very low’, ‘Low’, and ‘Medium’ categories; these subcategories are used in supplementary analyses treating this outcome as multi-categorical.

CASP-19: 19-item Control, Autonomy, Self-realisation and Pleasure; CESD-8: 8-item Centre for Epidemiologic Studies Depression Scale; CI: confidence interval; M: mean; ONS: Office for National Statistics; UCLA-3: University of California Los Angeles 3-item loneliness scale.

**Table 2:** Results from the modified Poisson and linear regression models testing the statistical effect of time (wave), the grouping variables, and their interaction. Weighted results.

		High depressive symptomatology (CESD-8 ≥ 4)				High anxiety (ONS single question ≥ 6)				High loneliness (UCLA-3 ≥ 6)				Quality of life (CASP-19)			
	Variable	RR	LB	UB	p-value	RR	LB	UB	p-value	RR	LB	UB	p-value	B	LB	UB	p-value
**Overall**	Wave (ref. Wave 9)				<0.001				0.253				<0.001				<0.001
	Wave 8	1.02	0.93	1.11	0.660	1.02	0.92	1.12	0.719	0.95	0.88	1.03	0.209	−0.27	−0.59	0.05	0.093
	Wave 10	1.23	1.12	1.34	<0.001	1.09	0.98	1.20	0.105	1.32	1.22	1.42	<0.001	−1.84	−2.21	−1.48	<0.001
**By gender**	Gender: Women	1.65	1.40	1.93	<0.001	1.17	1.01	1.35	0.038	1.17	1.03	1.33	0.013	0.48	−0.08	1.03	0.091
	Wave (ref. Wave 9)				<0.001				0.785				<0.001				<0.001
	Wave 8	1.09	0.93	1.29	0.292	1.05	0.90	1.23	0.541	1.00	0.88	1.13	0.977	−0.48	−1.01	0.06	0.079
	Wave 10	1.50	1.26	1.78	<0.001	1.05	0.89	1.24	0.587	1.39	1.23	1.59	<0.001	−1.89	−2.44	−1.33	<0.001
	Wave (ref. Wave 9) * Gender (ref. Men)				0.007				0.651				0.373				0.853
	Wave 8 * Women	0.91	0.75	1.10	0.340	0.94	0.77	1.14	0.544	0.94	0.80	1.09	0.386	0.18	−0.48	0.84	0.594
	Wave 10 * Women	0.72	0.59	0.89	0.002	1.04	0.85	1.28	0.696	0.90	0.77	1.05	0.177	0.12	−0.62	0.85	0.755
**By living situation**	Living situation: Not living alone	0.55	0.48	0.63	<0.001	0.83	0.71	0.96	0.015	0.49	0.43	0.55	<0.001	1.77	1.12	2.41	<0.001
	Wave (ref. Wave 9)				0.432				0.734				<0.001				<0.001
	Wave 8	1.02	0.91	1.15	0.727	0.98	0.83	1.16	0.813	0.96	0.87	1.05	0.357	−0.27	−0.92	0.37	0.406
	Wave 10	1.08	0.96	1.23	0.204	0.93	0.78	1.11	0.439	1.27	1.15	1.40	<0.001	−2.07	−2.85	−1.30	<0.001
	Wave (ref. Wave 9) * Living situation (ref. Living alone)				0.050				0.170				0.635				0.769
	Wave 8 * Not living alone	1.00	0.85	1.19	0.972	1.05	0.86	1.29	0.618	1.00	0.86	1.15	0.956	0.01	−0.74	0.76	0.977
	Wave 10 * Not living alone	1.22	1.02	1.47	0.026	1.23	0.99	1.52	0.065	1.06	0.92	1.23	0.398	0.31	−0.57	1.19	0.492
**By wealth quintile**	Wealth quintile (ref. 1st, Poorest)				<0.001				0.233				<0.001				<0.001
	2nd	0.71	0.58	0.86	<0.001	1.02	0.81	1.29	0.834	0.89	0.74	1.07	0.215	2.41	1.38	3.45	<0.001
	3rd	0.54	0.45	0.66	<0.001	0.89	0.71	1.11	0.289	0.75	0.63	0.89	0.001	3.67	2.73	4.61	<0.001
	4th	0.47	0.37	0.60	<0.001	0.90	0.70	1.14	0.370	0.64	0.52	0.77	<0.001	4.56	3.60	5.51	<0.001
	5th, Wealthiest	0.37	0.28	0.47	<0.001	0.80	0.63	1.01	0.065	0.55	0.44	0.68	<0.001	5.60	4.71	6.49	<0.001
	Wave (ref. Wave 9)				0.104				0.350				0.002				<0.001
	Wave 8	1.12	0.97	1.30	0.125	1.17	0.95	1.44	0.147	1.03	0.88	1.21	0.696	−0.98	−2.04	0.08	0.069
	Wave 10	1.17	1.00	1.37	0.049	1.08	0.85	1.36	0.528	1.31	1.12	1.53	0.001	−2.15	−3.27	−1.03	<0.001
	Wave (ref. Wave 9) * Wealth quintile (ref. 1st, Poorest)				0.365				0.930				0.653				0.748
	Wave 8 * 2nd	0.87	0.68	1.13	0.298	0.84	0.62	1.13	0.248	0.92	0.71	1.18	0.507	0.66	−0.82	2.13	0.383
	Wave 8 * 3rd	0.91	0.70	1.19	0.490	0.85	0.62	1.16	0.309	0.89	0.70	1.13	0.333	0.82	−0.49	2.12	0.219
	Wave 8 * 4th	0.80	0.58	1.11	0.178	0.84	0.61	1.15	0.273	0.89	0.69	1.14	0.360	1.01	−0.23	2.25	0.111
	Wave 8 * 5th, Wealthiest	0.88	0.63	1.25	0.479	0.85	0.63	1.14	0.275	0.93	0.71	1.21	0.571	0.89	−0.32	2.09	0.148
	Wave 10 * 2nd	0.99	0.75	1.30	0.932	1.00	0.72	1.38	0.981	0.95	0.75	1.21	0.677	0.02	−1.52	1.55	0.982
	Wave 10 * 3rd	1.18	0.90	1.55	0.237	1.02	0.74	1.41	0.906	1.09	0.87	1.37	0.452	0.32	−1.07	1.71	0.654
	Wave 10 * 4th	1.20	0.88	1.64	0.248	0.95	0.69	1.31	0.769	1.13	0.89	1.44	0.311	0.90	−0.49	2.29	0.203
	Wave 10 * 5th, Wealthiest	1.02	0.72	1.46	0.898	1.06	0.77	1.46	0.742	0.99	0.77	1.29	0.959	0.59	−0.71	1.89	0.376

Note. Weighted and adjusted models (adjusted for age group, gender, educational attainment, and chronic conditions; models by gender do not include educational attainment to avoid over-adjustment). B: regression coefficient; CASP-19: 19-item Control, Autonomy, Self-realisation and Pleasure; CESD-8: 8-item Centre for Epidemiologic Studies Depression Scale; LB: 95 % confidence interval lower bound; ONS: Office for National Statistics; RR: risk ratio; UB: 95 % confidence interval upper bound; UCLA-3: University of California Los Angeles 3-item loneliness scale. P-values for overall wave, wealth, and the interaction between wave and the grouping variable correspond to the omnibus test.
