# Supplementary material for: Mental health in adults aged 50+ since the COVID-19 pandemic: Are we (all) back to ‘normal’? evidence from England
Source: J Affect Disord Rep. Author manuscript; Available in PMC 2026 Jun 8. (PMC13008333; doi:10.1016/j.jadr.2025.101012)

**Mental Health in Adults Aged 50+ Since the COVID-19 Pandemic: Are We (All) Back to ‘Normal’? Evidence From England**

**Supplementary material**

[Table S1. Extended results from the modified Poisson and linear regression models testing the statistical effect of time (wave), the grouping variables, and their interaction, weighted results. 3](#_Toc214549602)

[Table S2. Extended results from the modified Poisson and linear regression models testing the statistical effect of time (wave), the grouping variables, and their interaction, weighted results, using imputed data. 7](#_Toc214549603)

[Table S3. Results from the modified Poisson and linear regression models excluding participants interviewed during the temporary restrictions between November 2021 and February 2022, using a non-face-to-face mode, or without a COVID-19 booster vaccine. Weighted results. 11](#_Toc214549604)

[Table S4. Results from the models by gender including educational attainment as part of the adjustment set. Weighted results. 15](#_Toc214549605)

[Table S5. Results from multinomial models with anxiety as a multi-categorical outcome. Weighted results. 16](#_Toc214549606)

[Table S6. Results from the models stratified by age group. Weighted results. Age group: 50 to 69 year-olds. 19](#_Toc214549607)

[Table S7. Results from the models stratified by age group. Weighted results. Age group: 70+ year-olds. 22](#_Toc214549608)

[Figure S1. Marginal predicted probability of mental (ill-)health and predicted mean quality of life across time-points (brackets represent 95% confidence intervals) after excluding participants interviewed during the temporary restrictions between November 2021 and February 2022, using a non-face-to-face mode, or without a COVID-19 booster vaccine. 25](#_Toc214549609)

[Figure S2. Marginal predicted probability of mental (ill-)health and predicted mean quality of life across time-points (brackets represent 95% confidence intervals) by gender (women / men) after excluding participants interviewed during the temporary restrictions between November 2021 and February 2022, using a non-face-to-face mode, or without a COVID-19 booster vaccine. 26](#_Toc214549610)

[Figure S3. Marginal predicted probability of mental (ill-)health and predicted mean quality of life across time-points (brackets represent 95% confidence intervals) by living situation (living alone / not living alone) after excluding participants interviewed during the temporary restrictions between November 2021 and February 2022, using a non-face-to-face mode, or without a COVID-19 booster vaccine. 28](#_Toc214549611)

[Figure S4. Marginal predicted probability of mental (ill-)health and predicted mean quality of life across time-points (brackets represent 95% confidence intervals) by wealth quintiles after excluding participants interviewed during the temporary restrictions between November 2021 and February 2022, using a non-face-to-face mode, or without a COVID-19 booster vaccine. 30](#_Toc214549612)

[Figure S5. Marginal predicted probability of four different anxiety categories (measured with the UK Office for National Statistics single question) across time-points (brackets represent 95% confidence intervals) from multinomial models. 31](#_Toc214549613)

[Figure S6. Marginal predicted probability of four different anxiety categories (measured with the UK Office for National Statistics single question) across time-points (brackets represent 95% confidence intervals) from multinomial models. Results by gender (women/men). 32](#_Toc214549614)

[Figure S7. Marginal predicted probability of four different anxiety categories (measured with the UK Office for National Statistics single question) across time-points (brackets represent 95% confidence intervals) from multinomial models. Results by living situation (living alone / not living alone) 33](#_Toc214549615)

[Figure S8. Marginal predicted probability of four different anxiety categories (measured with the UK Office for National Statistics single question) across time-points (brackets represent 95% confidence intervals) from multinomial models. Results by wealth quintiles. Wave 8: 2016-2017; wave 9: 2018-2019; wave 10: 2021-2023. 34](#_Toc214549616)

[Figure S9. Marginal predicted probability of mental (ill-)health and predicted mean quality of life across time-points (brackets represent 95% confidence intervals). Results by age groups (50-69 and 70+ year-olds). 35](#_Toc214549617)

[Figure S10. Marginal predicted probability of mental (ill-)health and predicted mean quality of life across time-points (brackets represent 95% confidence intervals). Results by age groups (50-69 and 70+ year-olds) and gender (women / men). 36](#_Toc214549618)

[Figure S11. Marginal predicted probability of mental (ill-)health and predicted mean quality of life across time-points (brackets represent 95% confidence intervals). Results by age groups (50-69 and 70+ year-olds) and living situation (living alone / not living alone). 37](#_Toc214549619)

[Figure S12. Marginal predicted probability of mental (ill-)health and predicted mean quality of life across time-points (brackets represent 95% confidence intervals). Results by age groups (50-69 and 70+ year-olds) and wealth quintiles. 38](#_Toc214549620)

# Table S1. Extended results from the modified Poisson and linear regression models testing the statistical effect of time (wave), the grouping variables, and their interaction, weighted results.

|  |  | High depressive symptomatology  (CESD-8 ≥ 4) | | | |  | High anxiety  (ONS single question ≥ 6) | | | |  | High loneliness  (UCLA-3 ≥ 6) | | | |  | Quality of life  (CASP-19) | | | |
| --- | --- | --- | --- | --- | --- | --- | --- | --- | --- | --- | --- | --- | --- | --- | --- | --- | --- | --- | --- | --- |
|  | Variable | RR | LB | UB | p-value |  | RR | LB | UB | p-value |  | RR | LB | UB | p-value |  | B | LB | UB | p-value |
| Overall | Wave (ref. Wave 9) |  |  |  | <0.001 |  |  |  |  | 0.253 |  |  |  |  | <0.001 |  |  |  |  | <0.001 |
|  | Wave 8 | 1.02 | 0.93 | 1.11 | 0.660 |  | 1.02 | 0.92 | 1.12 | 0.719 |  | 0.95 | 0.88 | 1.03 | 0.209 |  | -0.27 | -0.59 | 0.05 | 0.093 |
|  | Wave 10 | 1.23 | 1.12 | 1.34 | <0.001 |  | 1.09 | 0.98 | 1.20 | 0.105 |  | 1.32 | 1.22 | 1.42 | <0.001 |  | -1.84 | -2.21 | -1.48 | <0.001 |
|  | Age group (ref. 50-59) |  |  |  |  |  |  |  |  |  |  |  |  |  |  |  |  |  |  |  |
|  | 60-69 | 0.71 | 0.63 | 0.79 | <0.001 |  | 0.84 | 0.74 | 0.94 | 0.003 |  | 0.84 | 0.76 | 0.94 | 0.001 |  | 2.00 | 1.44 | 2.55 | <0.001 |
|  | 70-79 | 0.53 | 0.47 | 0.60 | <0.001 |  | 0.71 | 0.63 | 0.81 | <0.001 |  | 0.68 | 0.61 | 0.77 | <0.001 |  | 2.16 | 1.57 | 2.74 | <0.001 |
|  | 80+ | 0.63 | 0.55 | 0.72 | <0.001 |  | 0.64 | 0.55 | 0.75 | <0.001 |  | 0.78 | 0.69 | 0.89 | <0.001 |  | 0.45 | -0.24 | 1.15 | 0.204 |
|  | Gender: Women | 1.37 | 1.23 | 1.53 | <0.001 |  | 1.19 | 1.08 | 1.31 | 0.001 |  | 1.11 | 1.02 | 1.21 | 0.020 |  | 0.65 | 0.21 | 1.09 | 0.004 |
|  | Highest educational qualification (ref. Post-secondary) |  |  |  |  |  |  |  |  |  |  |  |  |  |  |  |  |  |  |  |
|  | A-level, O-Level, CSE, technical equivalent | 1.41 | 1.22 | 1.63 | <0.001 |  | 0.86 | 0.76 | 0.96 | 0.010 |  | 1.32 | 1.17 | 1.50 | <0.001 |  | -1.81 | -2.30 | -1.33 | <0.001 |
|  | No academic qualification | 1.89 | 1.61 | 2.22 | <0.001 |  | 1.09 | 0.94 | 1.27 | 0.265 |  | 1.59 | 1.38 | 1.84 | <0.001 |  | -3.50 | -4.19 | -2.81 | <0.001 |
|  | Chronic conditions (ref. No illness) |  |  |  |  |  |  |  |  |  |  |  |  |  |  |  |  |  |  |  |
|  | Non-limiting illness | 1.50 | 1.28 | 1.77 | <0.001 |  | 1.11 | 0.98 | 1.26 | 0.107 |  | 1.26 | 1.13 | 1.41 | <0.001 |  | -1.83 | -2.30 | -1.37 | <0.001 |
|  | Limiting illness | 4.46 | 3.92 | 5.07 | <0.001 |  | 1.67 | 1.50 | 1.86 | <0.001 |  | 2.00 | 1.83 | 2.20 | <0.001 |  | -8.50 | -9.00 | -8.01 | <0.001 |
| By gender | Gender: Women | 1.65 | 1.40 | 1.93 | <0.001 |  | 1.17 | 1.01 | 1.35 | 0.038 |  | 1.17 | 1.03 | 1.33 | 0.013 |  | 0.48 | -0.08 | 1.03 | 0.091 |
|  | Wave (ref. Wave 9) |  |  |  | <0.001 |  |  |  |  | 0.785 |  |  |  |  | <0.001 |  |  |  |  | <0.001 |
|  | Wave 8 | 1.09 | 0.93 | 1.29 | 0.292 |  | 1.05 | 0.90 | 1.23 | 0.541 |  | 1.00 | 0.88 | 1.13 | 0.977 |  | -0.48 | -1.01 | 0.06 | 0.079 |
|  | Wave 10 | 1.50 | 1.26 | 1.78 | <0.001 |  | 1.05 | 0.89 | 1.24 | 0.587 |  | 1.39 | 1.23 | 1.59 | <0.001 |  | -1.89 | -2.44 | -1.33 | <0.001 |
|  | Wave (ref. Wave 9) * Gender (ref. Men) |  |  |  | 0.007 |  |  |  |  | 0.651 |  |  |  |  | 0.373 |  |  |  |  | 0.853 |
|  | Wave 8 * Women | 0.91 | 0.75 | 1.10 | 0.340 |  | 0.94 | 0.77 | 1.14 | 0.544 |  | 0.94 | 0.80 | 1.09 | 0.386 |  | 0.18 | -0.48 | 0.84 | 0.594 |
|  | Wave 10 * Women | 0.72 | 0.59 | 0.89 | 0.002 |  | 1.04 | 0.85 | 1.28 | 0.696 |  | 0.90 | 0.77 | 1.05 | 0.177 |  | 0.12 | -0.62 | 0.85 | 0.755 |
|  | Age group (ref. 50-59) |  |  |  |  |  |  |  |  |  |  |  |  |  |  |  |  |  |  |  |
|  | 60-69 | 0.73 | 0.65 | 0.81 | <0.001 |  | 0.84 | 0.75 | 0.94 | 0.003 |  | 0.86 | 0.77 | 0.95 | 0.004 |  | 1.80 | 1.23 | 2.38 | <0.001 |
|  | 70-79 | 0.57 | 0.51 | 0.65 | <0.001 |  | 0.72 | 0.64 | 0.81 | <0.001 |  | 0.71 | 0.64 | 0.80 | <0.001 |  | 1.75 | 1.16 | 2.35 | <0.001 |
|  | 80+ | 0.71 | 0.62 | 0.81 | <0.001 |  | 0.67 | 0.58 | 0.78 | <0.001 |  | 0.85 | 0.75 | 0.96 | 0.010 |  | -0.30 | -0.99 | 0.40 | 0.403 |
|  | Chronic conditions (ref. No illness) |  |  |  |  |  |  |  |  |  |  |  |  |  |  |  |  |  |  |  |
|  | Non-limiting illness | 1.57 | 1.33 | 1.86 | <0.001 |  | 1.15 | 1.01 | 1.32 | 0.041 |  | 1.26 | 1.13 | 1.42 | <0.001 |  | -1.87 | -2.41 | -1.32 | <0.001 |
|  | Limiting illness | 4.81 | 4.23 | 5.46 | <0.001 |  | 1.68 | 1.52 | 1.87 | <0.001 |  | 2.06 | 1.88 | 2.26 | <0.001 |  | -8.67 | -9.18 | -8.17 | <0.001 |
| By living situation | Living situation: Not living alone | 0.55 | 0.48 | 0.63 | <0.001 |  | 0.83 | 0.71 | 0.96 | 0.015 |  | 0.49 | 0.43 | 0.55 | <0.001 |  | 1.77 | 1.12 | 2.41 | <0.001 |
|  | Wave (ref. Wave 9) |  |  |  | 0.432 |  |  |  |  | 0.734 |  |  |  |  | <0.001 |  |  |  |  | <0.001 |
|  | Wave 8 | 1.02 | 0.91 | 1.15 | 0.727 |  | 0.98 | 0.83 | 1.16 | 0.813 |  | 0.96 | 0.87 | 1.05 | 0.357 |  | -0.27 | -0.92 | 0.37 | 0.406 |
|  | Wave 10 | 1.08 | 0.96 | 1.23 | 0.204 |  | 0.93 | 0.78 | 1.11 | 0.439 |  | 1.27 | 1.15 | 1.40 | <0.001 |  | -2.07 | -2.85 | -1.30 | <0.001 |
|  | Wave (ref. Wave 9) * Living situation (ref. Living alone) |  |  |  | 0.050 |  |  |  |  | 0.170 |  |  |  |  | 0.635 |  |  |  |  | 0.769 |
|  | Wave 8 * Not living alone | 1.00 | 0.85 | 1.19 | 0.972 |  | 1.05 | 0.86 | 1.29 | 0.618 |  | 1.00 | 0.86 | 1.15 | 0.956 |  | 0.01 | -0.74 | 0.76 | 0.977 |
|  | Wave 10 * Not living alone | 1.22 | 1.02 | 1.47 | 0.026 |  | 1.23 | 0.99 | 1.52 | 0.065 |  | 1.06 | 0.92 | 1.23 | 0.398 |  | 0.31 | -0.57 | 1.19 | 0.492 |
|  | Age group (ref. 50-59) |  |  |  |  |  |  |  |  |  |  |  |  |  |  |  |  |  |  |  |
|  | 60-69 | 0.70 | 0.62 | 0.78 | <0.001 |  | 0.83 | 0.74 | 0.94 | 0.003 |  | 0.82 | 0.74 | 0.91 | <0.001 |  | 2.07 | 1.52 | 2.62 | <0.001 |
|  | 70-79 | 0.51 | 0.45 | 0.58 | <0.001 |  | 0.71 | 0.62 | 0.80 | <0.001 |  | 0.65 | 0.58 | 0.72 | <0.001 |  | 2.31 | 1.73 | 2.89 | <0.001 |
|  | 80+ | 0.54 | 0.47 | 0.61 | <0.001 |  | 0.62 | 0.53 | 0.73 | <0.001 |  | 0.64 | 0.56 | 0.72 | <0.001 |  | 0.96 | 0.26 | 1.66 | 0.007 |
|  | Gender: Women | 1.35 | 1.21 | 1.50 | <0.001 |  | 1.18 | 1.07 | 1.30 | 0.001 |  | 1.07 | 0.99 | 1.17 | 0.104 |  | 0.75 | 0.32 | 1.19 | 0.001 |
|  | Highest educational qualification (ref. Post-secondary) |  |  |  |  |  |  |  |  |  |  |  |  |  |  |  |  |  |  |  |
|  | A-level, O-Level, CSE, technical equivalent | 1.40 | 1.21 | 1.61 | <0.001 |  | 0.86 | 0.76 | 0.96 | 0.009 |  | 1.31 | 1.16 | 1.47 | <0.001 |  | -1.79 | -2.27 | -1.30 | <0.001 |
|  | No academic qualification | 1.81 | 1.54 | 2.12 | <0.001 |  | 1.09 | 0.93 | 1.26 | 0.287 |  | 1.53 | 1.32 | 1.76 | <0.001 |  | -3.42 | -4.11 | -2.73 | <0.001 |
|  | Chronic conditions (ref. No illness) |  |  |  |  |  |  |  |  |  |  |  |  |  |  |  |  |  |  |  |
|  | Non-limiting illness | 1.48 | 1.25 | 1.74 | <0.001 |  | 1.11 | 0.98 | 1.26 | 0.114 |  | 1.24 | 1.11 | 1.38 | <0.001 |  | -1.80 | -2.26 | -1.33 | <0.001 |
|  | Limiting illness | 4.18 | 3.67 | 4.76 | <0.001 |  | 1.65 | 1.49 | 1.84 | <0.001 |  | 1.85 | 1.69 | 2.03 | <0.001 |  | -8.36 | -8.85 | -7.87 | <0.001 |
| By wealth quintile | Wealth quintile (ref. 1st, Poorest) |  |  |  | <0.001 |  |  |  |  | 0.233 |  |  |  |  | <0.001 |  |  |  |  | <0.001 |
|  | 2nd | 0.71 | 0.58 | 0.86 | <0.001 |  | 1.02 | 0.81 | 1.29 | 0.834 |  | 0.89 | 0.74 | 1.07 | 0.215 |  | 2.41 | 1.38 | 3.45 | <0.001 |
|  | 3rd | 0.54 | 0.45 | 0.66 | <0.001 |  | 0.89 | 0.71 | 1.11 | 0.289 |  | 0.75 | 0.63 | 0.89 | 0.001 |  | 3.67 | 2.73 | 4.61 | <0.001 |
|  | 4th | 0.47 | 0.37 | 0.60 | <0.001 |  | 0.90 | 0.70 | 1.14 | 0.370 |  | 0.64 | 0.52 | 0.77 | <0.001 |  | 4.56 | 3.60 | 5.51 | <0.001 |
|  | 5th, Wealthiest | 0.37 | 0.28 | 0.47 | <0.001 |  | 0.80 | 0.63 | 1.01 | 0.065 |  | 0.55 | 0.44 | 0.68 | <0.001 |  | 5.60 | 4.71 | 6.49 | <0.001 |
|  | Wave (ref. Wave 9) |  |  |  | 0.104 |  |  |  |  | 0.350 |  |  |  |  | 0.002 |  |  |  |  | <0.001 |
|  | Wave 8 | 1.12 | 0.97 | 1.30 | 0.125 |  | 1.17 | 0.95 | 1.44 | 0.147 |  | 1.03 | 0.88 | 1.21 | 0.696 |  | -0.98 | -2.04 | 0.08 | 0.069 |
|  | Wave 10 | 1.17 | 1.00 | 1.37 | 0.049 |  | 1.08 | 0.85 | 1.36 | 0.528 |  | 1.31 | 1.12 | 1.53 | 0.001 |  | -2.15 | -3.27 | -1.03 | <0.001 |
|  | Wave (ref. Wave 9) * Wealth quintile (ref. 1st, Poorest) |  |  |  | 0.365 |  |  |  |  | 0.930 |  |  |  |  | 0.653 |  |  |  |  | 0.748 |
|  | Wave 8 * 2nd | 0.87 | 0.68 | 1.13 | 0.298 |  | 0.84 | 0.62 | 1.13 | 0.248 |  | 0.92 | 0.71 | 1.18 | 0.507 |  | 0.66 | -0.82 | 2.13 | 0.383 |
|  | Wave 8 * 3rd | 0.91 | 0.70 | 1.19 | 0.490 |  | 0.85 | 0.62 | 1.16 | 0.309 |  | 0.89 | 0.70 | 1.13 | 0.333 |  | 0.82 | -0.49 | 2.12 | 0.219 |
|  | Wave 8 * 4th | 0.80 | 0.58 | 1.11 | 0.178 |  | 0.84 | 0.61 | 1.15 | 0.273 |  | 0.89 | 0.69 | 1.14 | 0.360 |  | 1.01 | -0.23 | 2.25 | 0.111 |
|  | Wave 8 * 5th, Wealthiest | 0.88 | 0.63 | 1.25 | 0.479 |  | 0.85 | 0.63 | 1.14 | 0.275 |  | 0.93 | 0.71 | 1.21 | 0.571 |  | 0.89 | -0.32 | 2.09 | 0.148 |
|  | Wave 10 * 2nd | 0.99 | 0.75 | 1.30 | 0.932 |  | 1.00 | 0.72 | 1.38 | 0.981 |  | 0.95 | 0.75 | 1.21 | 0.677 |  | 0.02 | -1.52 | 1.55 | 0.982 |
|  | Wave 10 * 3rd | 1.18 | 0.90 | 1.55 | 0.237 |  | 1.02 | 0.74 | 1.41 | 0.906 |  | 1.09 | 0.87 | 1.37 | 0.452 |  | 0.32 | -1.07 | 1.71 | 0.654 |
|  | Wave 10 * 4th | 1.20 | 0.88 | 1.64 | 0.248 |  | 0.95 | 0.69 | 1.31 | 0.769 |  | 1.13 | 0.89 | 1.44 | 0.311 |  | 0.90 | -0.49 | 2.29 | 0.203 |
|  | Wave 10 * 5th, Wealthiest | 1.02 | 0.72 | 1.46 | 0.898 |  | 1.06 | 0.77 | 1.46 | 0.742 |  | 0.99 | 0.77 | 1.29 | 0.959 |  | 0.59 | -0.71 | 1.89 | 0.376 |
|  | Age group (ref. 50-59) |  |  |  |  |  |  |  |  |  |  |  |  |  |  |  |  |  |  |  |
|  | 60-69 | 0.81 | 0.72 | 0.91 | <0.001 |  | 0.88 | 0.77 | 1.00 | 0.043 |  | 0.91 | 0.82 | 1.02 | 0.092 |  | 1.07 | 0.54 | 1.61 | <0.001 |
|  | 70-79 | 0.66 | 0.58 | 0.75 | <0.001 |  | 0.75 | 0.66 | 0.86 | <0.001 |  | 0.76 | 0.68 | 0.85 | <0.001 |  | 0.91 | 0.35 | 1.47 | 0.002 |
|  | 80+ | 0.81 | 0.70 | 0.93 | 0.003 |  | 0.68 | 0.58 | 0.80 | <0.001 |  | 0.88 | 0.77 | 1.00 | 0.049 |  | -0.91 | -1.59 | -0.23 | 0.009 |
|  | Gender: Women | 1.36 | 1.22 | 1.51 | <0.001 |  | 1.19 | 1.08 | 1.31 | 0.001 |  | 1.11 | 1.02 | 1.21 | 0.019 |  | 0.71 | 0.29 | 1.14 | 0.001 |
|  | Highest educational qualification (ref. Post-secondary) |  |  |  |  |  |  |  |  |  |  |  |  |  |  |  |  |  |  |  |
|  | A-level, O-Level, CSE, technical equivalent | 1.18 | 1.02 | 1.36 | 0.030 |  | 0.82 | 0.72 | 0.92 | 0.001 |  | 1.17 | 1.03 | 1.33 | 0.014 |  | -0.78 | -1.25 | -0.31 | 0.001 |
|  | No academic qualification | 1.37 | 1.16 | 1.61 | <0.001 |  | 1.01 | 0.85 | 1.18 | 0.941 |  | 1.28 | 1.11 | 1.49 | 0.001 |  | -1.54 | -2.22 | -0.86 | <0.001 |
|  | Chronic conditions (ref. No illness) |  |  |  |  |  |  |  |  |  |  |  |  |  |  |  |  |  |  |  |
|  | Non-limiting illness | 1.40 | 1.18 | 1.65 | <0.001 |  | 1.08 | 0.95 | 1.23 | 0.223 |  | 1.24 | 1.11 | 1.38 | <0.001 |  | -1.52 | -1.98 | -1.06 | <0.001 |
|  | Limiting illness | 3.75 | 3.28 | 4.28 | <0.001 |  | 1.59 | 1.43 | 1.78 | <0.001 |  | 1.84 | 1.67 | 2.02 | <0.001 |  | -7.55 | -8.03 | -7.06 | <0.001 |

Note. Weighted and adjusted models (adjusted for age group, gender, educational attainment, and chronic conditions; models by gender do not include educational attainment to avoid over-adjustment). B: regression coefficient; CASP-19: 19-item Control, Autonomy, Self-realisation and Pleasure; CESD-8: 8-item Centre for Epidemiologic Studies Depression Scale; LB: 95% confidence interval lower bound; ONS: Office for National Statistics; RR: risk ratio; UB: 95% confidence interval upper bound; UCLA-3: University of California Los Angeles 3-item loneliness scale. P-values for overall wave, wealth, and the interaction between wave and the grouping variable correspond to the omnibus test.

# Table S2. Extended results from the modified Poisson and linear regression models testing the statistical effect of time (wave), the grouping variables, and their interaction, weighted results, using imputed data.

|  |  | High depressive symptomatology  (CESD-8 ≥ 4) | | | |  | High anxiety  (ONS single question ≥ 6) | | | |  | High loneliness  (UCLA-3 ≥ 6) | | | |  | Quality of life  (CASP-19) | | | |
| --- | --- | --- | --- | --- | --- | --- | --- | --- | --- | --- | --- | --- | --- | --- | --- | --- | --- | --- | --- | --- |
|  | Variable | RR | LB | UB | p-value |  | RR | LB | UB | p-value |  | RR | LB | UB | p-value |  | B | LB | UB | p-value |
| Overall | Wave (ref. Wave 9) |  |  |  | <0.001 |  |  |  |  | 0.265 |  |  |  |  | <0.001 |  |  |  |  | <0.001 |
|  | Wave 8 | 1.01 | 0.93 | 1.10 | 0.812 |  | 1.02 | 0.92 | 1.12 | 0.706 |  | 0.95 | 0.88 | 1.02 | 0.171 |  | -0.30 | -0.63 | 0.03 | 0.075 |
|  | Wave 10 | 1.24 | 1.13 | 1.36 | <0.001 |  | 1.09 | 0.98 | 1.20 | 0.107 |  | 1.33 | 1.23 | 1.43 | <0.001 |  | -1.92 | -2.29 | -1.55 | <0.001 |
|  | Age group (ref. 50-59) |  |  |  |  |  |  |  |  |  |  |  |  |  |  |  |  |  |  |  |
|  | 60-69 | 0.73 | 0.65 | 0.82 | <0.001 |  | 0.84 | 0.74 | 0.94 | 0.003 |  | 0.85 | 0.76 | 0.94 | 0.002 |  | 1.89 | 1.32 | 2.45 | <0.001 |
|  | 70-79 | 0.55 | 0.49 | 0.62 | <0.001 |  | 0.71 | 0.63 | 0.80 | <0.001 |  | 0.69 | 0.61 | 0.77 | <0.001 |  | 2.03 | 1.43 | 2.63 | <0.001 |
|  | 80+ | 0.65 | 0.57 | 0.74 | <0.001 |  | 0.65 | 0.56 | 0.76 | <0.001 |  | 0.79 | 0.70 | 0.90 | <0.001 |  | 0.44 | -0.27 | 1.15 | 0.220 |
|  | Gender: Women | 1.38 | 1.24 | 1.53 | <0.001 |  | 1.16 | 1.05 | 1.28 | 0.004 |  | 1.09 | 1.00 | 1.18 | 0.061 |  | 0.70 | 0.25 | 1.14 | 0.002 |
|  | Highest educational qualification (ref. Post-secondary) |  |  |  |  |  |  |  |  |  |  |  |  |  |  |  |  |  |  |  |
|  | A-level, O-Level, CSE, technical equivalent | 1.43 | 1.24 | 1.66 | <0.001 |  | 0.87 | 0.77 | 0.98 | 0.026 |  | 1.32 | 1.17 | 1.49 | <0.001 |  | -1.86 | -2.37 | -1.35 | <0.001 |
|  | No academic qualification | 1.92 | 1.63 | 2.25 | <0.001 |  | 1.10 | 0.94 | 1.28 | 0.251 |  | 1.59 | 1.38 | 1.84 | <0.001 |  | -3.59 | -4.30 | -2.88 | <0.001 |
|  | Chronic conditions (ref. No illness) |  |  |  |  |  |  |  |  |  |  |  |  |  |  |  |  |  |  |  |
|  | Non-limiting illness | 1.54 | 1.30 | 1.82 | <0.001 |  | 1.15 | 1.00 | 1.32 | 0.042 |  | 1.26 | 1.13 | 1.41 | <0.001 |  | -1.84 | -2.36 | -1.32 | <0.001 |
|  | Limiting illness | 4.47 | 3.93 | 5.09 | <0.001 |  | 1.66 | 1.49 | 1.84 | <0.001 |  | 1.98 | 1.80 | 2.17 | <0.001 |  | -8.48 | -8.97 | -8.00 | <0.001 |
| By gender | Gender: Women | 1.63 | 1.39 | 1.91 | <0.001 |  | 1.17 | 1.01 | 1.35 | 0.041 |  | 1.19 | 1.05 | 1.35 | 0.006 |  | 0.33 | -0.21 | 0.88 | 0.233 |
|  | Wave (ref. Wave 9) |  |  |  | <0.001 |  |  |  |  | 0.712 |  |  |  |  | <0.001 |  |  |  |  | <0.001 |
|  | Wave 8 | 1.08 | 0.92 | 1.28 | 0.352 |  | 1.06 | 0.90 | 1.24 | 0.508 |  | 1.01 | 0.89 | 1.14 | 0.922 |  | -0.51 | -1.04 | 0.02 | 0.057 |
|  | Wave 10 | 1.50 | 1.26 | 1.79 | <0.001 |  | 1.06 | 0.90 | 1.26 | 0.475 |  | 1.41 | 1.24 | 1.60 | <0.001 |  | -1.97 | -2.54 | -1.41 | <0.001 |
|  | Wave (ref. Wave 9) * Gender (ref. Men) |  |  |  | 0.006 |  |  |  |  | 0.747 |  |  |  |  | 0.241 |  |  |  |  | 0.667 |
|  | Wave 8 * Women | 0.92 | 0.76 | 1.12 | 0.404 |  | 0.95 | 0.78 | 1.16 | 0.608 |  | 0.92 | 0.79 | 1.07 | 0.288 |  | 0.23 | -0.43 | 0.88 | 0.495 |
|  | Wave 10 * Women | 0.72 | 0.59 | 0.89 | 0.002 |  | 1.03 | 0.84 | 1.27 | 0.764 |  | 0.88 | 0.75 | 1.03 | 0.106 |  | 0.28 | -0.46 | 1.02 | 0.457 |
|  | Age group (ref. 50-59) |  |  |  |  |  |  |  |  |  |  |  |  |  |  |  |  |  |  |  |
|  | 60-69 | 0.73 | 0.65 | 0.82 | <0.001 |  | 0.84 | 0.75 | 0.95 | 0.005 |  | 0.85 | 0.77 | 0.95 | 0.003 |  | 1.83 | 1.26 | 2.40 | <0.001 |
|  | 70-79 | 0.58 | 0.51 | 0.65 | <0.001 |  | 0.73 | 0.64 | 0.82 | <0.001 |  | 0.71 | 0.64 | 0.79 | <0.001 |  | 1.72 | 1.13 | 2.31 | <0.001 |
|  | 80+ | 0.71 | 0.63 | 0.82 | <0.001 |  | 0.68 | 0.59 | 0.79 | <0.001 |  | 0.86 | 0.76 | 0.97 | 0.012 |  | -0.21 | -0.90 | 0.48 | 0.550 |
|  | Chronic conditions (ref. No illness) |  |  |  |  |  |  |  |  |  |  |  |  |  |  |  |  |  |  |  |
|  | Non-limiting illness | 1.54 | 1.30 | 1.83 | <0.001 |  | 1.15 | 1.00 | 1.31 | 0.043 |  | 1.26 | 1.12 | 1.41 | <0.001 |  | -1.82 | -2.35 | -1.29 | <0.001 |
|  | Limiting illness | 4.73 | 4.16 | 5.37 | <0.001 |  | 1.68 | 1.51 | 1.86 | <0.001 |  | 2.04 | 1.86 | 2.24 | <0.001 |  | -8.72 | -9.22 | -8.23 | <0.001 |
| By living situation | Living situation: Not living alone | 0.55 | 0.48 | 0.63 | <0.001 |  | 0.82 | 0.70 | 0.95 | 0.010 |  | 0.48 | 0.43 | 0.54 | <0.001 |  | 1.76 | 1.10 | 2.42 | <0.001 |
|  | Wave (ref. Wave 9) |  |  |  | 0.207 |  |  |  |  | 0.787 |  |  |  |  | <0.001 |  |  |  |  | <0.001 |
|  | Wave 8 | 1.01 | 0.90 | 1.14 | 0.834 |  | 0.97 | 0.82 | 1.14 | 0.681 |  | 0.95 | 0.86 | 1.04 | 0.258 |  | -0.18 | -0.83 | 0.47 | 0.587 |
|  | Wave 10 | 1.11 | 0.98 | 1.26 | 0.097 |  | 0.94 | 0.78 | 1.13 | 0.493 |  | 1.29 | 1.17 | 1.42 | <0.001 |  | -2.27 | -3.06 | -1.47 | <0.001 |
|  | Wave (ref. Wave 9) * Living situation (ref. Living alone) |  |  |  | 0.090 |  |  |  |  | 0.222 |  |  |  |  | 0.753 |  |  |  |  | 0.455 |
|  | Wave 8 * Not living alone | 1.01 | 0.85 | 1.20 | 0.918 |  | 1.08 | 0.88 | 1.32 | 0.485 |  | 1.01 | 0.87 | 1.17 | 0.885 |  | -0.16 | -0.93 | 0.62 | 0.691 |
|  | Wave 10 * Not living alone | 1.20 | 1.01 | 1.44 | 0.044 |  | 1.21 | 0.98 | 1.51 | 0.080 |  | 1.05 | 0.91 | 1.22 | 0.464 |  | 0.45 | -0.45 | 1.35 | 0.328 |
|  | Age group (ref. 50-59) |  |  |  |  |  |  |  |  |  |  |  |  |  |  |  |  |  |  |  |
|  | 60-69 | 0.72 | 0.64 | 0.80 | <0.001 |  | 0.83 | 0.74 | 0.94 | 0.003 |  | 0.82 | 0.74 | 0.91 | <0.001 |  | 1.96 | 1.40 | 2.52 | <0.001 |
|  | 70-79 | 0.53 | 0.47 | 0.60 | <0.001 |  | 0.70 | 0.62 | 0.80 | <0.001 |  | 0.65 | 0.58 | 0.72 | <0.001 |  | 2.17 | 1.58 | 2.77 | <0.001 |
|  | 80+ | 0.56 | 0.49 | 0.63 | <0.001 |  | 0.63 | 0.54 | 0.73 | <0.001 |  | 0.65 | 0.57 | 0.73 | <0.001 |  | 0.95 | 0.24 | 1.67 | 0.009 |
|  | Gender: Women | 1.36 | 1.22 | 1.51 | <0.001 |  | 1.15 | 1.04 | 1.27 | 0.005 |  | 1.05 | 0.97 | 1.15 | 0.217 |  | 0.79 | 0.35 | 1.24 | <0.001 |
|  | Highest educational qualification (ref. Post-secondary) |  |  |  |  |  |  |  |  |  |  |  |  |  |  |  |  |  |  |  |
|  | A-level, O-Level, CSE, technical equivalent | 1.42 | 1.22 | 1.64 | <0.001 |  | 0.87 | 0.77 | 0.98 | 0.025 |  | 1.30 | 1.15 | 1.47 | <0.001 |  | -1.83 | -2.34 | -1.32 | <0.001 |
|  | No academic qualification | 1.84 | 1.56 | 2.17 | <0.001 |  | 1.09 | 0.93 | 1.28 | 0.275 |  | 1.53 | 1.32 | 1.76 | <0.001 |  | -3.51 | -4.22 | -2.80 | <0.001 |
|  | Chronic conditions (ref. No illness) |  |  |  |  |  |  |  |  |  |  |  |  |  |  |  |  |  |  |  |
|  | Non-limiting illness | 1.51 | 1.27 | 1.79 | <0.001 |  | 1.15 | 1.00 | 1.31 | 0.048 |  | 1.23 | 1.10 | 1.38 | <0.001 |  | -1.80 | -2.32 | -1.27 | <0.001 |
|  | Limiting illness | 4.18 | 3.68 | 4.76 | <0.001 |  | 1.64 | 1.48 | 1.82 | <0.001 |  | 1.82 | 1.66 | 2.00 | <0.001 |  | -8.33 | -8.81 | -7.84 | <0.001 |
| By wealth quintile | Wealth quintile (ref. 1st, Poorest) |  |  |  | <0.001 |  |  |  |  | 0.150 |  |  |  |  | <0.001 |  |  |  |  | <0.001 |
|  | 2nd | 0.70 | 0.58 | 0.85 | <0.001 |  | 0.99 | 0.79 | 1.24 | 0.935 |  | 0.89 | 0.74 | 1.06 | 0.187 |  | 2.70 | 1.71 | 3.70 | <0.001 |
|  | 3rd | 0.55 | 0.45 | 0.67 | <0.001 |  | 0.85 | 0.68 | 1.07 | 0.167 |  | 0.74 | 0.62 | 0.88 | 0.001 |  | 3.93 | 3.01 | 4.85 | <0.001 |
|  | 4th | 0.48 | 0.38 | 0.61 | <0.001 |  | 0.87 | 0.69 | 1.11 | 0.262 |  | 0.63 | 0.52 | 0.77 | <0.001 |  | 4.90 | 3.97 | 5.83 | <0.001 |
|  | 5th, Wealthiest | 0.37 | 0.29 | 0.48 | <0.001 |  | 0.77 | 0.60 | 0.97 | 0.027 |  | 0.56 | 0.45 | 0.70 | <0.001 |  | 5.82 | 4.93 | 6.70 | <0.001 |
|  | Wave (ref. Wave 9) |  |  |  | 0.081 |  |  |  |  | 0.352 |  |  |  |  | 0.002 |  |  |  |  | <0.001 |
|  | Wave 8 | 1.13 | 0.97 | 1.32 | 0.108 |  | 1.16 | 0.95 | 1.42 | 0.145 |  | 1.03 | 0.88 | 1.20 | 0.740 |  | -1.12 | -2.21 | -0.02 | 0.045 |
|  | Wave 10 | 1.18 | 1.01 | 1.39 | 0.040 |  | 1.07 | 0.85 | 1.35 | 0.569 |  | 1.31 | 1.12 | 1.53 | 0.001 |  | -2.34 | -3.49 | -1.19 | <0.001 |
|  | Wave (ref. Wave 9) * Wealth quintile (ref. 1st, Poorest) |  |  |  | 0.336 |  |  |  |  | 0.889 |  |  |  |  | 0.802 |  |  |  |  | 0.638 |
|  | Wave 8 * 2nd | 0.87 | 0.67 | 1.13 | 0.295 |  | 0.87 | 0.65 | 1.18 | 0.370 |  | 0.90 | 0.70 | 1.15 | 0.394 |  | 0.76 | -0.71 | 2.23 | 0.309 |
|  | Wave 8 * 3rd | 0.99 | 0.75 | 1.30 | 0.931 |  | 1.01 | 0.73 | 1.40 | 0.966 |  | 0.95 | 0.75 | 1.21 | 0.698 |  | 0.13 | -1.42 | 1.68 | 0.869 |
|  | Wave 8 * 4th | 0.87 | 0.66 | 1.14 | 0.319 |  | 0.81 | 0.59 | 1.10 | 0.179 |  | 0.91 | 0.71 | 1.17 | 0.467 |  | 1.00 | -0.32 | 2.31 | 0.139 |
|  | Wave 8 * 5th, Wealthiest | 1.16 | 0.88 | 1.53 | 0.283 |  | 1.05 | 0.75 | 1.45 | 0.792 |  | 1.09 | 0.87 | 1.36 | 0.466 |  | 0.39 | -1.02 | 1.81 | 0.586 |
|  | Wave 10 * 2nd | 0.77 | 0.56 | 1.06 | 0.112 |  | 0.82 | 0.60 | 1.12 | 0.203 |  | 0.89 | 0.70 | 1.14 | 0.370 |  | 1.04 | -0.22 | 2.30 | 0.107 |
|  | Wave 10 * 3rd | 1.16 | 0.84 | 1.58 | 0.367 |  | 0.97 | 0.70 | 1.33 | 0.830 |  | 1.08 | 0.85 | 1.38 | 0.509 |  | 0.94 | -0.45 | 2.33 | 0.185 |
|  | Wave 10 * 4th | 0.82 | 0.58 | 1.16 | 0.265 |  | 0.87 | 0.65 | 1.16 | 0.339 |  | 0.90 | 0.69 | 1.17 | 0.425 |  | 1.11 | -0.13 | 2.36 | 0.079 |
|  | Wave 10 * 5th, Wealthiest | 1.00 | 0.70 | 1.43 | 0.995 |  | 1.06 | 0.77 | 1.47 | 0.714 |  | 0.94 | 0.72 | 1.21 | 0.621 |  | 0.82 | -0.52 | 2.16 | 0.230 |
|  | Age group (ref. 50-59) |  |  |  |  |  |  |  |  |  |  |  |  |  |  |  |  |  |  |  |
|  | 60-69 | 0.83 | 0.74 | 0.92 | 0.001 |  | 0.87 | 0.77 | 0.98 | 0.026 |  | 0.92 | 0.83 | 1.02 | 0.107 |  | 0.97 | 0.45 | 1.49 | <0.001 |
|  | 70-79 | 0.67 | 0.59 | 0.77 | <0.001 |  | 0.75 | 0.66 | 0.85 | <0.001 |  | 0.77 | 0.69 | 0.86 | <0.001 |  | 0.77 | 0.22 | 1.33 | 0.006 |
|  | 80+ | 0.82 | 0.71 | 0.94 | 0.005 |  | 0.69 | 0.59 | 0.81 | <0.001 |  | 0.89 | 0.79 | 1.01 | 0.068 |  | -0.93 | -1.60 | -0.26 | 0.007 |
|  | Gender: Women | 1.37 | 1.23 | 1.52 | <0.001 |  | 1.15 | 1.04 | 1.27 | 0.005 |  | 1.08 | 0.99 | 1.18 | 0.073 |  | 0.75 | 0.33 | 1.17 | <0.001 |
|  | Highest educational qualification (ref. Post-secondary) |  |  |  |  |  |  |  |  |  |  |  |  |  |  |  |  |  |  |  |
|  | A-level, O-Level, CSE, technical equivalent | 1.19 | 1.03 | 1.38 | 0.017 |  | 0.83 | 0.73 | 0.93 | 0.002 |  | 1.17 | 1.03 | 1.33 | 0.013 |  | -0.78 | -1.26 | -0.29 | 0.002 |
|  | No academic qualification | 1.39 | 1.18 | 1.64 | <0.001 |  | 0.99 | 0.84 | 1.17 | 0.896 |  | 1.30 | 1.12 | 1.51 | 0.001 |  | -1.62 | -2.32 | -0.93 | <0.001 |
|  | Chronic conditions (ref. No illness) |  |  |  |  |  |  |  |  |  |  |  |  |  |  |  |  |  |  |  |
|  | Non-limiting illness | 1.46 | 1.24 | 1.73 | <0.001 |  | 1.14 | 1.00 | 1.30 | 0.056 |  | 1.23 | 1.10 | 1.38 | <0.001 |  | -1.56 | -2.05 | -1.07 | <0.001 |
|  | Limiting illness | 3.80 | 3.33 | 4.33 | <0.001 |  | 1.59 | 1.42 | 1.76 | <0.001 |  | 1.80 | 1.64 | 1.98 | <0.001 |  | -7.48 | -7.95 | -7.01 | <0.001 |

Note. Models conducted with imputed data. Weighted and adjusted models (adjusted for age group, gender, educational attainment, and chronic conditions; models by gender do not include educational attainment to avoid over-adjustment). B: regression coefficient; CASP-19: 19-item Control, Autonomy, Self-realisation and Pleasure; CESD-8: 8-item Centre for Epidemiologic Studies Depression Scale; LB: 95% confidence interval lower bound; ONS: Office for National Statistics; RR: risk ratio; UB: 95% confidence interval upper bound; UCLA-3: University of California Los Angeles 3-item loneliness scale. P-values for overall wave, wealth, and the interaction between wave and the grouping variable correspond to the omnibus test.

# Table S3. Results from the modified Poisson and linear regression models excluding participants interviewed during the temporary restrictions between November 2021 and February 2022, using a non-face-to-face mode, or without a COVID-19 booster vaccine. Weighted results.

|  |  | High depressive symptomatology  (CESD-8 ≥ 4) | | | |  | High anxiety  (ONS single question ≥ 6) | | | |  | High loneliness  (UCLA-3 ≥ 6) | | | |  | Quality of life  (CASP-19) | | | |
| --- | --- | --- | --- | --- | --- | --- | --- | --- | --- | --- | --- | --- | --- | --- | --- | --- | --- | --- | --- | --- |
|  | Variable | RR | LB | UB | p-value |  | RR | LB | UB | p-value |  | RR | LB | UB | p-value |  | B | LB | UB | p-value |
| Overall | Wave (ref. Wave 9) |  |  |  | 0.005 |  |  |  |  | 0.220 |  |  |  |  | <0.001 |  |  |  |  | <0.001 |
|  | Wave 8 | 1.02 | 0.93 | 1.11 | 0.686 |  | 1.02 | 0.92 | 1.12 | 0.708 |  | 0.95 | 0.88 | 1.03 | 0.207 |  | -0.28 | -0.60 | 0.04 | 0.087 |
|  | Wave 10 | 1.18 | 1.07 | 1.31 | 0.002 |  | 1.10 | 0.99 | 1.23 | 0.086 |  | 1.32 | 1.21 | 1.43 | <0.001 |  | -1.90 | -2.32 | -1.48 | <0.001 |
|  | Age group (ref. 50-59) |  |  |  |  |  |  |  |  |  |  |  |  |  |  |  |  |  |  |  |
|  | 60-69 | 0.71 | 0.63 | 0.80 | <0.001 |  | 0.82 | 0.72 | 0.92 | 0.001 |  | 0.85 | 0.76 | 0.95 | 0.004 |  | 2.11 | 1.54 | 2.68 | <0.001 |
|  | 70-79 | 0.54 | 0.47 | 0.62 | <0.001 |  | 0.69 | 0.61 | 0.79 | <0.001 |  | 0.69 | 0.61 | 0.77 | <0.001 |  | 2.17 | 1.57 | 2.77 | <0.001 |
|  | 80+ | 0.64 | 0.56 | 0.74 | <0.001 |  | 0.63 | 0.54 | 0.74 | <0.001 |  | 0.79 | 0.69 | 0.90 | <0.001 |  | 0.53 | -0.19 | 1.24 | 0.148 |
|  | Gender: Women | 1.39 | 1.24 | 1.56 | <0.001 |  | 1.20 | 1.08 | 1.33 | <0.001 |  | 1.11 | 1.02 | 1.22 | 0.020 |  | 0.64 | 0.20 | 1.09 | 0.005 |
|  | Highest educational qualification (ref. Post-secondary) |  |  |  |  |  |  |  |  |  |  |  |  |  |  |  |  |  |  |  |
|  | A-level, O-Level, CSE, technical equivalent | 1.38 | 1.18 | 1.61 | <0.001 |  | 0.86 | 0.76 | 0.97 | 0.012 |  | 1.34 | 1.17 | 1.53 | <0.001 |  | -1.75 | -2.25 | -1.25 | <0.001 |
|  | No academic qualification | 1.89 | 1.60 | 2.24 | <0.001 |  | 1.11 | 0.95 | 1.30 | 0.194 |  | 1.60 | 1.37 | 1.86 | <0.001 |  | -3.46 | -4.17 | -2.75 | <0.001 |
|  | Chronic conditions (ref. No illness) |  |  |  |  |  |  |  |  |  |  |  |  |  |  |  |  |  |  |  |
|  | Non-limiting illness | 1.48 | 1.24 | 1.76 | <0.001 |  | 1.10 | 0.97 | 1.26 | 0.141 |  | 1.27 | 1.13 | 1.43 | <0.001 |  | -1.86 | -2.34 | -1.37 | <0.001 |
|  | Limiting illness | 4.36 | 3.81 | 5.00 | <0.001 |  | 1.66 | 1.49 | 1.86 | <0.001 |  | 2.02 | 1.84 | 2.23 | <0.001 |  | -8.48 | -8.98 | -7.98 | <0.001 |
| By gender | Gender: Women | 1.64 | 1.40 | 1.93 | <0.001 |  | 1.17 | 1.01 | 1.35 | 0.037 |  | 1.17 | 1.03 | 1.33 | 0.013 |  | 0.47 | -0.08 | 1.03 | 0.094 |
|  | Wave (ref. Wave 9) |  |  |  | <0.001 |  |  |  |  | 0.808 |  |  |  |  | <0.001 |  |  |  |  | <0.001 |
|  | Wave 8 | 1.09 | 0.93 | 1.29 | 0.295 |  | 1.05 | 0.90 | 1.23 | 0.529 |  | 1.00 | 0.88 | 1.13 | 0.969 |  | -0.48 | -1.01 | 0.05 | 0.077 |
|  | Wave 10 | 1.47 | 1.21 | 1.80 | <0.001 |  | 1.04 | 0.86 | 1.25 | 0.701 |  | 1.41 | 1.22 | 1.62 | <0.001 |  | -1.98 | -2.63 | -1.33 | <0.001 |
|  | Wave (ref. Wave 9) * Gender (ref. Men) |  |  |  | 0.012 |  |  |  |  | 0.531 |  |  |  |  | 0.357 |  |  |  |  | 0.852 |
|  | Wave 8 * Women | 0.91 | 0.75 | 1.10 | 0.341 |  | 0.94 | 0.77 | 1.14 | 0.539 |  | 0.94 | 0.80 | 1.09 | 0.388 |  | 0.18 | -0.48 | 0.84 | 0.592 |
|  | Wave 10 * Women | 0.70 | 0.56 | 0.89 | 0.003 |  | 1.08 | 0.86 | 1.36 | 0.514 |  | 0.89 | 0.75 | 1.05 | 0.168 |  | 0.13 | -0.72 | 0.99 | 0.759 |
|  | Age group (ref. 50-59) |  |  |  |  |  |  |  |  |  |  |  |  |  |  |  |  |  |  |  |
|  | 60-69 | 0.74 | 0.65 | 0.83 | <0.001 |  | 0.82 | 0.73 | 0.93 | 0.002 |  | 0.87 | 0.77 | 0.97 | 0.012 |  | 1.90 | 1.31 | 2.50 | <0.001 |
|  | 70-79 | 0.59 | 0.51 | 0.67 | <0.001 |  | 0.71 | 0.62 | 0.80 | <0.001 |  | 0.72 | 0.64 | 0.81 | <0.001 |  | 1.75 | 1.13 | 2.36 | <0.001 |
|  | 80+ | 0.73 | 0.64 | 0.84 | <0.001 |  | 0.66 | 0.57 | 0.77 | <0.001 |  | 0.86 | 0.76 | 0.98 | 0.023 |  | -0.24 | -0.95 | 0.48 | 0.515 |
|  | Chronic conditions (ref. No illness) |  |  |  |  |  |  |  |  |  |  |  |  |  |  |  |  |  |  |  |
|  | Non-limiting illness | 1.55 | 1.29 | 1.85 | <0.001 |  | 1.15 | 1.00 | 1.32 | 0.055 |  | 1.28 | 1.13 | 1.44 | <0.001 |  | -1.89 | -2.46 | -1.32 | <0.001 |
|  | Limiting illness | 4.71 | 4.12 | 5.39 | <0.001 |  | 1.67 | 1.50 | 1.86 | <0.001 |  | 2.08 | 1.89 | 2.29 | <0.001 |  | -8.63 | -9.14 | -8.12 | <0.001 |
| By living situation | Living situation: Not living alone | 0.55 | 0.48 | 0.63 | <0.001 |  | 0.82 | 0.70 | 0.96 | 0.013 |  | 0.49 | 0.43 | 0.55 | <0.001 |  | 1.78 | 1.14 | 2.43 | <0.001 |
|  | Wave (ref. Wave 9) |  |  |  | 0.767 |  |  |  |  | 0.891 |  |  |  |  | <0.001 |  |  |  |  | <0.001 |
|  | Wave 8 | 1.02 | 0.91 | 1.15 | 0.740 |  | 0.98 | 0.83 | 1.16 | 0.826 |  | 0.96 | 0.87 | 1.05 | 0.360 |  | -0.28 | -0.92 | 0.37 | 0.398 |
|  | Wave 10 | 1.05 | 0.91 | 1.22 | 0.467 |  | 1.03 | 0.85 | 1.25 | 0.775 |  | 1.32 | 1.19 | 1.46 | <0.001 |  | -2.07 | -2.94 | -1.21 | <0.001 |
|  | Wave (ref. Wave 9) * Living situation (ref. Living alone) |  |  |  | 0.138 |  |  |  |  | 0.726 |  |  |  |  | 0.998 |  |  |  |  | 0.890 |
|  | Wave 8 * Not living alone | 1.00 | 0.84 | 1.19 | 0.975 |  | 1.05 | 0.86 | 1.29 | 0.621 |  | 1.00 | 0.86 | 1.15 | 0.953 |  | 0.01 | -0.74 | 0.76 | 0.982 |
|  | Wave 10 * Not living alone | 1.21 | 0.99 | 1.49 | 0.065 |  | 1.10 | 0.87 | 1.39 | 0.436 |  | 1.00 | 0.85 | 1.17 | 0.996 |  | 0.23 | -0.76 | 1.23 | 0.644 |
|  | Age group (ref. 50-59) |  |  |  |  |  |  |  |  |  |  |  |  |  |  |  |  |  |  |  |
|  | 60-69 | 0.70 | 0.62 | 0.79 | <0.001 |  | 0.81 | 0.72 | 0.92 | 0.001 |  | 0.82 | 0.73 | 0.92 | <0.001 |  | 2.19 | 1.62 | 2.75 | <0.001 |
|  | 70-79 | 0.52 | 0.46 | 0.59 | <0.001 |  | 0.68 | 0.60 | 0.78 | <0.001 |  | 0.64 | 0.57 | 0.72 | <0.001 |  | 2.34 | 1.74 | 2.94 | <0.001 |
|  | 80+ | 0.54 | 0.47 | 0.62 | <0.001 |  | 0.60 | 0.51 | 0.71 | <0.001 |  | 0.63 | 0.56 | 0.72 | <0.001 |  | 1.06 | 0.34 | 1.78 | 0.004 |
|  | Gender: Women | 1.36 | 1.22 | 1.53 | <0.001 |  | 1.19 | 1.07 | 1.32 | 0.001 |  | 1.07 | 0.98 | 1.18 | 0.112 |  | 0.75 | 0.30 | 1.20 | 0.001 |
|  | Highest educational qualification (ref. Post-secondary) |  |  |  |  |  |  |  |  |  |  |  |  |  |  |  |  |  |  |  |
|  | A-level, O-Level, CSE, technical equivalent | 1.36 | 1.17 | 1.59 | <0.001 |  | 0.85 | 0.76 | 0.96 | 0.011 |  | 1.33 | 1.16 | 1.51 | <0.001 |  | -1.74 | -2.24 | -1.24 | <0.001 |
|  | No academic qualification | 1.82 | 1.53 | 2.15 | <0.001 |  | 1.10 | 0.94 | 1.29 | 0.220 |  | 1.54 | 1.32 | 1.79 | <0.001 |  | -3.38 | -4.09 | -2.67 | <0.001 |
|  | Chronic conditions (ref. No illness) |  |  |  |  |  |  |  |  |  |  |  |  |  |  |  |  |  |  |  |
|  | Non-limiting illness | 1.45 | 1.22 | 1.72 | <0.001 |  | 1.10 | 0.96 | 1.25 | 0.156 |  | 1.24 | 1.11 | 1.39 | <0.001 |  | -1.81 | -2.29 | -1.33 | <0.001 |
|  | Limiting illness | 4.08 | 3.57 | 4.67 | <0.001 |  | 1.64 | 1.47 | 1.83 | <0.001 |  | 1.86 | 1.69 | 2.05 | <0.001 |  | -8.34 | -8.83 | -7.84 | <0.001 |
| By wealth quintile | Wealth quintile (ref. 1st, Poorest) |  |  |  | <0.001 |  |  |  |  | 0.259 |  |  |  |  | <0.001 |  |  |  |  | <0.001 |
|  | 2nd | 0.70 | 0.58 | 0.85 | <0.001 |  | 1.03 | 0.82 | 1.29 | 0.808 |  | 0.89 | 0.74 | 1.07 | 0.214 |  | 2.41 | 1.37 | 3.44 | <0.001 |
|  | 3rd | 0.54 | 0.44 | 0.66 | <0.001 |  | 0.89 | 0.71 | 1.12 | 0.320 |  | 0.75 | 0.63 | 0.89 | 0.001 |  | 3.67 | 2.72 | 4.61 | <0.001 |
|  | 4th | 0.47 | 0.37 | 0.60 | <0.001 |  | 0.90 | 0.71 | 1.15 | 0.400 |  | 0.64 | 0.52 | 0.77 | <0.001 |  | 4.57 | 3.61 | 5.53 | <0.001 |
|  | 5th, Wealthiest | 0.36 | 0.28 | 0.47 | <0.001 |  | 0.80 | 0.63 | 1.02 | 0.075 |  | 0.55 | 0.44 | 0.68 | <0.001 |  | 5.63 | 4.73 | 6.52 | <0.001 |
|  | Wave (ref. Wave 9) |  |  |  | 0.310 |  |  |  |  | 0.295 |  |  |  |  | 0.008 |  |  |  |  | <0.001 |
|  | Wave 8 | 1.12 | 0.97 | 1.30 | 0.134 |  | 1.17 | 0.95 | 1.44 | 0.147 |  | 1.03 | 0.88 | 1.21 | 0.691 |  | -0.99 | -2.05 | 0.07 | 0.067 |
|  | Wave 10 | 1.08 | 0.89 | 1.31 | 0.429 |  | 1.15 | 0.90 | 1.48 | 0.269 |  | 1.31 | 1.10 | 1.55 | 0.002 |  | -2.61 | -3.85 | -1.37 | <0.001 |
|  | Wave (ref. Wave 9) * Wealth quintile (ref. 1st, Poorest) |  |  |  | 0.202 |  |  |  |  | 0.879 |  |  |  |  | 0.814 |  |  |  |  | 0.462 |
|  | Wave 8 * 2nd | 0.88 | 0.68 | 1.13 | 0.306 |  | 0.84 | 0.62 | 1.13 | 0.256 |  | 0.92 | 0.71 | 1.18 | 0.502 |  | 0.65 | -0.82 | 2.13 | 0.384 |
|  | Wave 8 * 3rd | 1.06 | 0.78 | 1.45 | 0.701 |  | 0.92 | 0.64 | 1.30 | 0.626 |  | 0.97 | 0.75 | 1.26 | 0.840 |  | 0.52 | -1.14 | 2.18 | 0.541 |
|  | Wave 8 * 4th | 0.91 | 0.70 | 1.19 | 0.498 |  | 0.85 | 0.62 | 1.16 | 0.310 |  | 0.89 | 0.70 | 1.13 | 0.332 |  | 0.82 | -0.49 | 2.12 | 0.220 |
|  | Wave 8 * 5th, Wealthiest | 1.29 | 0.95 | 1.75 | 0.098 |  | 1.05 | 0.74 | 1.50 | 0.772 |  | 1.11 | 0.87 | 1.42 | 0.398 |  | 0.50 | -1.05 | 2.05 | 0.527 |
|  | Wave 10 * 2nd | 0.80 | 0.58 | 1.11 | 0.184 |  | 0.84 | 0.61 | 1.15 | 0.273 |  | 0.89 | 0.69 | 1.14 | 0.357 |  | 1.01 | -0.23 | 2.25 | 0.111 |
|  | Wave 10 * 3rd | 1.33 | 0.95 | 1.86 | 0.103 |  | 0.86 | 0.60 | 1.22 | 0.385 |  | 1.10 | 0.84 | 1.43 | 0.498 |  | 1.37 | -0.16 | 2.89 | 0.079 |
|  | Wave 10 * 4th | 0.89 | 0.63 | 1.25 | 0.488 |  | 0.85 | 0.63 | 1.14 | 0.275 |  | 0.93 | 0.71 | 1.21 | 0.569 |  | 0.89 | -0.31 | 2.09 | 0.147 |
|  | Wave 10 * 5th, Wealthiest | 1.07 | 0.70 | 1.64 | 0.739 |  | 0.95 | 0.66 | 1.35 | 0.761 |  | 0.99 | 0.74 | 1.32 | 0.923 |  | 1.40 | -0.05 | 2.84 | 0.058 |
|  | Age group (ref. 50-59) |  |  |  |  |  |  |  |  |  |  |  |  |  |  |  |  |  |  |  |
|  | 60-69 | 0.82 | 0.72 | 0.92 | 0.001 |  | 0.85 | 0.75 | 0.97 | 0.016 |  | 0.92 | 0.82 | 1.03 | 0.134 |  | 1.19 | 0.64 | 1.74 | <0.001 |
|  | 70-79 | 0.67 | 0.58 | 0.77 | <0.001 |  | 0.73 | 0.64 | 0.84 | <0.001 |  | 0.77 | 0.68 | 0.86 | <0.001 |  | 0.93 | 0.35 | 1.51 | 0.002 |
|  | 80+ | 0.81 | 0.70 | 0.94 | 0.006 |  | 0.66 | 0.56 | 0.79 | <0.001 |  | 0.88 | 0.77 | 1.01 | 0.069 |  | -0.81 | -1.51 | -0.11 | 0.023 |
|  | Gender: Women | 1.37 | 1.23 | 1.54 | <0.001 |  | 1.20 | 1.08 | 1.33 | 0.001 |  | 1.11 | 1.02 | 1.22 | 0.020 |  | 0.72 | 0.28 | 1.15 | 0.001 |
|  | Highest educational qualification (ref. Post-secondary) |  |  |  |  |  |  |  |  |  |  |  |  |  |  |  |  |  |  |  |
|  | A-level, O-Level, CSE, technical equivalent | 1.14 | 0.98 | 1.33 | 0.098 |  | 0.81 | 0.72 | 0.92 | 0.001 |  | 1.18 | 1.03 | 1.35 | 0.017 |  | -0.68 | -1.16 | -0.20 | 0.006 |
|  | No academic qualification | 1.36 | 1.14 | 1.62 | 0.001 |  | 1.02 | 0.86 | 1.21 | 0.826 |  | 1.27 | 1.09 | 1.49 | 0.003 |  | -1.42 | -2.11 | -0.72 | <0.001 |
|  | Chronic conditions (ref. No illness) |  |  |  |  |  |  |  |  |  |  |  |  |  |  |  |  |  |  |  |
|  | Non-limiting illness | 1.39 | 1.16 | 1.65 | <0.001 |  | 1.08 | 0.95 | 1.24 | 0.229 |  | 1.24 | 1.11 | 1.39 | <0.001 |  | -1.54 | -2.02 | -1.07 | <0.001 |
|  | Limiting illness | 3.69 | 3.21 | 4.24 | <0.001 |  | 1.58 | 1.41 | 1.76 | <0.001 |  | 1.85 | 1.67 | 2.04 | <0.001 |  | -7.51 | -8.00 | -7.02 | <0.001 |

Note. Weighted and adjusted models (adjusted for age group, gender, educational attainment, and chronic conditions; models by gender do not include educational attainment to avoid over-adjustment). B: regression coefficient; CASP-19: 19-item Control, Autonomy, Self-realisation and Pleasure; CESD-8: 8-item Centre for Epidemiologic Studies Depression Scale; LB: 95% confidence interval lower bound; ONS: Office for National Statistics; RR: risk ratio; UB: 95% confidence interval upper bound; UCLA-3: University of California Los Angeles 3-item loneliness scale. P-values for overall wave, wealth, and the interaction between wave and the grouping variable correspond to the omnibus test.

# Table S4. Results from the models by gender including educational attainment as part of the adjustment set. Weighted results.

|  | High depressive symptomatology  (CESD-8 ≥ 4) | | | |  | High anxiety  (ONS single question ≥ 6) | | | |  | High loneliness  (UCLA-3 ≥ 6) | | | |  | Quality of life  (CASP-19) | | | |
| --- | --- | --- | --- | --- | --- | --- | --- | --- | --- | --- | --- | --- | --- | --- | --- | --- | --- | --- | --- |
| Variable | RR | LB | UB | p-value |  | RR | LB | UB | p-value |  | RR | LB | UB | p-value |  | B | LB | UB | p-value |
| Gender: Women | 1.59 | 1.36 | 1.86 | <0.001 |  | 1.20 | 1.03 | 1.39 | 0.016 |  | 1.16 | 1.02 | 1.32 | 0.020 |  | 0.58 | 0.03 | 1.13 | 0.038 |
| Wave (ref. Wave 9) |  |  |  | <0.001 |  |  |  |  | 0.646 |  |  |  |  | <0.001 |  |  |  |  | <0.001 |
| Wave 8 | 1.08 | 0.93 | 1.27 | 0.315 |  | 1.04 | 0.88 | 1.22 | 0.664 |  | 0.97 | 0.86 | 1.09 | 0.578 |  | -0.35 | -0.86 | 0.15 | 0.170 |
| Wave 10 | 1.50 | 1.26 | 1.79 | <0.001 |  | 1.08 | 0.92 | 1.28 | 0.350 |  | 1.40 | 1.23 | 1.60 | <0.001 |  | -1.87 | -2.42 | -1.33 | <0.001 |
| Wave (ref. Wave 9) * Gender (ref. Men) |  |  |  | 0.007 |  |  |  |  | 0.935 |  |  |  |  | 0.386 |  |  |  |  | 0.889 |
| Wave 8 * Women | 0.91 | 0.76 | 1.10 | 0.326 |  | 0.97 | 0.80 | 1.18 | 0.754 |  | 0.98 | 0.84 | 1.14 | 0.764 |  | 0.16 | -0.48 | 0.79 | 0.628 |
| Wave 10 * Women | 0.73 | 0.59 | 0.89 | 0.002 |  | 1.00 | 0.82 | 1.23 | 0.970 |  | 0.90 | 0.77 | 1.05 | 0.174 |  | 0.06 | -0.67 | 0.79 | 0.875 |
| Age group (ref. 50-59) |  |  |  |  |  |  |  |  |  |  |  |  |  |  |  |  |  |  |  |
| 60-69 | 0.70 | 0.63 | 0.79 | <0.001 |  | 0.84 | 0.74 | 0.94 | 0.003 |  | 0.84 | 0.76 | 0.94 | 0.001 |  | 2.00 | 1.44 | 2.55 | <0.001 |
| 70-79 | 0.53 | 0.47 | 0.60 | <0.001 |  | 0.71 | 0.63 | 0.81 | <0.001 |  | 0.68 | 0.61 | 0.76 | <0.001 |  | 2.16 | 1.57 | 2.74 | <0.001 |
| 80+ | 0.63 | 0.55 | 0.72 | <0.001 |  | 0.64 | 0.55 | 0.75 | <0.001 |  | 0.78 | 0.69 | 0.89 | <0.001 |  | 0.45 | -0.24 | 1.15 | 0.204 |
| Highest educational qualification (ref. Post-secondary) |  |  |  |  |  |  |  |  |  |  |  |  |  |  |  |  |  |  |  |
| A-level, O-Level, CSE, technical equivalent | 1.42 | 1.23 | 1.64 | <0.001 |  | 0.86 | 0.76 | 0.96 | 0.009 |  | 1.32 | 1.17 | 1.50 | <0.001 |  | -1.81 | -2.30 | -1.33 | <0.001 |
| No academic qualification | 1.89 | 1.61 | 2.22 | <0.001 |  | 1.09 | 0.94 | 1.27 | 0.263 |  | 1.59 | 1.38 | 1.83 | <0.001 |  | -3.50 | -4.19 | -2.82 | <0.001 |
| Chronic conditions (ref. No illness) |  |  |  |  |  |  |  |  |  |  |  |  |  |  |  |  |  |  |  |
| Non-limiting illness | 1.51 | 1.28 | 1.78 | <0.001 |  | 1.11 | 0.98 | 1.26 | 0.108 |  | 1.26 | 1.13 | 1.41 | <0.001 |  | -1.83 | -2.30 | -1.37 | <0.001 |
| Limiting illness | 4.47 | 3.93 | 5.09 | <0.001 |  | 1.67 | 1.50 | 1.86 | <0.001 |  | 2.01 | 1.83 | 2.20 | <0.001 |  | -8.50 | -9.00 | -8.01 | <0.001 |

Note. Weighted and adjusted models. B: regression coefficient; CASP-19: 19-item Control, Autonomy, Self-realisation and Pleasure; CESD-8: 8-item Centre for Epidemiologic Studies Depression Scale; LB: 95% confidence interval lower bound; ONS: Office for National Statistics; RR: risk ratio; UB: 95% confidence interval upper bound; UCLA-3: University of California Los Angeles 3-item loneliness scale. P-values for overall wave, wealth, and the interaction between wave and the grouping variable correspond to the omnibus test.

# Table S5. Results from multinomial models with anxiety as a multi-categorical outcome. Weighted results.

|  |  | Reference group: Very low anxiety (ONS single question = 0 to 1) | | | | | | | | | | | | | |
| --- | --- | --- | --- | --- | --- | --- | --- | --- | --- | --- | --- | --- | --- | --- | --- |
|  |  | Low anxiety (2 to 3) | | | |  | Medium anxiety (4 to 5) | | | |  | High anxiety (6 to 10) | | | |
|  | Variable | RRR | LB | UB | p-value |  | RRR | LB | UB | p-value |  | RRR | LB | UB | p-value |
| Overall | Wave (ref. Wave 9) |  |  |  | <0.001 |  |  |  |  | <0.001 |  |  |  |  | 0.011 |
|  | Wave 8 | 1.32 | 1.18 | 1.46 | <0.001 |  | 1.45 | 1.27 | 1.65 | <0.001 |  | 1.19 | 1.05 | 1.34 | 0.005 |
|  | Wave 10 | 1.04 | 0.93 | 1.16 | 0.546 |  | 1.16 | 1.00 | 1.34 | 0.049 |  | 1.15 | 1.01 | 1.31 | 0.033 |
|  | Age group (ref. 50-59) |  |  |  |  |  |  |  |  |  |  |  |  |  |  |
|  | 60-69 | 0.77 | 0.67 | 0.88 | <0.001 |  | 0.64 | 0.54 | 0.77 | <0.001 |  | 0.68 | 0.58 | 0.80 | <0.001 |
|  | 70-79 | 0.74 | 0.64 | 0.85 | <0.001 |  | 0.56 | 0.46 | 0.67 | <0.001 |  | 0.54 | 0.45 | 0.64 | <0.001 |
|  | 80+ | 0.63 | 0.53 | 0.75 | <0.001 |  | 0.55 | 0.45 | 0.69 | <0.001 |  | 0.45 | 0.37 | 0.56 | <0.001 |
|  | Gender: Women | 1.07 | 0.96 | 1.19 | 0.220 |  | 1.17 | 1.02 | 1.34 | 0.025 |  | 1.30 | 1.14 | 1.48 | <0.001 |
|  | Highest educational qualification (ref. Post-secondary) |  |  |  |  |  |  |  |  |  |  |  |  |  |  |
|  | A-level, O-Level, CSE, technical equivalent | 0.71 | 0.62 | 0.80 | <0.001 |  | 0.94 | 0.79 | 1.11 | 0.445 |  | 0.73 | 0.62 | 0.86 | <0.001 |
|  | No academic qualification | 0.56 | 0.47 | 0.67 | <0.001 |  | 1.09 | 0.88 | 1.35 | 0.431 |  | 0.96 | 0.78 | 1.19 | 0.705 |
|  | Chronic conditions (ref. No illness) |  |  |  |  |  |  |  |  |  |  |  |  |  |  |
|  | Non-limiting illness | 1.16 | 1.02 | 1.32 | 0.023 |  | 1.18 | 1.00 | 1.38 | 0.050 |  | 1.22 | 1.04 | 1.43 | 0.016 |
|  | Limiting illness | 1.22 | 1.09 | 1.38 | 0.001 |  | 1.94 | 1.67 | 2.25 | <0.001 |  | 2.27 | 1.97 | 2.62 | <0.001 |
| By gender | Gender: Women | 0.95 | 0.82 | 1.11 | 0.545 |  | 1.25 | 1.02 | 1.52 | 0.033 |  | 1.24 | 1.03 | 1.49 | 0.027 |
|  | Wave (ref. Wave 9) |  |  |  | 0.009 |  |  |  |  | <0.001 |  |  |  |  | 0.120 |
|  | Wave 8 | 1.24 | 1.06 | 1.45 | 0.008 |  | 1.57 | 1.28 | 1.93 | <0.001 |  | 1.23 | 1.01 | 1.49 | 0.040 |
|  | Wave 10 | 0.99 | 0.83 | 1.17 | 0.873 |  | 1.21 | 0.97 | 1.52 | 0.097 |  | 1.09 | 0.88 | 1.34 | 0.435 |
|  | Wave (ref. Wave 9) * Gender (ref. Men) |  |  |  | 0.470 |  |  |  |  | 0.477 |  |  |  |  | 0.529 |
|  | Wave 8 * Women | 1.10 | 0.89 | 1.36 | 0.402 |  | 0.85 | 0.65 | 1.11 | 0.228 |  | 0.93 | 0.73 | 1.19 | 0.569 |
|  | Wave 10 * Women | 1.14 | 0.91 | 1.43 | 0.241 |  | 0.95 | 0.71 | 1.27 | 0.720 |  | 1.09 | 0.84 | 1.42 | 0.501 |
|  | Age group (ref. 50-59) |  |  |  |  |  |  |  |  |  |  |  |  |  |  |
|  | 60-69 | 0.76 | 0.66 | 0.87 | <0.001 |  | 0.67 | 0.57 | 0.80 | <0.001 |  | 0.68 | 0.58 | 0.80 | <0.001 |
|  | 70-79 | 0.70 | 0.61 | 0.80 | <0.001 |  | 0.59 | 0.49 | 0.70 | <0.001 |  | 0.54 | 0.46 | 0.64 | <0.001 |
|  | 80+ | 0.57 | 0.48 | 0.67 | <0.001 |  | 0.59 | 0.48 | 0.73 | <0.001 |  | 0.47 | 0.38 | 0.57 | <0.001 |
|  | Chronic conditions (ref. No illness) |  |  |  |  |  |  |  |  |  |  |  |  |  |  |
|  | Non-limiting illness | 1.16 | 1.02 | 1.32 | 0.020 |  | 1.17 | 0.99 | 1.37 | 0.060 |  | 1.27 | 1.07 | 1.50 | 0.005 |
|  | Limiting illness | 1.17 | 1.04 | 1.32 | 0.009 |  | 1.94 | 1.67 | 2.25 | <0.001 |  | 2.26 | 1.96 | 2.60 | <0.001 |
| By living situation | Living situation: Not living alone | 1.08 | 0.90 | 1.30 | 0.426 |  | 0.84 | 0.67 | 1.06 | 0.140 |  | 0.78 | 0.63 | 0.96 | 0.017 |
|  | Wave (ref. Wave 9) |  |  |  | 0.029 |  |  |  |  | 0.007 |  |  |  |  | 0.443 |
|  | Wave 8 | 1.31 | 1.06 | 1.63 | 0.015 |  | 1.46 | 1.15 | 1.86 | 0.002 |  | 1.14 | 0.91 | 1.42 | 0.249 |
|  | Wave 10 | 1.27 | 1.02 | 1.59 | 0.034 |  | 1.16 | 0.89 | 1.51 | 0.287 |  | 1.01 | 0.79 | 1.27 | 0.966 |
|  | Wave (ref. Wave 9) * Living situation (ref. Living alone) |  |  |  | 0.075 |  |  |  |  | 0.995 |  |  |  |  | 0.460 |
|  | Wave 8 * Not living alone | 1.00 | 0.78 | 1.29 | 0.984 |  | 0.99 | 0.74 | 1.31 | 0.932 |  | 1.06 | 0.82 | 1.38 | 0.658 |
|  | Wave 10 * Not living alone | 0.77 | 0.60 | 1.00 | 0.046 |  | 1.00 | 0.73 | 1.38 | 0.989 |  | 1.20 | 0.90 | 1.59 | 0.215 |
|  | Age group (ref. 50-59) |  |  |  |  |  |  |  |  |  |  |  |  |  |  |
|  | 60-69 | 0.77 | 0.66 | 0.88 | <0.001 |  | 0.64 | 0.53 | 0.76 | <0.001 |  | 0.67 | 0.57 | 0.80 | <0.001 |
|  | 70-79 | 0.74 | 0.64 | 0.85 | <0.001 |  | 0.55 | 0.46 | 0.66 | <0.001 |  | 0.53 | 0.45 | 0.63 | <0.001 |
|  | 80+ | 0.63 | 0.53 | 0.75 | <0.001 |  | 0.53 | 0.42 | 0.65 | <0.001 |  | 0.43 | 0.35 | 0.53 | <0.001 |
|  | Gender: Women | 1.07 | 0.96 | 1.19 | 0.221 |  | 1.16 | 1.01 | 1.33 | 0.038 |  | 1.29 | 1.12 | 1.47 | <0.001 |
|  | Highest educational qualification (ref. Post-secondary) |  |  |  |  |  |  |  |  |  |  |  |  |  |  |
|  | A-level, O-Level, CSE, technical equivalent | 0.71 | 0.62 | 0.80 | <0.001 |  | 0.94 | 0.79 | 1.10 | 0.430 |  | 0.73 | 0.62 | 0.86 | <0.001 |
|  | No academic qualification | 0.56 | 0.47 | 0.67 | <0.001 |  | 1.08 | 0.87 | 1.34 | 0.469 |  | 0.95 | 0.77 | 1.18 | 0.667 |
|  | Chronic conditions (ref. No illness) |  |  |  |  |  |  |  |  |  |  |  |  |  |  |
|  | Non-limiting illness | 1.16 | 1.02 | 1.32 | 0.024 |  | 1.17 | 1.00 | 1.38 | 0.055 |  | 1.21 | 1.03 | 1.42 | 0.017 |
|  | Limiting illness | 1.23 | 1.09 | 1.38 | 0.001 |  | 1.91 | 1.64 | 2.22 | <0.001 |  | 2.24 | 1.94 | 2.58 | <0.001 |
| By wealth quintile | Wealth quintile (ref. 1st, Poorest) |  |  |  | 0.088 |  |  |  |  | 0.062 |  |  |  |  | 0.205 |
|  | 2nd | 0.79 | 0.59 | 1.06 | 0.115 |  | 0.76 | 0.55 | 1.07 | 0.112 |  | 0.92 | 0.67 | 1.25 | 0.577 |
|  | 3rd | 0.83 | 0.62 | 1.10 | 0.184 |  | 0.66 | 0.48 | 0.91 | 0.012 |  | 0.76 | 0.56 | 1.02 | 0.069 |
|  | 4th | 0.96 | 0.72 | 1.27 | 0.773 |  | 0.70 | 0.51 | 0.97 | 0.034 |  | 0.81 | 0.59 | 1.11 | 0.194 |
|  | 5th, Wealthiest | 1.06 | 0.80 | 1.40 | 0.680 |  | 0.63 | 0.45 | 0.88 | 0.007 |  | 0.72 | 0.53 | 0.98 | 0.039 |
|  | Wave (ref. Wave 9) |  |  |  | 0.561 |  |  |  |  | 0.205 |  |  |  |  | 0.125 |
|  | Wave 8 | 1.13 | 0.81 | 1.58 | 0.459 |  | 1.35 | 0.96 | 1.90 | 0.088 |  | 1.36 | 1.01 | 1.83 | 0.041 |
|  | Wave 10 | 0.93 | 0.67 | 1.30 | 0.689 |  | 1.23 | 0.86 | 1.77 | 0.256 |  | 1.14 | 0.82 | 1.59 | 0.427 |
|  | Wave (ref. Wave 9) * Wealth quintile (ref. 1st, Poorest) |  |  |  | 0.289 |  |  |  |  | 0.145 |  |  |  |  | 0.945 |
|  | Wave 8 * 2nd | 1.27 | 0.83 | 1.95 | 0.267 |  | 1.12 | 0.69 | 1.82 | 0.643 |  | 0.86 | 0.57 | 1.30 | 0.464 |
|  | Wave 8 * 3rd | 1.36 | 0.90 | 2.05 | 0.145 |  | 1.51 | 0.98 | 2.35 | 0.064 |  | 0.94 | 0.62 | 1.43 | 0.772 |
|  | Wave 8 * 4th | 1.19 | 0.80 | 1.77 | 0.381 |  | 1.06 | 0.66 | 1.69 | 0.818 |  | 0.83 | 0.54 | 1.27 | 0.387 |
|  | Wave 8 * 5th, Wealthiest | 1.05 | 0.71 | 1.56 | 0.815 |  | 0.85 | 0.54 | 1.35 | 0.485 |  | 0.78 | 0.53 | 1.16 | 0.223 |
|  | Wave 10 * 2nd | 1.48 | 0.95 | 2.30 | 0.082 |  | 1.05 | 0.64 | 1.72 | 0.855 |  | 1.10 | 0.70 | 1.72 | 0.685 |
|  | Wave 10 * 3rd | 1.16 | 0.76 | 1.76 | 0.491 |  | 0.99 | 0.61 | 1.59 | 0.957 |  | 1.03 | 0.67 | 1.59 | 0.884 |
|  | Wave 10 * 4th | 1.05 | 0.70 | 1.58 | 0.805 |  | 0.88 | 0.55 | 1.41 | 0.602 |  | 0.91 | 0.59 | 1.40 | 0.672 |
|  | Wave 10 * 5th, Wealthiest | 0.92 | 0.61 | 1.37 | 0.667 |  | 0.63 | 0.39 | 1.03 | 0.066 |  | 0.94 | 0.61 | 1.44 | 0.772 |
|  | Age group (ref. 50-59) |  |  |  |  |  |  |  |  |  |  |  |  |  |  |
|  | 60-69 | 0.77 | 0.67 | 0.89 | <0.001 |  | 0.69 | 0.58 | 0.83 | <0.001 |  | 0.73 | 0.62 | 0.87 | <0.001 |
|  | 70-79 | 0.74 | 0.64 | 0.86 | <0.001 |  | 0.62 | 0.51 | 0.75 | <0.001 |  | 0.59 | 0.49 | 0.71 | <0.001 |
|  | 80+ | 0.63 | 0.53 | 0.76 | <0.001 |  | 0.62 | 0.49 | 0.77 | <0.001 |  | 0.50 | 0.40 | 0.62 | <0.001 |
|  | Gender: Women | 1.06 | 0.95 | 1.18 | 0.303 |  | 1.17 | 1.02 | 1.34 | 0.029 |  | 1.29 | 1.13 | 1.48 | <0.001 |
|  | Highest educational qualification (ref. Post-secondary) |  |  |  |  |  |  |  |  |  |  |  |  |  |  |
|  | A-level, O-Level, CSE, technical equivalent | 0.73 | 0.64 | 0.84 | <0.001 |  | 0.85 | 0.72 | 1.01 | 0.057 |  | 0.68 | 0.58 | 0.81 | <0.001 |
|  | No academic qualification | 0.59 | 0.50 | 0.71 | <0.001 |  | 0.91 | 0.72 | 1.13 | 0.383 |  | 0.85 | 0.68 | 1.07 | 0.174 |
|  | Chronic conditions (ref. No illness) |  |  |  |  |  |  |  |  |  |  |  |  |  |  |
|  | Non-limiting illness | 1.16 | 1.02 | 1.32 | 0.025 |  | 1.17 | 0.99 | 1.37 | 0.066 |  | 1.18 | 1.00 | 1.38 | 0.045 |
|  | Limiting illness | 1.23 | 1.09 | 1.39 | 0.001 |  | 1.77 | 1.52 | 2.06 | <0.001 |  | 2.11 | 1.82 | 2.44 | <0.001 |

Note. Weighted and adjusted models. LB: 95% confidence interval lower bound; ONS: Office for National Statistics; RRR: relative risk ratio; UB: 95% confidence interval upper bound. P-values for overall wave, wealth, and the interaction between wave and the grouping variable correspond to the omnibus test.

# Table S6. Results from the models stratified by age group. Weighted results. Age group: 50 to 69 year-olds.

|  |  | High depressive symptomatology  (CESD-8 ≥ 4) | | | |  | High anxiety  (ONS single question ≥ 6) | | | |  | High loneliness  (UCLA-3 ≥ 6) | | | |  | Quality of life  (CASP-19) | | | |  |
| --- | --- | --- | --- | --- | --- | --- | --- | --- | --- | --- | --- | --- | --- | --- | --- | --- | --- | --- | --- | --- | --- |
|  | Variable | RR | LB | UB | p-value |  | RR | LB | UB | p-value |  | RR | LB | UB | p-value |  | B | LB | UB | p-value | |
| Overall | Wave (ref. Wave 9) |  |  |  | 0.001 |  |  |  |  | 0.681 |  |  |  |  | <0.001 |  |  |  |  | <0.001 | |
|  | Wave 8 | 1.03 | 0.91 | 1.16 | 0.659 |  | 1.01 | 0.88 | 1.15 | 0.938 |  | 0.91 | 0.82 | 1.01 | 0.077 |  | -0.15 | -0.61 | 0.32 | 0.532 | |
|  | Wave 10 | 1.25 | 1.11 | 1.42 | <0.001 |  | 1.06 | 0.93 | 1.21 | 0.407 |  | 1.27 | 1.14 | 1.40 | <0.001 |  | -1.86 | -2.38 | -1.35 | <0.001 | |
|  | Age group (ref. 50-59) |  |  |  |  |  |  |  |  |  |  |  |  |  |  |  |  |  |  |  | |
|  | 60-69 | 0.70 | 0.62 | 0.78 | <0.001 |  | 0.83 | 0.74 | 0.93 | 0.002 |  | 0.83 | 0.75 | 0.93 | 0.001 |  | 2.12 | 1.57 | 2.68 | <0.001 | |
|  | Gender: Women | 1.28 | 1.11 | 1.48 | 0.001 |  | 1.14 | 1.01 | 1.30 | 0.041 |  | 1.04 | 0.93 | 1.17 | 0.484 |  | 0.79 | 0.18 | 1.39 | 0.010 | |
|  | Highest educational qualification (ref. Post-secondary) |  |  |  |  |  |  |  |  |  |  |  |  |  |  |  |  |  |  |  | |
|  | A-level, O-Level, CSE, technical equivalent | 1.44 | 1.20 | 1.73 | <0.001 |  | 0.84 | 0.73 | 0.97 | 0.015 |  | 1.35 | 1.15 | 1.57 | <0.001 |  | -1.92 | -2.53 | -1.31 | <0.001 | |
|  | No academic qualification | 2.08 | 1.69 | 2.56 | <0.001 |  | 1.12 | 0.90 | 1.37 | 0.307 |  | 1.68 | 1.38 | 2.04 | <0.001 |  | -4.39 | -5.44 | -3.34 | <0.001 | |
|  | Chronic conditions (ref. No illness) |  |  |  |  |  |  |  |  |  |  |  |  |  |  |  |  |  |  |  | |
|  | Non-limiting illness | 1.65 | 1.32 | 2.05 | <0.001 |  | 1.09 | 0.92 | 1.28 | 0.328 |  | 1.24 | 1.07 | 1.44 | 0.005 |  | -1.88 | -2.52 | -1.24 | <0.001 | |
|  | Limiting illness | 5.20 | 4.38 | 6.18 | <0.001 |  | 1.78 | 1.55 | 2.05 | <0.001 |  | 2.25 | 2.00 | 2.54 | <0.001 |  | -9.64 | -10.35 | -8.93 | <0.001 | |
| By gender | Gender: Women | 1.43 | 1.16 | 1.75 | 0.001 |  | 1.11 | 0.92 | 1.35 | 0.268 |  | 1.09 | 0.92 | 1.28 | 0.330 |  | 0.80 | 0.03 | 1.56 | 0.042 | |
|  | Wave (ref. Wave 9) |  |  |  | 0.003 |  |  |  |  | 0.845 |  |  |  |  | 0.001 |  |  |  |  | <0.001 | |
|  | Wave 8 | 1.03 | 0.83 | 1.28 | 0.791 |  | 1.06 | 0.86 | 1.30 | 0.583 |  | 0.96 | 0.82 | 1.13 | 0.654 |  | -0.34 | -1.10 | 0.42 | 0.382 | |
|  | Wave 10 | 1.45 | 1.16 | 1.81 | 0.001 |  | 1.00 | 0.81 | 1.25 | 0.977 |  | 1.34 | 1.13 | 1.58 | 0.001 |  | -1.92 | -2.71 | -1.14 | <0.001 | |
|  | Wave (ref. Wave 9) * Gender (ref. Men) |  |  |  | 0.157 |  |  |  |  | 0.447 |  |  |  |  | 0.527 |  |  |  |  | 0.970 | |
|  | Wave 8 * Women | 1.00 | 0.77 | 1.30 | 0.997 |  | 0.89 | 0.69 | 1.16 | 0.389 |  | 0.91 | 0.74 | 1.12 | 0.359 |  | 0.12 | -0.82 | 1.06 | 0.809 | |
|  | Wave 10 * Women | 0.79 | 0.60 | 1.03 | 0.079 |  | 1.07 | 0.82 | 1.41 | 0.608 |  | 0.90 | 0.73 | 1.12 | 0.348 |  | 0.06 | -0.98 | 1.10 | 0.912 | |
|  | Age group (ref. 50-59) |  |  |  |  |  |  |  |  |  |  |  |  |  |  |  |  |  |  |  | |
|  | 60-69 | 0.72 | 0.64 | 0.80 | <0.001 |  | 0.83 | 0.74 | 0.94 | 0.003 |  | 0.85 | 0.76 | 0.94 | 0.002 |  | 1.90 | 1.32 | 2.48 | <0.001 | |
|  | Chronic conditions (ref. No illness) |  |  |  |  |  |  |  |  |  |  |  |  |  |  |  |  |  |  |  | |
|  | Non-limiting illness | 1.74 | 1.40 | 2.18 | <0.001 |  | 1.15 | 0.96 | 1.37 | 0.126 |  | 1.24 | 1.06 | 1.45 | 0.008 |  | -1.95 | -2.71 | -1.19 | <0.001 | |
|  | Limiting illness | 5.76 | 4.87 | 6.82 | <0.001 |  | 1.79 | 1.56 | 2.05 | <0.001 |  | 2.32 | 2.06 | 2.61 | <0.001 |  | -9.84 | -10.56 | -9.12 | <0.001 | |
| By living situation | Living situation: Not living alone | 0.56 | 0.46 | 0.68 | <0.001 |  | 0.78 | 0.63 | 0.97 | 0.022 |  | 0.50 | 0.42 | 0.59 | <0.001 |  | 2.43 | 1.43 | 3.44 | <0.001 | |
|  | Wave (ref. Wave 9) |  |  |  | 0.681 |  |  |  |  | 0.416 |  |  |  |  | 0.003 |  |  |  |  | 0.002 | |
|  | Wave 8 | 1.06 | 0.88 | 1.28 | 0.538 |  | 1.00 | 0.77 | 1.28 | 0.971 |  | 0.91 | 0.78 | 1.05 | 0.186 |  | -0.09 | -1.22 | 1.03 | 0.870 | |
|  | Wave 10 | 1.08 | 0.90 | 1.30 | 0.399 |  | 0.85 | 0.66 | 1.11 | 0.230 |  | 1.19 | 1.03 | 1.38 | 0.019 |  | -2.15 | -3.41 | -0.89 | 0.001 | |
|  | Wave (ref. Wave 9) * Living situation (ref. Living alone) |  |  |  | 0.113 |  |  |  |  | 0.164 |  |  |  |  | 0.639 |  |  |  |  | 0.829 | |
|  | Wave 8 * Not living alone | 0.97 | 0.76 | 1.25 | 0.825 |  | 1.02 | 0.76 | 1.36 | 0.908 |  | 1.02 | 0.83 | 1.25 | 0.846 |  | -0.09 | -1.34 | 1.16 | 0.887 | |
|  | Wave 10 * Not living alone | 1.25 | 0.97 | 1.59 | 0.080 |  | 1.31 | 0.97 | 1.78 | 0.079 |  | 1.10 | 0.90 | 1.34 | 0.354 |  | 0.36 | -1.04 | 1.75 | 0.615 | |
|  | Age group (ref. 50-59) |  |  |  |  |  |  |  |  |  |  |  |  |  |  |  |  |  |  |  | |
|  | 60-69 | 0.69 | 0.61 | 0.77 | <0.001 |  | 0.83 | 0.73 | 0.93 | 0.002 |  | 0.81 | 0.73 | 0.90 | <0.001 |  | 2.22 | 1.67 | 2.77 | <0.001 | |
|  | Gender: Women | 1.32 | 1.14 | 1.52 | <0.001 |  | 1.15 | 1.01 | 1.30 | 0.040 |  | 1.07 | 0.95 | 1.19 | 0.278 |  | 0.77 | 0.17 | 1.37 | 0.012 | |
|  | Highest educational qualification (ref. Post-secondary) |  |  |  |  |  |  |  |  |  |  |  |  |  |  |  |  |  |  |  | |
|  | A-level, O-Level, CSE, technical equivalent | 1.42 | 1.18 | 1.70 | <0.001 |  | 0.84 | 0.72 | 0.96 | 0.014 |  | 1.33 | 1.14 | 1.55 | <0.001 |  | -1.90 | -2.51 | -1.28 | <0.001 | |
|  | No academic qualification | 2.01 | 1.63 | 2.47 | <0.001 |  | 1.11 | 0.90 | 1.37 | 0.307 |  | 1.63 | 1.34 | 1.98 | <0.001 |  | -4.33 | -5.38 | -3.29 | <0.001 | |
|  | Chronic conditions (ref. No illness) |  |  |  |  |  |  |  |  |  |  |  |  |  |  |  |  |  |  |  | |
|  | Non-limiting illness | 1.62 | 1.30 | 2.01 | <0.001 |  | 1.08 | 0.92 | 1.28 | 0.346 |  | 1.21 | 1.05 | 1.41 | 0.010 |  | -1.83 | -2.47 | -1.19 | <0.001 | |
|  | Limiting illness | 4.82 | 4.05 | 5.73 | <0.001 |  | 1.74 | 1.51 | 2.00 | <0.001 |  | 2.05 | 1.81 | 2.31 | <0.001 |  | -9.38 | -10.09 | -8.66 | <0.001 | |
| By wealth quintile | Wealth quintile (ref. 1st, Poorest) |  |  |  | <0.001 |  |  |  |  | 0.220 |  |  |  |  | <0.001 |  |  |  |  | <0.001 | |
|  | 2nd | 0.72 | 0.54 | 0.95 | 0.021 |  | 1.14 | 0.85 | 1.51 | 0.381 |  | 0.99 | 0.78 | 1.25 | 0.926 |  | 2.19 | 0.83 | 3.56 | 0.002 | |
|  | 3rd | 0.53 | 0.39 | 0.71 | <0.001 |  | 0.90 | 0.67 | 1.20 | 0.455 |  | 0.73 | 0.57 | 0.93 | 0.012 |  | 3.99 | 2.77 | 5.20 | <0.001 | |
|  | 4th | 0.48 | 0.34 | 0.67 | <0.001 |  | 0.88 | 0.64 | 1.21 | 0.432 |  | 0.69 | 0.53 | 0.88 | 0.004 |  | 4.39 | 3.16 | 5.62 | <0.001 | |
|  | 5th, Wealthiest | 0.28 | 0.19 | 0.42 | <0.001 |  | 0.79 | 0.58 | 1.07 | 0.130 |  | 0.58 | 0.43 | 0.77 | <0.001 |  | 5.76 | 4.68 | 6.85 | <0.001 | |
|  | Wave (ref. Wave 9) |  |  |  | 0.138 |  |  |  |  | 0.374 |  |  |  |  | 0.014 |  |  |  |  | 0.001 | |
|  | Wave 8 | 1.15 | 0.96 | 1.36 | 0.123 |  | 1.19 | 0.93 | 1.52 | 0.163 |  | 1.10 | 0.91 | 1.33 | 0.319 |  | -1.09 | -2.37 | 0.20 | 0.097 | |
|  | Wave 10 | 1.18 | 0.98 | 1.41 | 0.078 |  | 1.10 | 0.85 | 1.43 | 0.477 |  | 1.31 | 1.09 | 1.58 | 0.004 |  | -2.50 | -3.83 | -1.18 | <0.001 | |
|  | Wave (ref. Wave 9) * Wealth quintile (ref. 1st, Poorest) |  |  |  | 0.600 |  |  |  |  | 0.733 |  |  |  |  | 0.575 |  |  |  |  | 0.488 | |
|  | Wave 8 * 2nd | 0.87 | 0.59 | 1.28 | 0.487 |  | 0.77 | 0.52 | 1.15 | 0.209 |  | 0.77 | 0.54 | 1.11 | 0.165 |  | 1.13 | -0.92 | 3.18 | 0.281 | |
|  | Wave 8 * 3rd | 0.83 | 0.54 | 1.26 | 0.379 |  | 0.78 | 0.50 | 1.22 | 0.275 |  | 0.78 | 0.55 | 1.11 | 0.170 |  | 0.89 | -0.89 | 2.67 | 0.325 | |
|  | Wave 8 * 4th | 0.66 | 0.41 | 1.06 | 0.088 |  | 0.88 | 0.58 | 1.33 | 0.532 |  | 0.74 | 0.53 | 1.02 | 0.068 |  | 1.48 | -0.11 | 3.07 | 0.068 | |
|  | Wave 8 * 5th, Wealthiest | 1.06 | 0.63 | 1.77 | 0.838 |  | 0.79 | 0.54 | 1.16 | 0.226 |  | 0.73 | 0.51 | 1.04 | 0.083 |  | 0.99 | -0.51 | 2.50 | 0.195 | |
|  | Wave 10 * 2nd | 1.09 | 0.74 | 1.59 | 0.664 |  | 0.88 | 0.58 | 1.31 | 0.522 |  | 0.93 | 0.68 | 1.27 | 0.647 |  | 0.18 | -1.83 | 2.18 | 0.862 | |
|  | Wave 10 * 3rd | 1.13 | 0.75 | 1.72 | 0.559 |  | 1.02 | 0.67 | 1.55 | 0.921 |  | 1.02 | 0.74 | 1.40 | 0.918 |  | 0.85 | -0.93 | 2.63 | 0.349 | |
|  | Wave 10 * 4th | 1.05 | 0.66 | 1.65 | 0.845 |  | 0.80 | 0.52 | 1.23 | 0.312 |  | 1.01 | 0.73 | 1.40 | 0.930 |  | 1.87 | 0.07 | 3.67 | 0.041 | |
|  | Wave 10 * 5th, Wealthiest | 1.35 | 0.81 | 2.26 | 0.249 |  | 1.02 | 0.69 | 1.53 | 0.906 |  | 0.93 | 0.67 | 1.31 | 0.692 |  | 1.00 | -0.61 | 2.60 | 0.223 | |
|  | Age group (ref. 50-59) |  |  |  |  |  |  |  |  |  |  |  |  |  |  |  |  |  |  |  | |
|  | 60-69 | 0.81 | 0.72 | 0.91 | <0.001 |  | 0.88 | 0.77 | 1.00 | 0.046 |  | 0.91 | 0.82 | 1.01 | 0.088 |  | 1.12 | 0.59 | 1.66 | <0.001 | |
|  | Gender: Women | 1.28 | 1.11 | 1.47 | 0.001 |  | 1.15 | 1.00 | 1.31 | 0.043 |  | 1.05 | 0.93 | 1.18 | 0.422 |  | 0.82 | 0.24 | 1.41 | 0.006 | |
|  | Highest educational qualification (ref. Post-secondary) |  |  |  |  |  |  |  |  |  |  |  |  |  |  |  |  |  |  |  | |
|  | A-level, O-Level, CSE, technical equivalent | 1.17 | 0.97 | 1.41 | 0.095 |  | 0.79 | 0.68 | 0.91 | 0.001 |  | 1.18 | 1.01 | 1.39 | 0.041 |  | -0.79 | -1.38 | -0.20 | 0.009 | |
|  | No academic qualification | 1.43 | 1.15 | 1.76 | 0.001 |  | 1.01 | 0.80 | 1.26 | 0.943 |  | 1.30 | 1.06 | 1.59 | 0.013 |  | -1.99 | -3.02 | -0.97 | <0.001 | |
|  | Chronic conditions (ref. No illness) |  |  |  |  |  |  |  |  |  |  |  |  |  |  |  |  |  |  |  | |
|  | Non-limiting illness | 1.51 | 1.21 | 1.89 | <0.001 |  | 1.05 | 0.88 | 1.24 | 0.589 |  | 1.21 | 1.04 | 1.41 | 0.014 |  | -1.47 | -2.10 | -0.84 | <0.001 | |
|  | Limiting illness | 4.25 | 3.56 | 5.08 | <0.001 |  | 1.68 | 1.45 | 1.94 | <0.001 |  | 2.04 | 1.80 | 2.31 | <0.001 |  | -8.45 | -9.16 | -7.75 | <0.001 | |

Note. Weighted and adjusted models. B: regression coefficient; CASP-19: 19-item Control, Autonomy, Self-realisation and Pleasure; CESD-8: 8-item Centre for Epidemiologic Studies Depression Scale; LB: 95% confidence interval lower bound; ONS: Office for National Statistics; RR: risk ratio; UB: 95% confidence interval upper bound; UCLA-3: University of California Los Angeles 3-item loneliness scale. P-values for overall wave, wealth, and the interaction between wave and the grouping variable correspond to the omnibus test.

# Table S7. Results from the models stratified by age group. Weighted results. Age group: 70+ year-olds.

|  |  | High depressive symptomatology  (CESD-8 ≥ 4) | | | |  | High anxiety  (ONS single question ≥ 6) | | | |  | High loneliness  (UCLA-3 ≥ 6) | | | |  | Quality of life  (CASP-19) | | | |  |
| --- | --- | --- | --- | --- | --- | --- | --- | --- | --- | --- | --- | --- | --- | --- | --- | --- | --- | --- | --- | --- | --- |
|  | Variable | RR | LB | UB | p-value |  | RR | LB | UB | p-value |  | RR | LB | UB | p-value |  | B | LB | UB | p-value | |
| Overall | Wave (ref. Wave 9) |  |  |  | 0.067 |  |  |  |  | 0.149 |  |  |  |  | <0.001 |  |  |  |  | <0.001 | |
|  | Wave 8 | 1.01 | 0.91 | 1.13 | 0.797 |  | 1.05 | 0.93 | 1.18 | 0.429 |  | 1.05 | 0.96 | 1.16 | 0.272 |  | -0.53 | -0.88 | -0.19 | 0.003 | |
|  | Wave 10 | 1.15 | 1.02 | 1.29 | 0.026 |  | 1.14 | 1.00 | 1.29 | 0.051 |  | 1.41 | 1.29 | 1.55 | <0.001 |  | -1.65 | -2.07 | -1.23 | <0.001 | |
|  | Age group (ref. 70-79) |  |  |  |  |  |  |  |  |  |  |  |  |  |  |  |  |  |  |  | |
|  | 80+ | 1.24 | 1.09 | 1.40 | 0.001 |  | 0.92 | 0.81 | 1.05 | 0.236 |  | 1.19 | 1.07 | 1.32 | 0.001 |  | -2.10 | -2.60 | -1.59 | <0.001 | |
|  | Gender: Women | 1.61 | 1.40 | 1.85 | <0.001 |  | 1.30 | 1.15 | 1.47 | <0.001 |  | 1.26 | 1.13 | 1.41 | <0.001 |  | 0.30 | -0.20 | 0.79 | 0.239 | |
|  | Highest educational qualification (ref. Post-secondary) |  |  |  |  |  |  |  |  |  |  |  |  |  |  |  |  |  |  |  | |
|  | A-level, O-Level, CSE, technical equivalent | 1.27 | 1.04 | 1.55 | 0.018 |  | 0.90 | 0.77 | 1.06 | 0.228 |  | 1.21 | 1.04 | 1.41 | 0.016 |  | -1.28 | -1.90 | -0.67 | <0.001 | |
|  | No academic qualification | 1.47 | 1.18 | 1.82 | <0.001 |  | 1.06 | 0.88 | 1.28 | 0.553 |  | 1.36 | 1.14 | 1.63 | 0.001 |  | -2.12 | -2.88 | -1.36 | <0.001 | |
|  | Chronic conditions (ref. No illness) |  |  |  |  |  |  |  |  |  |  |  |  |  |  |  |  |  |  |  | |
|  | Non-limiting illness | 1.15 | 0.93 | 1.42 | 0.193 |  | 1.13 | 0.96 | 1.34 | 0.130 |  | 1.26 | 1.10 | 1.45 | 0.001 |  | -1.43 | -1.98 | -0.88 | <0.001 | |
|  | Limiting illness | 2.99 | 2.55 | 3.51 | <0.001 |  | 1.47 | 1.28 | 1.68 | <0.001 |  | 1.57 | 1.39 | 1.78 | <0.001 |  | -6.58 | -7.10 | -6.06 | <0.001 | |
| By gender | Gender: Women | 2.21 | 1.77 | 2.74 | <0.001 |  | 1.32 | 1.09 | 1.60 | 0.005 |  | 1.42 | 1.20 | 1.67 | <0.001 |  | -0.15 | -0.77 | 0.47 | 0.630 | |
|  | Wave (ref. Wave 9) |  |  |  | <0.001 |  |  |  |  | 0.342 |  |  |  |  | <0.001 |  |  |  |  | <0.001 | |
|  | Wave 8 | 1.28 | 1.02 | 1.62 | 0.037 |  | 1.04 | 0.85 | 1.27 | 0.724 |  | 1.11 | 0.94 | 1.30 | 0.219 |  | -0.78 | -1.29 | -0.28 | 0.002 | |
|  | Wave 10 | 1.61 | 1.27 | 2.04 | <0.001 |  | 1.17 | 0.94 | 1.45 | 0.154 |  | 1.55 | 1.32 | 1.82 | <0.001 |  | -1.77 | -2.35 | -1.19 | <0.001 | |
|  | Wave (ref. Wave 9) * Gender (ref. Men) |  |  |  | 0.001 |  |  |  |  | 0.811 |  |  |  |  | 0.243 |  |  |  |  | 0.552 | |
|  | Wave 8 * Women | 0.73 | 0.56 | 0.96 | 0.022 |  | 1.04 | 0.81 | 1.34 | 0.770 |  | 0.94 | 0.77 | 1.14 | 0.543 |  | 0.32 | -0.38 | 1.01 | 0.372 | |
|  | Wave 10 * Women | 0.60 | 0.46 | 0.79 | <0.001 |  | 0.95 | 0.73 | 1.24 | 0.700 |  | 0.85 | 0.69 | 1.03 | 0.095 |  | 0.37 | -0.47 | 1.20 | 0.389 | |
|  | Age group (ref. 70-79) |  |  |  |  |  |  |  |  |  |  |  |  |  |  |  |  |  |  |  | |
|  | 80+ | 1.28 | 1.13 | 1.45 | <0.001 |  | 0.94 | 0.82 | 1.07 | 0.338 |  | 1.23 | 1.10 | 1.36 | <0.001 |  | -2.29 | -2.79 | -1.78 | <0.001 | |
|  | Chronic conditions (ref. No illness) |  |  |  |  |  |  |  |  |  |  |  |  |  |  |  |  |  |  |  | |
|  | Non-limiting illness | 1.15 | 0.93 | 1.42 | 0.193 |  | 1.13 | 0.96 | 1.34 | 0.129 |  | 1.26 | 1.10 | 1.45 | 0.001 |  | -1.41 | -1.96 | -0.86 | <0.001 | |
|  | Limiting illness | 3.04 | 2.59 | 3.56 | <0.001 |  | 1.48 | 1.29 | 1.69 | <0.001 |  | 1.59 | 1.41 | 1.80 | <0.001 |  | -6.66 | -7.18 | -6.14 | <0.001 | |
| By living situation | Living situation: Not living alone | 0.55 | 0.46 | 0.66 | <0.001 |  | 0.92 | 0.75 | 1.12 | 0.396 |  | 0.47 | 0.40 | 0.55 | <0.001 |  | 0.75 | 0.05 | 1.45 | 0.035 | |
|  | Wave (ref. Wave 9) |  |  |  | 0.666 |  |  |  |  | 0.897 |  |  |  |  | <0.001 |  |  |  |  | <0.001 | |
|  | Wave 8 | 1.00 | 0.87 | 1.15 | 0.985 |  | 0.98 | 0.80 | 1.21 | 0.878 |  | 1.02 | 0.91 | 1.15 | 0.710 |  | -0.65 | -1.31 | 0.01 | 0.053 | |
|  | Wave 10 | 1.07 | 0.91 | 1.26 | 0.418 |  | 1.04 | 0.83 | 1.30 | 0.745 |  | 1.37 | 1.22 | 1.55 | <0.001 |  | -1.86 | -2.70 | -1.02 | <0.001 | |
|  | Wave (ref. Wave 9) * Living situation (ref. Living alone) |  |  |  | 0.446 |  |  |  |  | 0.588 |  |  |  |  | 0.844 |  |  |  |  | 0.782 | |
|  | Wave 8 * Not living alone | 1.01 | 0.80 | 1.26 | 0.959 |  | 1.10 | 0.86 | 1.42 | 0.447 |  | 1.01 | 0.84 | 1.23 | 0.882 |  | 0.21 | -0.59 | 1.01 | 0.602 | |
|  | Wave 10 * Not living alone | 1.16 | 0.91 | 1.48 | 0.239 |  | 1.15 | 0.87 | 1.51 | 0.336 |  | 1.06 | 0.87 | 1.27 | 0.570 |  | 0.31 | -0.67 | 1.29 | 0.541 | |
|  | Age group (ref. 70-79) |  |  |  |  |  |  |  |  |  |  |  |  |  |  |  |  |  |  |  | |
|  | 80+ | 1.10 | 0.97 | 1.24 | 0.149 |  | 0.92 | 0.80 | 1.06 | 0.235 |  | 1.02 | 0.91 | 1.13 | 0.764 |  | -1.92 | -2.43 | -1.41 | <0.001 | |
|  | Gender: Women | 1.44 | 1.25 | 1.66 | <0.001 |  | 1.30 | 1.14 | 1.47 | <0.001 |  | 1.09 | 0.97 | 1.22 | 0.136 |  | 0.45 | -0.05 | 0.95 | 0.075 | |
|  | Highest educational qualification (ref. Post-secondary) |  |  |  |  |  |  |  |  |  |  |  |  |  |  |  |  |  |  |  | |
|  | A-level, O-Level, CSE, technical equivalent | 1.28 | 1.05 | 1.56 | 0.016 |  | 0.91 | 0.77 | 1.07 | 0.232 |  | 1.20 | 1.03 | 1.40 | 0.018 |  | -1.27 | -1.88 | -0.66 | <0.001 | |
|  | No academic qualification | 1.43 | 1.16 | 1.77 | 0.001 |  | 1.06 | 0.87 | 1.28 | 0.555 |  | 1.32 | 1.11 | 1.57 | 0.001 |  | -2.08 | -2.84 | -1.32 | <0.001 | |
|  | Chronic conditions (ref. No illness) |  |  |  |  |  |  |  |  |  |  |  |  |  |  |  |  |  |  |  | |
|  | Non-limiting illness | 1.13 | 0.92 | 1.40 | 0.248 |  | 1.14 | 0.96 | 1.34 | 0.126 |  | 1.23 | 1.08 | 1.41 | 0.003 |  | -1.41 | -1.95 | -0.86 | <0.001 | |
|  | Limiting illness | 2.91 | 2.48 | 3.41 | <0.001 |  | 1.47 | 1.29 | 1.68 | <0.001 |  | 1.52 | 1.35 | 1.71 | <0.001 |  | -6.54 | -7.06 | -6.02 | <0.001 | |
| By wealth quintile | Wealth quintile (ref. 1st, Poorest) |  |  |  | <0.001 |  |  |  |  | 0.835 |  |  |  |  | <0.001 |  |  |  |  | <0.001 | |
|  | 2nd | 0.72 | 0.56 | 0.93 | 0.012 |  | 0.88 | 0.62 | 1.23 | 0.448 |  | 0.78 | 0.61 | 1.01 | 0.055 |  | 2.10 | 0.72 | 3.49 | 0.003 | |
|  | 3rd | 0.58 | 0.44 | 0.77 | <0.001 |  | 0.88 | 0.63 | 1.23 | 0.459 |  | 0.78 | 0.61 | 1.01 | 0.056 |  | 2.64 | 1.29 | 4.00 | <0.001 | |
|  | 4th | 0.48 | 0.35 | 0.65 | <0.001 |  | 0.93 | 0.67 | 1.30 | 0.676 |  | 0.57 | 0.43 | 0.75 | <0.001 |  | 4.28 | 2.95 | 5.61 | <0.001 | |
|  | 5th, Wealthiest | 0.52 | 0.37 | 0.72 | <0.001 |  | 0.82 | 0.58 | 1.17 | 0.282 |  | 0.50 | 0.37 | 0.67 | <0.001 |  | 4.78 | 3.44 | 6.13 | <0.001 | |
|  | Wave (ref. Wave 9) |  |  |  | 0.843 |  |  |  |  | 0.831 |  |  |  |  | 0.013 |  |  |  |  | 0.694 | |
|  | Wave 8 | 1.05 | 0.82 | 1.34 | 0.697 |  | 1.08 | 0.75 | 1.54 | 0.681 |  | 0.81 | 0.62 | 1.05 | 0.117 |  | -0.64 | -2.11 | 0.83 | 0.393 | |
|  | Wave 10 | 1.09 | 0.82 | 1.45 | 0.570 |  | 0.95 | 0.62 | 1.47 | 0.833 |  | 1.25 | 0.96 | 1.64 | 0.099 |  | -0.33 | -2.04 | 1.37 | 0.703 | |
|  | Wave (ref. Wave 9) * Wealth quintile (ref. 1st, Poorest) |  |  |  | 0.073 |  |  |  |  | 0.673 |  |  |  |  | 0.039 |  |  |  |  | 0.411 | |
|  | Wave 8 * 2nd | 0.92 | 0.67 | 1.28 | 0.635 |  | 1.02 | 0.65 | 1.60 | 0.936 |  | 1.35 | 0.97 | 1.88 | 0.073 |  | -0.19 | -1.98 | 1.60 | 0.837 | |
|  | Wave 8 * 3rd | 1.04 | 0.73 | 1.48 | 0.834 |  | 1.00 | 0.64 | 1.56 | 0.983 |  | 1.20 | 0.86 | 1.68 | 0.285 |  | 0.52 | -1.15 | 2.19 | 0.540 | |
|  | Wave 8 * 4th | 1.08 | 0.71 | 1.63 | 0.725 |  | 0.79 | 0.50 | 1.25 | 0.321 |  | 1.42 | 1.00 | 2.02 | 0.053 |  | 0.01 | -1.66 | 1.68 | 0.991 | |
|  | Wave 8 * 5th, Wealthiest | 0.75 | 0.49 | 1.17 | 0.208 |  | 1.02 | 0.65 | 1.62 | 0.923 |  | 1.65 | 1.14 | 2.39 | 0.008 |  | 0.48 | -1.16 | 2.12 | 0.566 | |
|  | Wave 10 * 2nd | 0.86 | 0.58 | 1.28 | 0.451 |  | 1.32 | 0.79 | 2.23 | 0.291 |  | 0.96 | 0.67 | 1.37 | 0.827 |  | -1.17 | -3.25 | 0.92 | 0.272 | |
|  | Wave 10 * 3rd | 1.31 | 0.90 | 1.91 | 0.164 |  | 1.10 | 0.66 | 1.83 | 0.706 |  | 1.21 | 0.87 | 1.68 | 0.254 |  | -1.72 | -3.72 | 0.28 | 0.091 | |
|  | Wave 10 * 4th | 1.47 | 0.98 | 2.22 | 0.064 |  | 1.27 | 0.78 | 2.09 | 0.338 |  | 1.38 | 0.97 | 1.95 | 0.070 |  | -1.75 | -3.64 | 0.14 | 0.070 | |
|  | Wave 10 * 5th, Wealthiest | 0.75 | 0.46 | 1.20 | 0.228 |  | 1.21 | 0.73 | 2.02 | 0.460 |  | 1.15 | 0.79 | 1.67 | 0.471 |  | -1.27 | -3.15 | 0.62 | 0.189 | |
|  | Age group (ref. 70-79) |  |  |  |  |  |  |  |  |  |  |  |  |  |  |  |  |  |  |  | |
|  | 80+ | 1.26 | 1.11 | 1.42 | <0.001 |  | 0.93 | 0.81 | 1.06 | 0.259 |  | 1.19 | 1.07 | 1.32 | 0.002 |  | -2.06 | -2.57 | -1.56 | <0.001 | |
|  | Gender: Women | 1.57 | 1.37 | 1.81 | <0.001 |  | 1.30 | 1.14 | 1.47 | <0.001 |  | 1.25 | 1.11 | 1.40 | <0.001 |  | 0.41 | -0.08 | 0.89 | 0.104 | |
|  | Highest educational qualification (ref. Post-secondary) |  |  |  |  |  |  |  |  |  |  |  |  |  |  |  |  |  |  |  | |
|  | A-level, O-Level, CSE, technical equivalent | 1.13 | 0.92 | 1.37 | 0.242 |  | 0.89 | 0.75 | 1.05 | 0.170 |  | 1.10 | 0.94 | 1.28 | 0.248 |  | -0.50 | -1.13 | 0.12 | 0.116 | |
|  | No academic qualification | 1.21 | 0.97 | 1.51 | 0.087 |  | 1.03 | 0.84 | 1.27 | 0.775 |  | 1.19 | 0.99 | 1.42 | 0.063 |  | -0.83 | -1.64 | -0.03 | 0.042 | |
|  | Chronic conditions (ref. No illness) |  |  |  |  |  |  |  |  |  |  |  |  |  |  |  |  |  |  |  | |
|  | Non-limiting illness | 1.12 | 0.91 | 1.39 | 0.283 |  | 1.14 | 0.97 | 1.34 | 0.116 |  | 1.26 | 1.09 | 1.44 | 0.001 |  | -1.35 | -1.89 | -0.81 | <0.001 | |
|  | Limiting illness | 2.73 | 2.33 | 3.20 | <0.001 |  | 1.45 | 1.27 | 1.66 | <0.001 |  | 1.50 | 1.33 | 1.70 | <0.001 |  | -6.10 | -6.62 | -5.59 | <0.001 | |

Note. Weighted and adjusted models. B: regression coefficient; CASP-19: 19-item Control, Autonomy, Self-realisation and Pleasure; CESD-8: 8-item Centre for Epidemiologic Studies Depression Scale; LB: 95% confidence interval lower bound; ONS: Office for National Statistics; RR: risk ratio; UB: 95% confidence interval upper bound; UCLA-3: University of California Los Angeles 3-item loneliness scale. P-values for overall wave, wealth, and the interaction between wave and the grouping variable correspond to the omnibus test.

# Figure S1. Marginal predicted probability of mental (ill-)health and predicted mean quality of life across time-points (brackets represent 95% confidence intervals) after excluding participants interviewed during the temporary restrictions between November 2021 and February 2022, using a non-face-to-face mode, or without a COVID-19 booster vaccine.


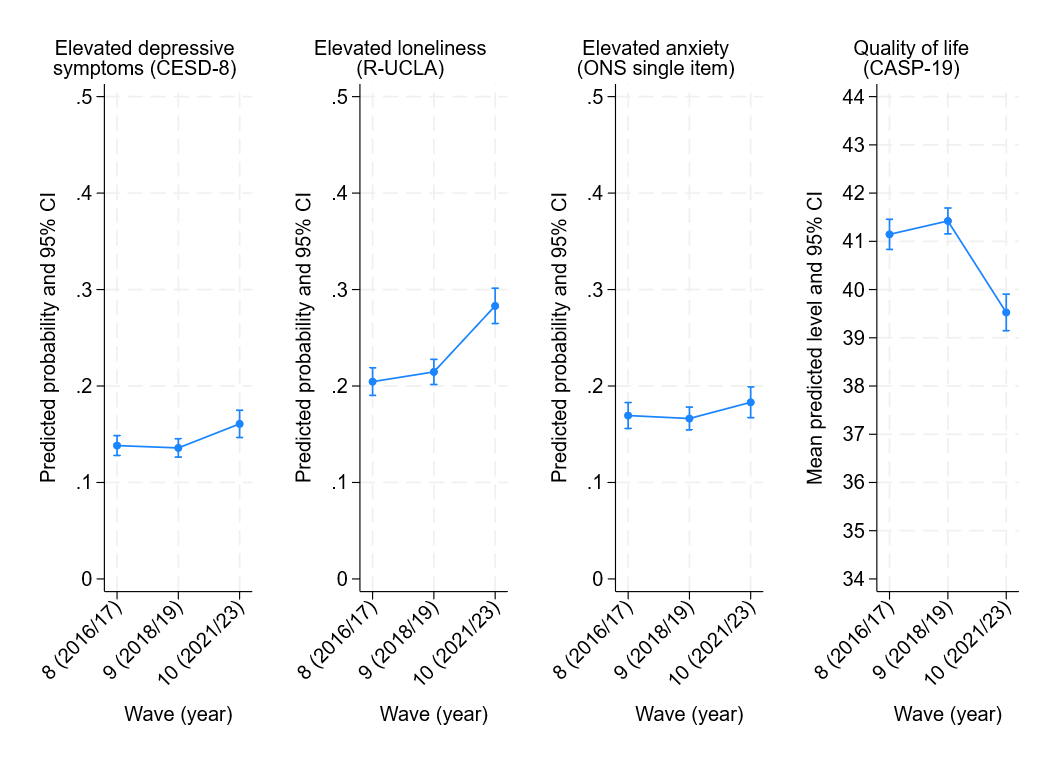


# Figure S2. Marginal predicted probability of mental (ill-)health and predicted mean quality of life across time-points (brackets represent 95% confidence intervals) by gender (women / men) after excluding participants interviewed during the temporary restrictions between November 2021 and February 2022, using a non-face-to-face mode, or without a COVID-19 booster vaccine.


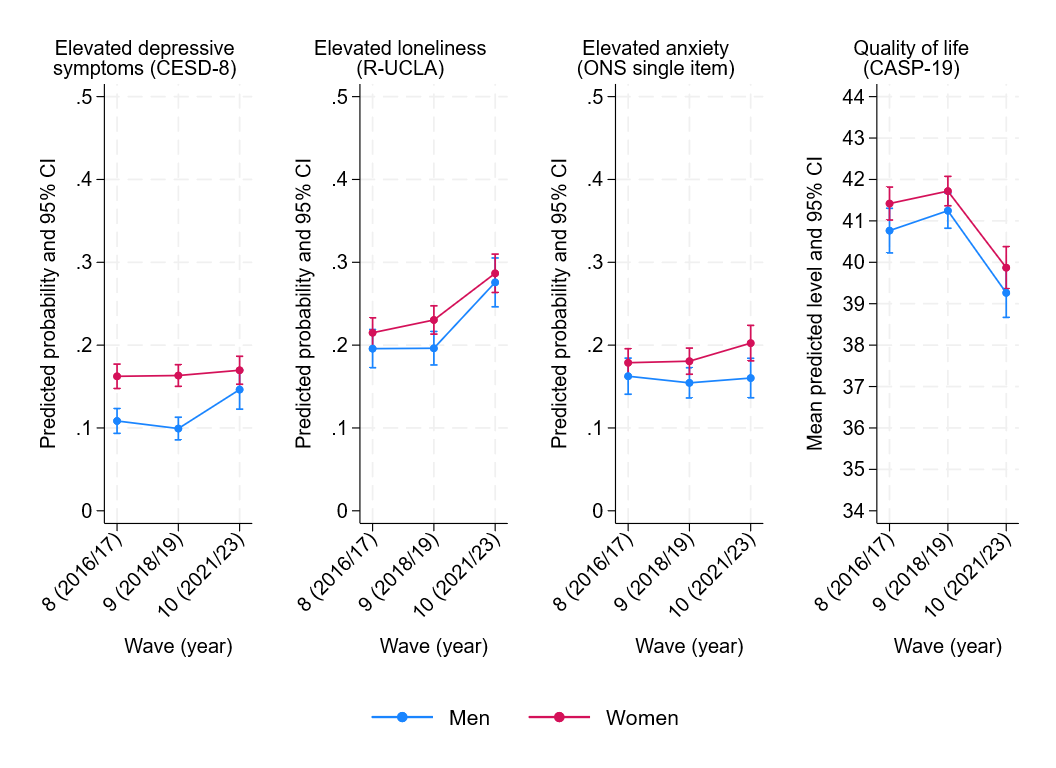


# Figure S3. Marginal predicted probability of mental (ill-)health and predicted mean quality of life across time-points (brackets represent 95% confidence intervals) by living situation (living alone / not living alone) after excluding participants interviewed during the temporary restrictions between November 2021 and February 2022, using a non-face-to-face mode, or without a COVID-19 booster vaccine.


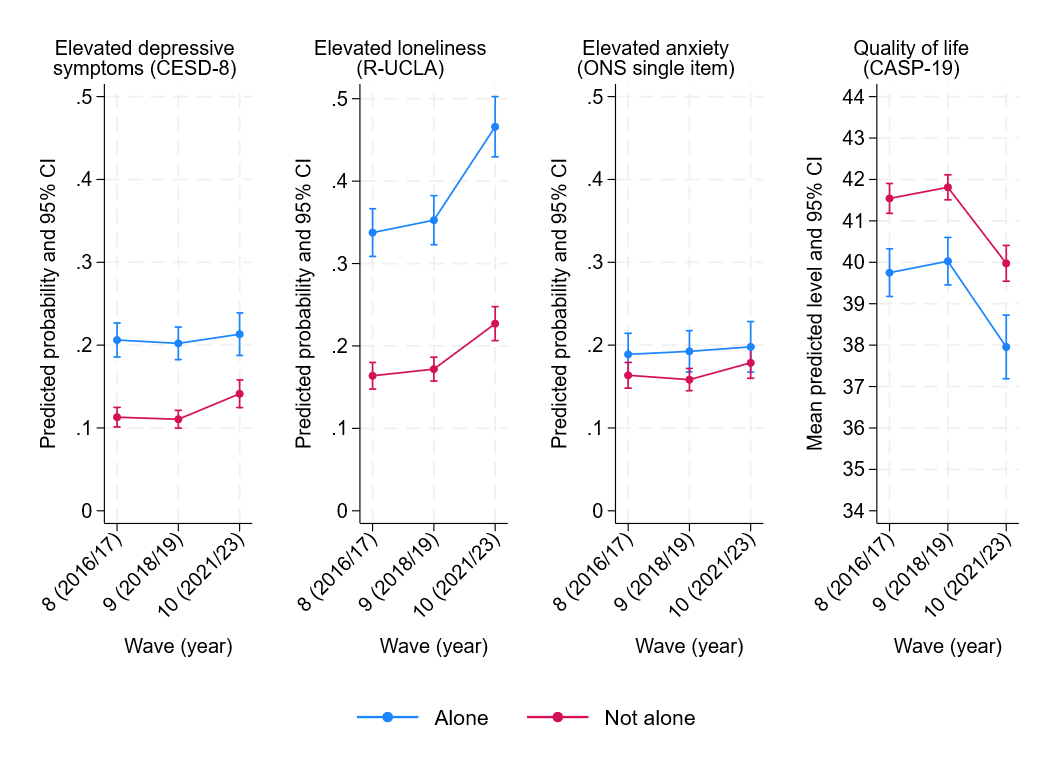


# Figure S4. Marginal predicted probability of mental (ill-)health and predicted mean quality of life across time-points (brackets represent 95% confidence intervals) by wealth quintiles after excluding participants interviewed during the temporary restrictions between November 2021 and February 2022, using a non-face-to-face mode, or without a COVID-19 booster vaccine.


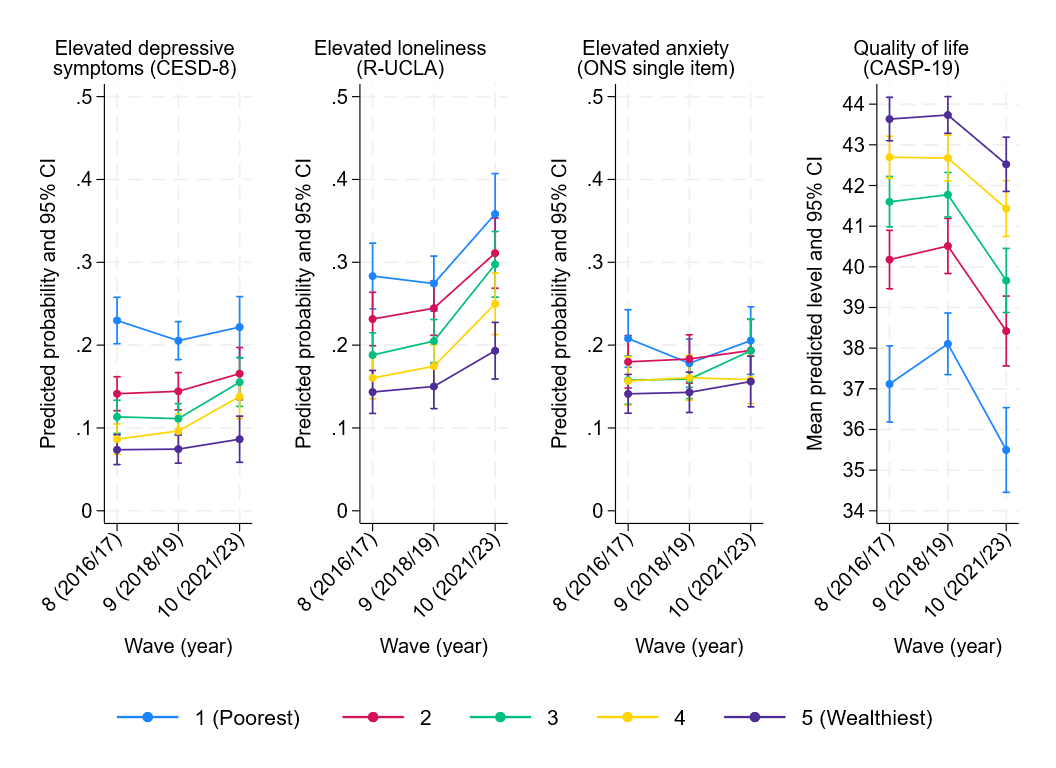


# Figure S5. Marginal predicted probability of four different anxiety categories (measured with the UK Office for National Statistics single question) across time-points (brackets represent 95% confidence intervals) from multinomial models.


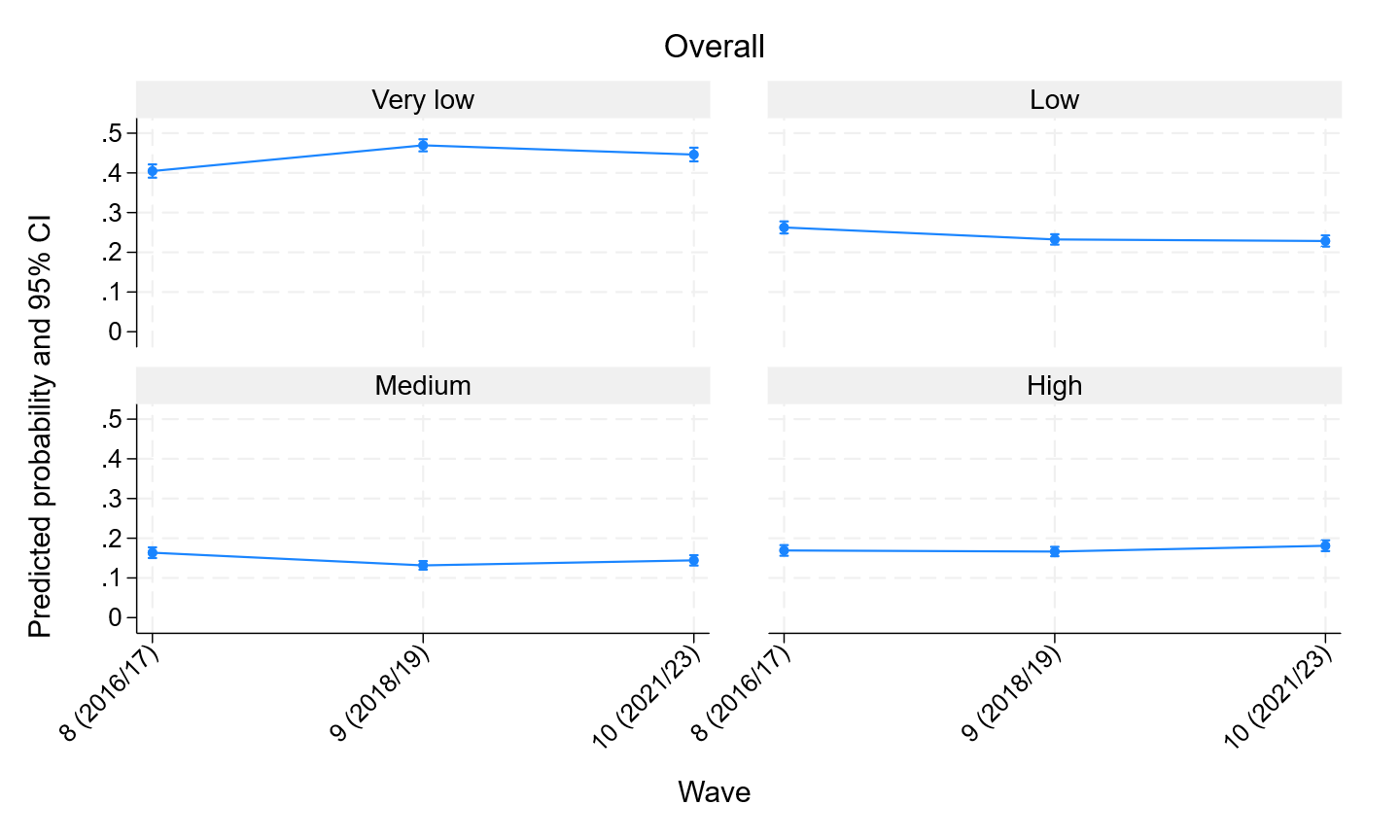


# Figure S6. Marginal predicted probability of four different anxiety categories (measured with the UK Office for National Statistics single question) across time-points (brackets represent 95% confidence intervals) from multinomial models. Results by gender (women/men).


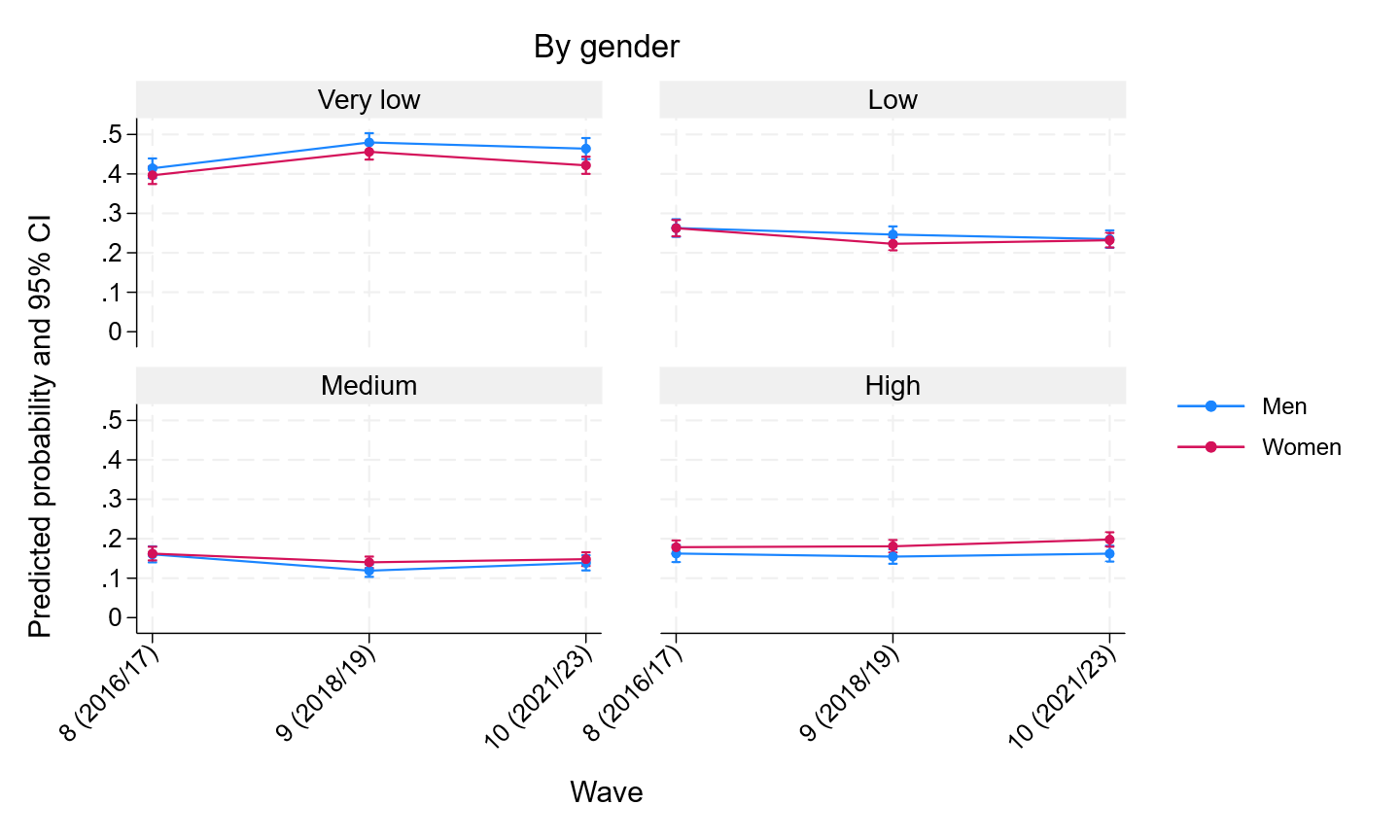


# Figure S7. Marginal predicted probability of four different anxiety categories (measured with the UK Office for National Statistics single question) across time-points (brackets represent 95% confidence intervals) from multinomial models. Results by living situation (living alone / not living alone)


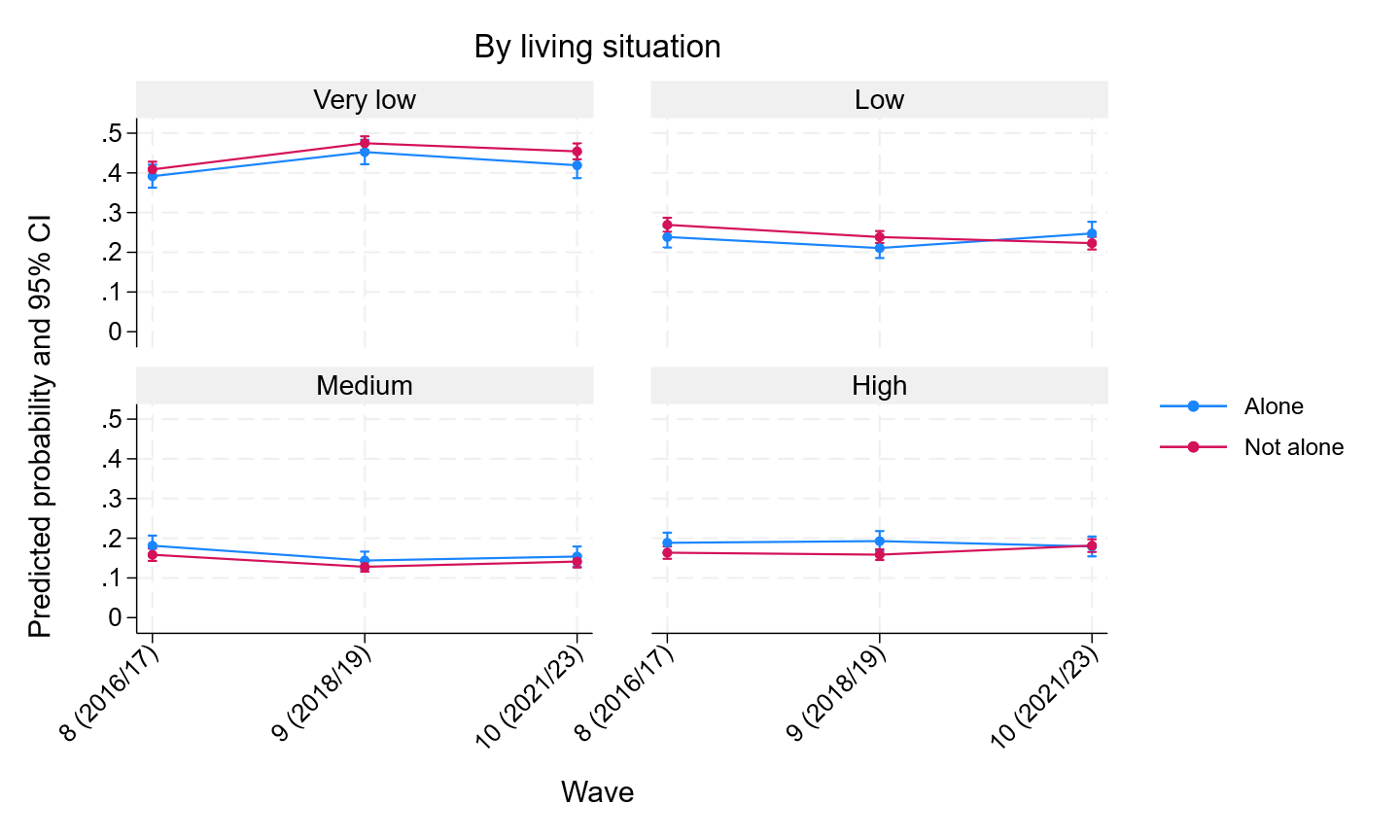


# Figure S8. Marginal predicted probability of four different anxiety categories (measured with the UK Office for National Statistics single question) across time-points (brackets represent 95% confidence intervals) from multinomial models. Results by wealth quintiles. Wave 8: 2016-2017; wave 9: 2018-2019; wave 10: 2021-2023.


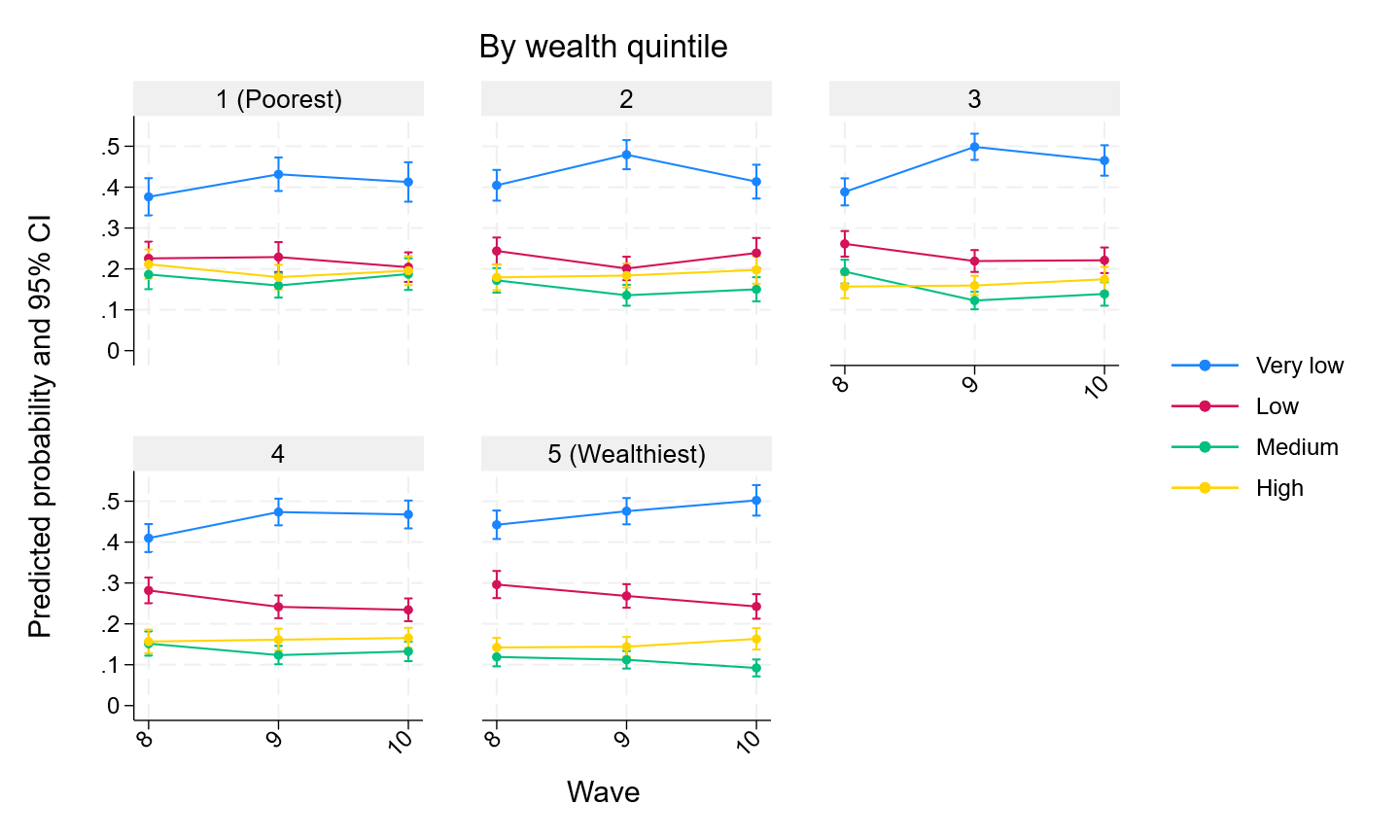


# Figure S9. Marginal predicted probability of mental (ill-)health and predicted mean quality of life across time-points (brackets represent 95% confidence intervals). Results by age groups (50-69 and 70+ year-olds).


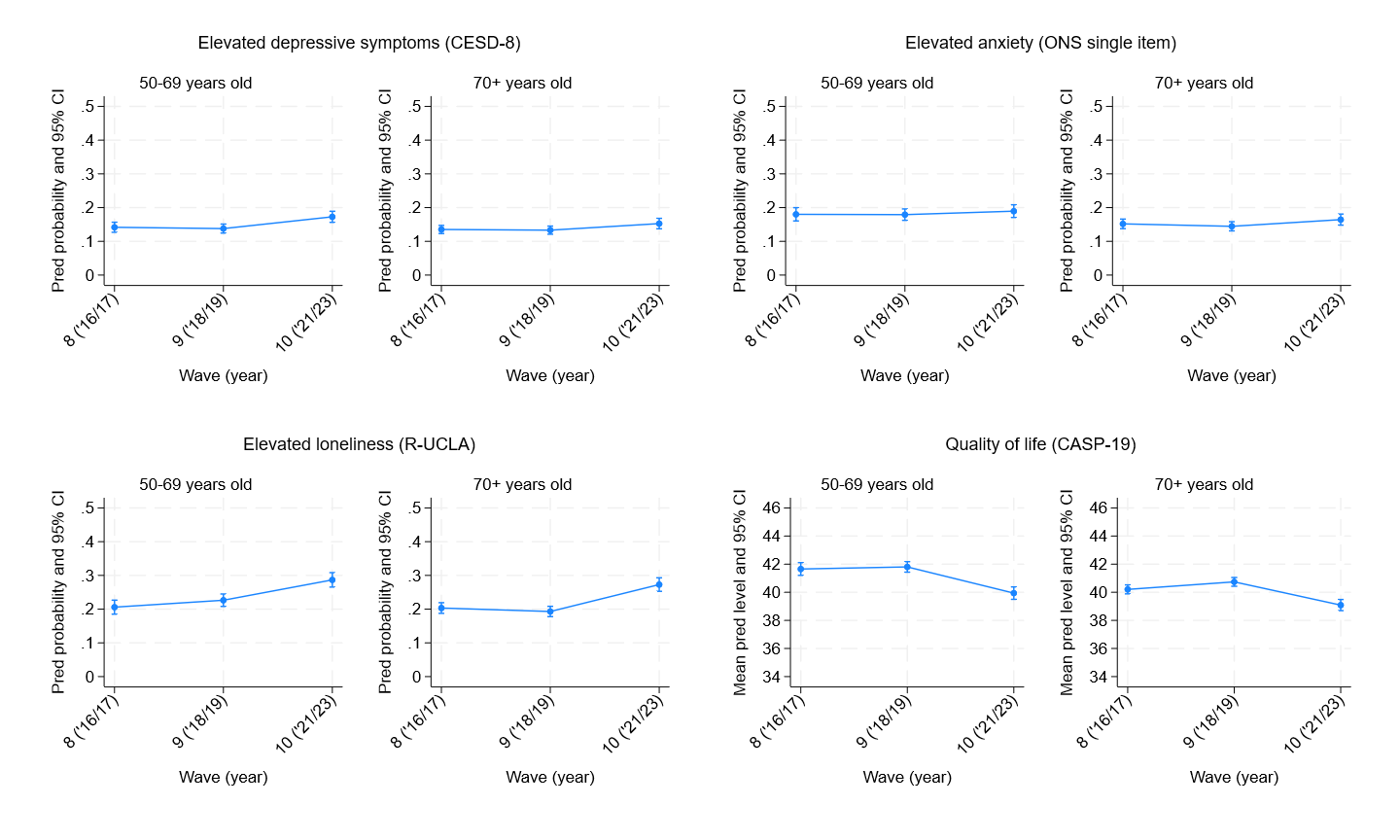


# Figure S10. Marginal predicted probability of mental (ill-)health and predicted mean quality of life across time-points (brackets represent 95% confidence intervals). Results by age groups (50-69 and 70+ year-olds) and gender (women / men).


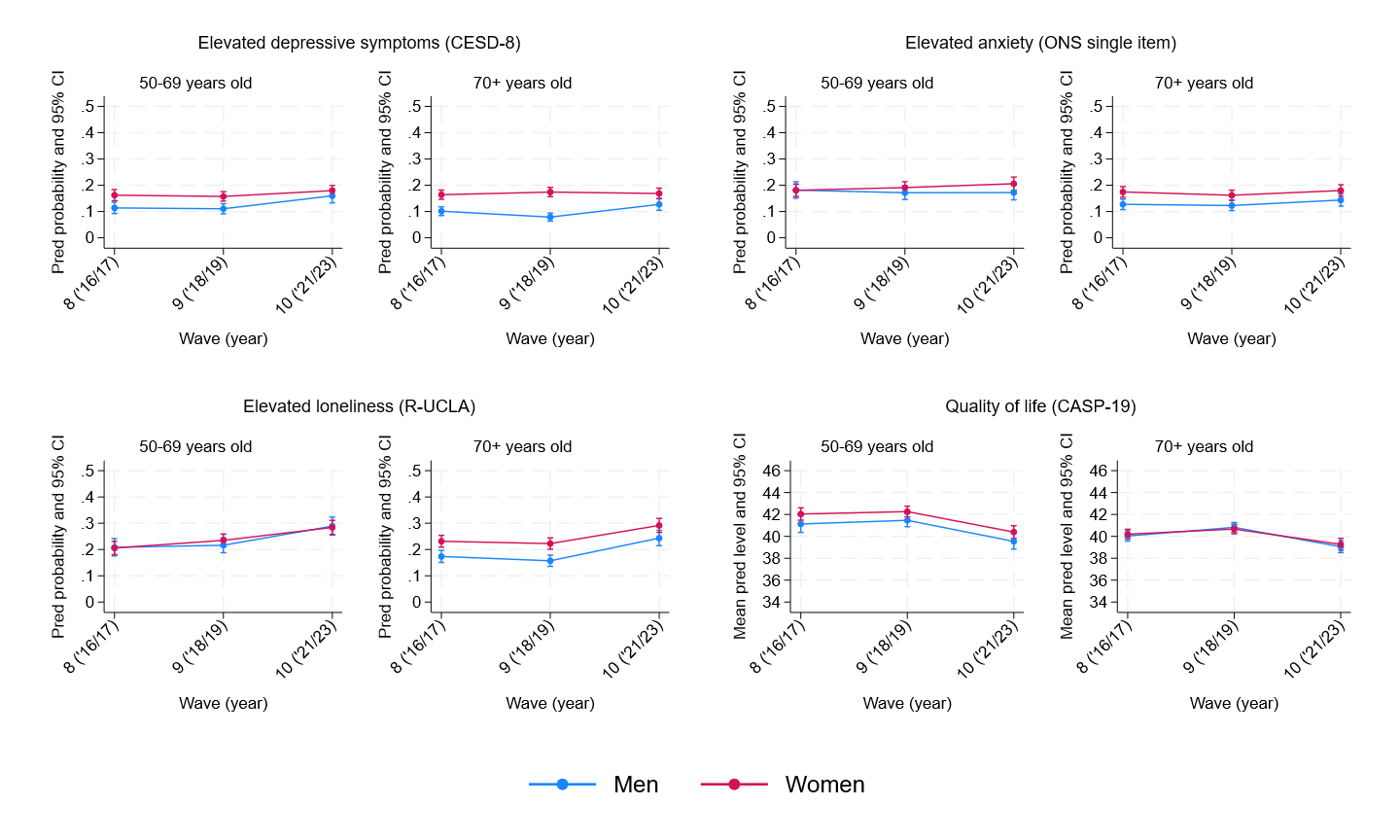


# Figure S11. Marginal predicted probability of mental (ill-)health and predicted mean quality of life across time-points (brackets represent 95% confidence intervals). Results by age groups (50-69 and 70+ year-olds) and living situation (living alone / not living alone).


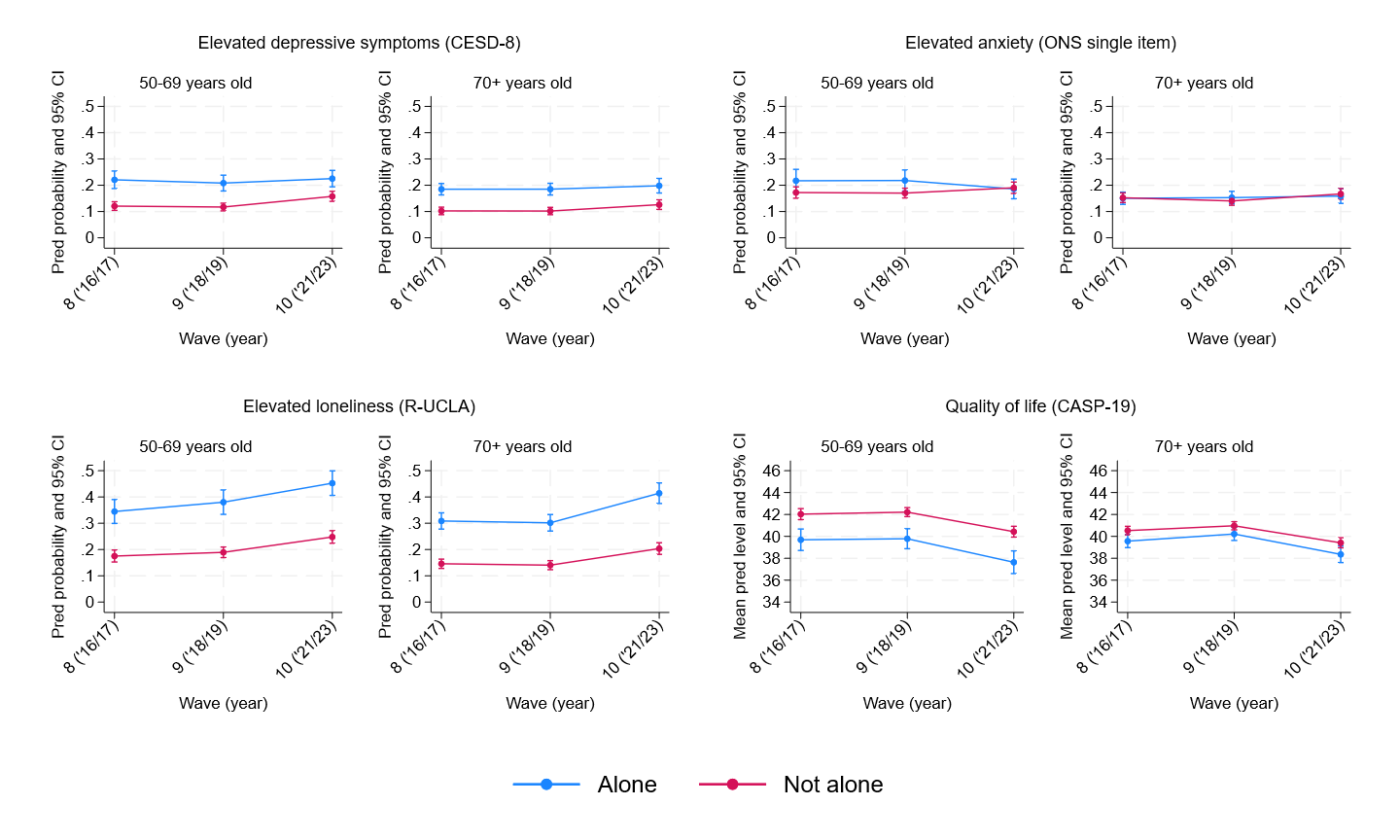


# Figure S12. Marginal predicted probability of mental (ill-)health and predicted mean quality of life across time-points (brackets represent 95% confidence intervals). Results by age groups (50-69 and 70+ year-olds) and wealth quintiles.


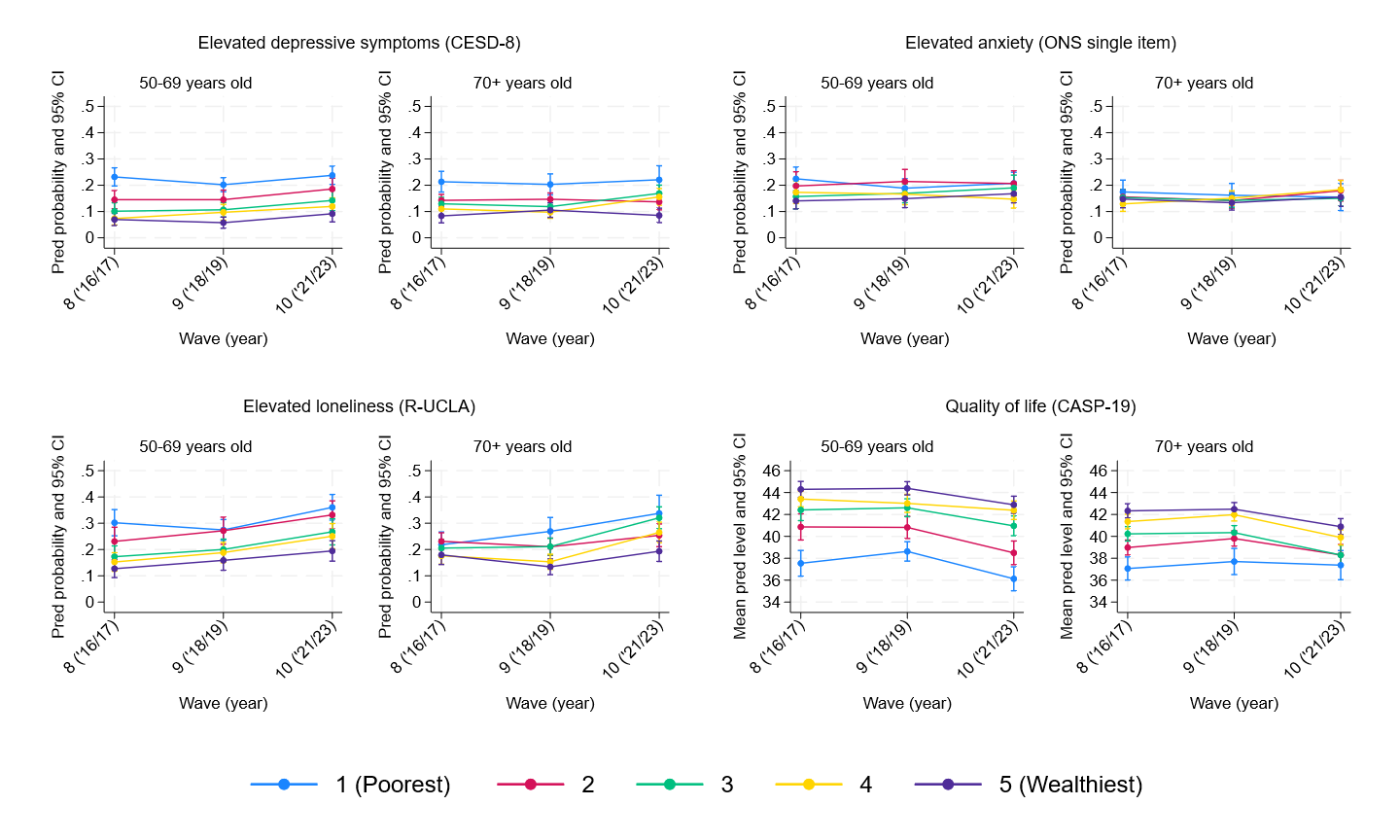

Supplement: Supplementary [file EMS213667-supplement-Supplementary.docx]
